# Hierarchical and programmable one-pot synthesis of oligosaccharides

**DOI:** 10.1038/s41467-018-07618-8

**Published:** 2018-12-06

**Authors:** Cheng-Wei Cheng, Yixuan Zhou, Wen-Harn Pan, Supriya Dey, Chung-Yi Wu, Wen-Lian Hsu, Chi-Huey Wong

**Affiliations:** 1 Bioinformatics Program, Taiwan International Graduate Program, Academia Sinica, Taipei, 11529 Taiwan; 2 Institute of Information Science, Academia Sinica, Taipei, 11529 Taiwan; 3 Institute of Biomedical Informatics, National Yang-Ming University, Taipei, 11221 Taiwan; 4 Genomics Research Center, Academia Sinica, Taipei, 11529 Taiwan; 5 Institute of Biomedical Sciences, Academia Sinica, Taipei, 11529 Taiwan; 6 Department of Chemistry, The Scripps Research Institute, La Jolla, CA 92037 USA

## Abstract

The programmable one-pot oligosaccharide synthesis method was designed to enable the rapid synthesis of a large number of oligosaccharides, using the software Optimer to search Building BLocks (BBLs) with defined relative reactivity values (RRVs) to be used sequentially in the one-pot reaction. However, there were only about 50 BBLs with measured RRVs in the original library and the method could only synthesize small oligosaccharides due to the RRV ordering requirement. Here, we increase the library to include 154 validated BBLs and more than 50,000 virtual BBLs with predicted RRVs by machine learning. We also develop the software Auto-CHO to accommodate more data handling and support hierarchical one-pot synthesis using fragments as BBLs generated by the one-pot synthesis. This advanced programmable one-pot method provides potential synthetic solutions for complex glycans with four successful examples demonstrated in this work.

## Abstract (web summary)

The software Optimer has aided the programmable one-pot oligosaccharide synthesis with a library of 50 Building BLocks (BBLs). Here, the authors expanded Optimer's validated and virtual libraries of BBLs and developed Auto-CHO, a software which allows the one-pot programmable synthesis of more complex glycans.

## Introduction

Most of human proteins and natural products are glycosylated^1,2^. However, it has not been clear what roles carbohydrates have in biological molecules, mainly due to the presence of hard-to-separate glycosylated mixtures in the biological system and the difficulty encountered in the synthesis of individual glycosylated molecules. Though the synthesis of oligosaccharides with chemical or enzymatic approach has been considered to be a mature methodology^3^, it is still limited to laboratories specialized in the field because of the tedious trial-and-error labor of intermediate separation and protecting group manipulation. The programmable one-pot synthesis method was developed to tackle this problem and was based on the sequential use of thioglycoside BBLs to form glycosidic bonds according to the reactivity differences of the BBLs^4^. It is performed by a sequential addition of thioglycoside BBLs according to the RRV, starting from the most reactive one from the non-reducing end unit toward the reducing end unit. The reactivity of each BBL can be tuned by protecting groups leaving one or more exposed OH groups to react with its donor. There are around 20 protecting groups available currently, and a combinatorial choice of these protecting groups for each mono-, di- or trisaccharide building block gives a wide-range of BBLs with different RRVs. Since the selection of suitable BBL combinations for the synthesis of a desired oligosaccharide in high yield represents a major challenge, a program called Optimer was developed for identifying the best combination of BBLs based on RRVs^4^.

To use the Optimer program, the user will first input the desired glycan structure, then one or more synthetic methods with appropriate combinations of BBLs needed to generate the target glycan are displayed and ranked according to the RRV of each BBL. The user can decide which combination of BBLs to use, based on their knowledge and the availability of BBLs.

The original Optimer program could only synthesize small oligosaccharides due to the RRV ordering requirement, and the limited number of BBLs. To make glycan synthesis more versatile and applicable to more complex oligosaccharides as well as available to the research community, we develop a software called Auto-CHO to meet the challenges encountered in the original Optimer program.

## Results

### Auto-CHO

**Fig. 1:**
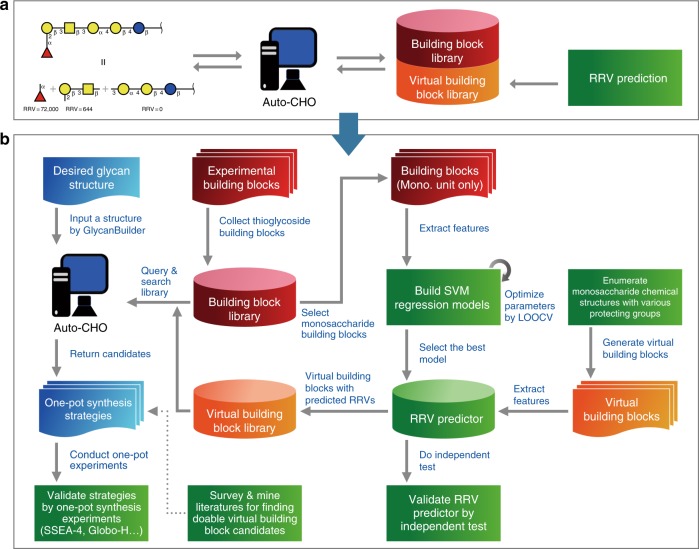
The workflow of Auto-CHO program. **a** The basic concept of Auto-CHO. **b** The detailed workflow. Auto-CHO allows users to input a desired glycan structure and the program returns with one-pot glycan synthesis options. To facilitate the ability of Auto-CHO, we have not only expanded the thioglycoside BBLs but also constructed a virtual BBL library by enumerating monosaccharide structures with different protecting group combinations and theoretical RRVs that are estimated by our RRV predictor. RRV predictors are trained by SVM regression models, and features from experimental BBLs with known RRVs. The predictor with the best performance by leave-one-out cross-validation (LOOCV) has been chosen as the final model. Independent test has also been applied to validate the result. Since the use of virtual BBL in glycosylation may not have been validated and it is uncertain if it can be successfully used in the one-pot synthesis process, text mining could be used to identify those virtual BBL candidates that have been reported in literature and thus, have a good chance of participating in the one-pot synthetic reaction. The synthetic methods provided by Auto-CHO have been further validated by four synthetic experiments in this study

The software Auto-CHO contains three unique features: (1) the program can be operated by graphical user interface and is a cross-platform with Java Runtime Environment; (2) it provides more synthetic BBLs with validated RRVs, and virtual BBLs with accurately predicted RRVs to greatly expand the current library size; (3) the program can be used to guide the one-pot synthesis of more complex oligosaccharides through fragment coupling. Auto-CHO can break a tree-shaped target glycan structure into fragments, provide synthetic routes to each fragment by the one-pot method, and put the fragments together also through the one-pot approach with the same or different leaving groups. Figure 1 shows the workflow of this method, in which Auto-CHO produces tens of thousands of virtual BBLs with accurately predicted RRVs through machine learning. Examples are shown in Supplementary Figure 1 and Supplementary Note 1.

We first increase the library size from 50 to 154 real BBLs with verified RRVs (Supplementary Data 1–7). Then, based on machine learning from these RRVs, create more than 50,000 virtual BBLs with predicted RRVs (Supplementary Data 8), making possible the synthesis of a diverse array of glycans. Although the structures of these virtual BBLs may not have been synthesized previously, most of them were used in glycosylation reactions with different leaving groups, so we can leverage many of the existing BBLs with different leaving groups which have been used for glycosylation reactions and convert them to thioglycosides or incorporate them into the program directly to increase the number of potentially feasible combinations. It is noted that the library search is operated in user’s local machine, and all query structures are in private.

Just like the original Optimer program, the Auto-CHO program cannot guarantee the success of high-yield synthesis for every target using the combinations of BBLs suggested by the program, since there are many other structural constraints or steric hindrance involved in the actual chemical reactions that may be difficult to predict by in silico simulation. However, Auto-CHO, an algorithm for hierarchical one-pot synthesis, has created more potentially feasible combinations by its virtual BBLs with accurately predicted RRVs, acting much like a search engine.

### RRV prediction and validation

In the following, we describe the process of RRV prediction (Table 1). First of all, there could be many factors affecting the RRV of a BBL. According to Steve Ley et al.^5^, NMR chemical shifts are related to the RRV of a BBL. Different protecting groups can affect RRVs, and BBLs with different protecting groups should have different structures and chemical shift values. Therefore, we collect monosaccharide BBLs based on hexose (Hex), *N*-acetyl-hexose (HexNAc), and sialic acid (SA) sugar types for RRV prediction. Since it is time-consuming to measure the NMR chemical shifts of each BBL at each position by experiments, we extract the ^1^H-NMR and ^13^C-NMR chemical shifts of BBLs calculated by ChemDraw^6, 7^, which exhibits no significant difference with experimental chemical shifts (see Supplementary Note 2 and Supplementary Data 9), and use them as features incorporated with other basic properties to build a RRV predictor by SVM regression model (Methods). The performance by leave-one-out cross-validation (LOOCV, see Supplementary Note 3) shows that chemical shifts alone can give the prediction result with Pearson correlation coefficient (PCC) of 0.70 and the mean absolute error (MAE) of 7520, though this performance may not be good enough for practical application. See Supplementary Note 4 for the definitions of performance evaluation.

**Table 1:** LOOCV performances of RRV predictors with different sugar classes, feature types, or settings

Dataset size	Sugar class	Feature type	FS	Feature size	PCC	MAE	RAE
136	Hex, HexNAc, SA	BP + CS (Norm.)	No	25	0.6964	7519.60	0.6161
117	Hex, HexNAc	BP + CS (Norm.)	No	16	0.6803	4220.57	0.4428
117	Hex, HexNAc	BP + CS (Bina.)	No	131	0.7675	3876.85	0.4067
117	Hex, HexNAc	BP + CS (Bina. + Norm.)	No	144	0.7768	3868.84	0.4059
117	Hex, HexNAc	MD	No	1444	0.7326	4131.05	0.4334
117	Hex, HexNAc	BP + CS + MD	No	1595	0.8803	2537.81	0.2662
117	Hex, HexNAc	BP + CS + MD	Yes	222	0.9706	1291.32	0.1355
**117**	**Hex, HexNAc**	**BP** **+** **CS** **+** **MD**	**Yes**	**225**	**0.9701**	**1253.31**	**0.1315**

The optimized performance is shown in bold

*FS* Feature selection; *BP* basic properties; *CS* (calculated) chemical shifts; *MD* molecular descriptors; *Norm.* normalized real values; *Bina.* binarization (Methods)

Since the RRVs of SA BBLs vary and its nine-carbon backbone structure is different from Hex or HexNAc, which has a six-carbon backbone, we remove the SA BBLs from the dataset for RRV prediction. As a result, the MAE is reduced from 7520 to 4221. Next, we use normalization combined with binarization of certain features to improve the PCC to 0.78 and the MAE to 3869.

Furthermore, to capture the maximum amount of chemical information encoded within a BBL, both chemical properties and mathematical procedures may be used to transform the information into an array of molecular descriptors, i.e., useful numbers which mimic the results of certain standardized experiments^8^. It has been documented that molecular descriptors can be used to solve the quantitative structure activity relationship (QSAR) problem, such as predicting the activities of chemical compounds^9^. Thus, molecular descriptors are also employed for RRV prediction. We use PaDEL-Descriptor^9^ to generate 1D and 2D molecular descriptors (as features) from the structures of BBLs, and use them alone to achieve the performances with PCC of 0.73 and MAE of 4131.

By combining the basic properties (e.g., sugar type, the anomeric configuration of product, etc.), NMR chemical shifts, and molecular descriptors together, the PCC is increased to 0.88 and the MAE decreased to 2538. Finally, we adopt a feature selection strategy (by removing the least effective feature one at a time) to further improve the PCC to 0.97 and the MAE to 1253 with 225 selected features. The relative absolute error (RAE) of the final model is 0.1315.

To eliminate the possible doubt of overfitting, we adopt the popular 10-fold cross-validation to evaluate the result of feature selection and compare the performance of each set of feature to see the effects of the number of selected features (e.g., 222 is more than the number of BBLs in the training set; 100, 75, 50, and 25 are less than the number of BBLs in the training set). This evaluation would avoid seeing the data from the test part in the feature selection process. The result (Supplementary Table 1a and Supplementary Data 10) shows that there is no significant difference between 222 (selected feature size > training set size) and 100 (selected feature size < training set size). Although PCC decreases and MAE increases when the number of selected feature decreases, the change is not significant. Thus, we believe that even the final size of the selected features (225) is bigger than the size of the training size (117) in our case, it would not have the overfitting problem and the developed RRV predictor can be used for real application, as supported by the result of independent test (Table 2). Note that the result with 222 selected features in Table 1 has better performance than the result with 222 selected features in the Supplementary Table 1a. It is because the latter is evaluated by 10-fold cross-validation for feature selection, in which only 9/10 of the training set is used for feature selection rather than the entire training set as in LOOCV. In general, LOOCV is expected to have better performance than 10-fold cross-validation for small training set.

**Table 2:** Predicted and observed RRVs of representative virtual building blocks

Supplementary Data 11 shows details of LOOCV performance and Supplementary Data 12 shows the details of  the selected features used in our optimized RRV predictor. According to the characteristic of the programmable one-pot glycan synthesis, the order of RRVs should be high, medium, and low. Based on our experience, at most three or four BBLs can be used to carry out the one-pot process to avoid side reactions caused by the activator. Thus, we classify all RRVs into three categories, [>15,000], [1000–15,000], and [0–1000] with the classification accuracy around 0.97. Among the BBLs selected for machine learning, the numbers of BBLs with RRV [>15,000], [1000–15,000], and [0–1000] are 19, 21, and 77, respectively; and the corresponding PCC values for these three categories are 0.89, 0.94, and 0.89, respectively. The differences in absolute numbers between observed and predicted RRVs are relatively big, especially when RRV is high (RRV > 15,000); however, if the observed and predicted RRVs belong to the same class of sugars, the predicted RRV still provides precious information for use in the programmable one-pot glycan synthesis (Supplementary Table 1b and Supplementary Data 11).

We selected some virtual building blocks for experimental validation and the results show that both the predicted and observed RRVs are similar (Table 2). The protecting group 3 of Dx7 is Fmoc, which does not appear in our training set. The observed RRV is 1313 and the predicted one is 971, indicating that the RRV prediction is quite successful. Although Dx6 has a large difference between the observed and predicted RRVs, the ratio of the observed/predicted RRV is not very significant, probably due to some unknown factors affected by the PMB group.

Overall, we generated more than 50,000 virtual BBLs (Methods), including Gal, Glc, Man, GalNAc, and GlcNAc sugar types, with predicted RRV based on the optimized RRV predictor.

### Representative one-pot synthesis of oligosaccharides

With Auto-CHO and BBLs available, we then conduct the synthesis of four representative oligosaccharides as protected forms, including Globo-H, heparin pentasaccharide, and LacNAc repeats using the BBLs reported previously, and stage-specific embryonic antigen 4 (SSEA-4) described in this study. All BBLs for the one-pot synthesis of these representative oligosaccharides are now included in the library of Auto-CHO and can be identified by users for the one-pot synthesis through the program search described here. The following briefly demonstrates the operation of each example.

**Fig. 2:**
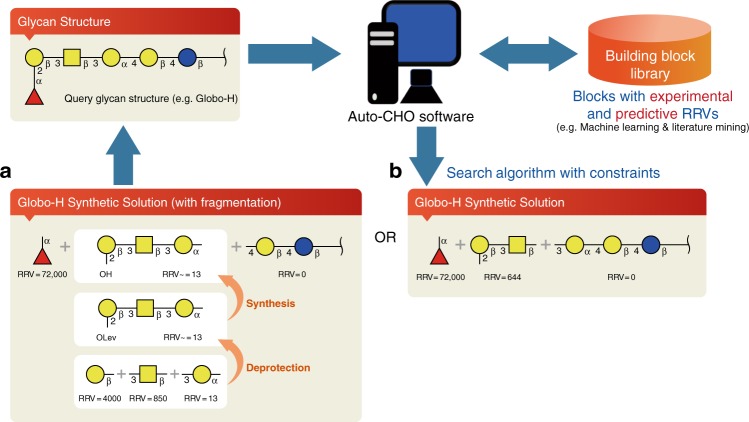
Illustration of the programmable one-pot synthesis of Globo-H by Auto-CHO. **a** The synthetic solution shows [1 + 3 + 2] strategy. The internal fragment can be synthesized by another one-pot approach. **b** The synthetic solution shows [1 + 2 + 3] strategy without further fragmentation

Globo-H is a glycosyl ceramide specifically found in a variety of epithelial tumors such as colon, ovarian, gastric, pancreatic, endometrial, lung, prostate, and breast cancers, but not on the immune-accessible normal tissues^10–14^. The Globo-H hexasaccharide has been used as an antigen for the development of carbohydrate-based vaccines against breast cancer and prostate cancer^15^, and a positive phase II clinical trial result has been released^16^. This glycan was prepared previously^17^ using the [1 + 3 + 2] strategy (Fig. 2a), but the BBL used to form the axial glycosidic linkage generates a low-yield product. To overcome this problem, we have developed a strategy based on the [1 + 2 + 3] strategy (Fig. 2b)^18^, which gave the product with more than 80% yield.

To conduct the programmable one-pot synthesis of Globo-H using the Auto-CHO program, two possibilities are suggested by the program. The first method (Fig. 2a) shows that the query glycan structure can be synthesized by three fragments. Fragment-1 consists of only one fucose BBL with RRV 72,000. Fragment-2 is a trisaccharide component that can be synthesized by the one-pot approach with three monosaccharide BBLs, including galactose, *N*-acetylgalactosamine, and galactose types with RRV 4000, 850, and 13, respectively. After completion of the one-pot synthesis of fragment-2, the Lev group at position-2 of the second galactose is deprotected, and the RRV of the deprotected fragment-2 is determined as 13. The calculated yield of fragment-2 is 71%. Fragment-3 is a disaccharide reducing end acceptor, and its RRV is zero. We followed the suggestion to carry out the synthesis, and the overall experimental yield of this fragment-based one-pot synthesis was 62%. The second method suggests the use of [1 + 2 + 3] strategy as shown in Fig. 2b which is an improved process to avoid the low-yield alpha-glycosylation to generate the axil glycosidic bond between the second and the third fragments. Based on Auto-CHO, the predicted overall yield of this procedure is 97%, and Globo-H was indeed prepared by this improved method in more than 80% yield^18^. Though the syntheses of Globo-H were performed previously, the Auto-CHO program further confirms its reliable operation.

Based on our experience, the synthesis of an oligosaccharide should not be performed with more than three BBLs directly by the programmable one-pot strategy, because the promotor *N*-iodosuccinimide used in the reaction often caused a side reaction with the oxocarbenium intermediate to form a dead end product. In this case, Auto-CHO will suggest the use of fragments for the one-pot reaction and each fragment can be synthesized from monosaccharide BBLs with the one-pot approach first and then used, after unmasking the protected hydroxyl group, as acceptor in another one-pot reaction to form the target structure.

**Fig. 3:**
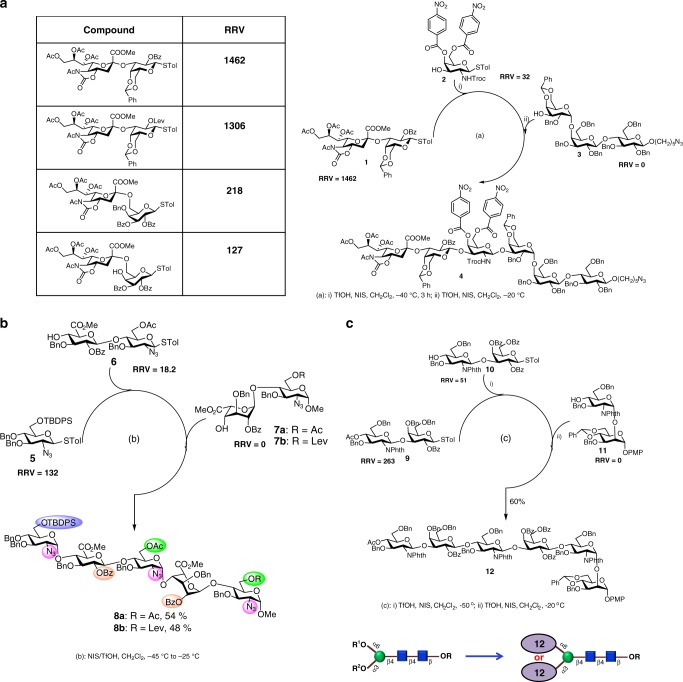
Examples of programmable one-pot synthesis. **a** Auto-CHO suggests that SSEA-4 can be synthesized with three BBLs: sialyl disaccharide **1** with RRV = 1462, monosaccharide **2** with RRV = 32.0, and reducing end acceptor **3** with RRV = 0. The calculated overall yield is 94% and the experimental yield is 43%. **b** Synthesis of a heparin pentasaccharide with differential protecting groups in color to allow selective introduction of the sulfate groups. **c** Synthesis of the oligoLacNAc module for the assembly of *N*-glycans with LacNAc repeats

SSEA-4 is a pluripotent human embryonic stem cell marker and its expression is correlated with the metastasis of some malignant tumors, thus it is regarded as a cancer-specific glycolipid^11, 12^. This oligosaccharide contains an alpha-2,3-linked sialic acid residue at the non-reducing end. However, sialic acid cannot be used as the first BBL because it is the least reactive BBL and contains a quaternary anomeric center which causes hindrance and elimination in the glycosylation reaction. To solve these problems, we have designed a series of sialyl disaccharides as BBLs of which the RRV is determined by the reducing end sugar (such as the one in Fig. 3a). Based on the RRVs of sialyl disaccharides, a [2 + 1 + 3] one-pot reaction for the synthesis of SSEA-4 has been chosen from the Auto-CHO program, and using this strategy, SSEA-4 was prepared in 43% yield. This programmable method for the synthesis of SSEA-4 is, in our opinion, better than the orthogonal method reported previously^19^.

The heparin pentasaccharide with regiodefined sulfate patterns has been used as anti-coagulant and prepared previously in our laboratory using the programmable one-pot program^20^. In order to develop a better method to allow the flexibility to install the sulfate groups’ regioselectively, we have redesigned building blocks and reported the successful synthesis of heparin pentasaccharides for the regioselective introduction of the sulfate group^21^. These building blocks are now in the library of the Auto-CHO program. To conduct the programmable one-pot synthesis of heparin pentasaccharides with various sulfation patterns, we use Auto-CHO to identify the [1 + 2 + 2] one-pot strategy as shown in Fig. 3b for the synthesis of a protected heparin pentasaccharide using monosaccharide **5** (RRV = 132), disaccharide **6** (RRV = 18.2) and reducing end disaccharide **7** (RRV = 0) as BBLs to obtain the protected pentasaccharide **8** in 48% yield (an increase of 28% from the previous one^20^). Glycan **8** with protecting groups can now be differentially deprotected for the installation of the desired sulfate groups’ regioselectively to explore their functional effects.

The oligomer of *N*-acetyllactosamine (OligoLacNAc) is often found on the N-linked glycans of glycoproteins associated with diseases and it has an important role in intercellular recognitions. Recently, we have developed a modular approach to the synthesis of N-glycans using designed modules and the core trisaccharide of N-glycans^22^, and compound **12** as well as others was shown to be an effective key building block for the synthesis of various N-linked glycans containing oligoLacNAc^22^. To test the use of Auto-CHO for the programmable one-pot synthesis of **12**, we follow the guide of Auto-CHO to identify the BBLs for the one-pot synthesis of compound **12** using donor **9** (RRV = 263) coupled with acceptor **10** (RRV = 51), followed by addition of another acceptor **11** (RRV = 0) to give the oligo-*N*-acetyllactosamine **12** in 60% overall yield (Fig. 3c). With this Auto-CHO available, various multiantennary N-glycans containing the LacNAc repeats at different branches can be efficiently assembled.

## Discussion

In summary, we have developed the Auto-CHO program as an advanced method for the one-pot synthesis of oligosaccharides. In addition to experimental validation, the RRV of BBLs can be accurately predicted by their chemical structures with the machine learning approach. Therefore, we have expanded our current library by adding more BBLs with verified RRVs and virtual BBLs with predicted RRVs. The Auto-CHO software program provides a valuable guideline for BBL selection to conduct the programmable one-pot synthesis of oligosaccharides. It also provides a hierarchical blueprint for the multiple one-pot synthesis of more complex glycans through fragment coupling. Furthermore, we have successfully demonstrated the one-pot synthesis of four glycans guided by the Auto-CHO program. With the Auto-CHO program available to the research community and the input from the users to validate the predicted BBLs with experimental RRVs, the program is expected to become a useful choice for the synthesis of oligosaccharides. In addition to the automated solid-phase method developed by Seeberger et al.^23^ and other chemical and enzymatic methods developed by many laboratories^3, 24, 25^, we anticipate that, with the Auto-CHO program and library available to the research community, the goal of oligosaccharide synthesis may be realized to facilitate the study of glycans and their roles in glycoproteins and glycolipids.

## Methods

### Computational method

The computational methods described here include two parts, Algorithm and RRV Prediction. For the algorithm part, we describe how to search the target glycan against the BBL library. It defines glycan data structure and matched cases (e.g. perfectly or precursor matches) between the target glycan and BBLs. We also describe how to conduct a search with or without fragment(s), and how to connect fragments together. For the prediction of RRV, we describe how we prepare the training set and features (NMR chemical shifts and other properties) for machine learning, including feature extraction, feature encoding, data transformation and rescaling, followed by evaluation. Finally, we use the optimized RRV predictor to build a virtual BBL library, which can be used in the search procedure.

### Algorithm

The GlycanBuilder^26, 27^ embedded in Auto-CHO allows users to input a desired glycan structure by mouse drawing. The program takes the given structure to search against the BBL library and returns with suitable synthetic solutions. The information of the query glycan structure, including the sugar type of each monosaccharide unit and their anomeric linkage configuration in the glycan should be given by the user. The user should define the fragment to be used as either a BBL in the library with full protection or as an intermediate product synthesized through the one-pot program followed by selective deprotection for further use. To synthesize some complex glycans we often need to first prepare some fragments as intermediates and use these fragments or selectively deprotected fragments as BBLs for further one-pot synthesis.

### The record of experimentally validated BBLs

We have collected 154 thioglycoside BBLs with experimentally validated RRVs. Each BBL consists of one or multiple monosaccharide units with certain or all hydroxyl groups exposed or protected. The record of a BBL includes its IUPAC name, chemical structure and anomeric linkage configuration, RRV, index, and saccharide elements (monosaccharide units of each BBL). For each saccharide element, the sugar type, anomeric form (alpha or beta form), identifier (ID), parent identifier (PID), and the hydroxyl groups with substituents and positions are recorded (Supplementary Figure 2).

### Glycan data structure description

Supplementary Figure 3a shows the glycan data structure used in this research. Definitions are described as follows. Root residue: the first monosaccharide of a glycan structure from the reducing end. Current state: the moving state in algorithm when searching against the BBL library. The residue number in the current state is equal to the residue number in the matched BBL. Current residue: residue(s) in the current state. Child residue of the current state: the residue(s) of which the parent residue is in the current state. Sub-structure of the current state: the partial glycan structure of which the parent residue is in the current state. Leaf residue: residue(s) that has no child residue.

### Matched cases between BBLs and the search target

Perfectly matched BBL means that the BBL perfectly matches the glycan structure in the current state of the query structure. The position of the free hydroxyl group on the BBL also perfectly matches the corresponding linkages in the current state of the query structure. For example, if the matched residue(s) of the query structure has (have) no child residue, the BBL should be fully protected; if the matched residue(s) of the query structure has (have) child residue(s), the substituent(s) on the corresponding position(s) of the BBL should be a free hydroxyl group(s).

On the other hand, the precursor matched BBL means that the BBL matches the glycan structure in the current state of the query glycan. However, the linkage between current state and sub-structure on the corresponding positions of the BBL could be masked by a unique protecting group. This protecting group should be deprotected with additional experimental operations. For example, if the matched residue(s) of the query structure has (have) one child residue, the BBL should be fully protected; if the matched residue(s) of the query structure has (have) multiple child residues, the BBL can be semi-protected (at least one substituent on the corresponding position is protected) or fully protected. The protecting group(s) on the corresponding position(s) of the BBL should be unique and can be selectively deprotected in the following step (Supplementary Figure 3b).

### Search procedure

The problem of finding the synthetic solutions of a query structure can be broken down into steps, including finding the synthetic solutions for the sub-structures of the query glycan. The order of residue visiting is the same as depth-first search in-order traversal. The algorithm starts from a leaf residue of the query structure. When the current state is on the leaf residue, the program searches the library for perfectly or precursor matched BBLs. These matched BBLs will be recorded in the synthetic candidate list of the current residue. Next, we move the current state to its parent residue. The algorithm considers two situations: (1) the current state has only one child residue or (2) the current state has multiple child residues. We tabulate the detailed procedure of manipulations in these situations in Supplementary Data 13.

### Search procedure without fragment synthesis

Auto-CHO provides a normal search mode for searching the target glycan structure against the BBL library to find possible synthetic solutions with the one-pot approach. The maximal synthesis steps (N-1) can be defined by the user. Generally, N should be less than three or four, otherwise it will be difficult to obtain the desired glycan structure with high overall yield (60–95%) because the promoter *N*-iodosuccinimide would cause side reactions^28^. The program gives all possible solutions to the synthesis options ranked by the computed overall yield in a descending order. Each possible solution has N BBL combinations ordered by descending RRVs. The algorithm does not consider the deprotection of any masked hydroxyl group of BBLs in the synthetic procedure.

### Search procedure with fragment synthesis

The query glycan structure sometimes can be synthesized through fragments. We provide fragment search mode and separate the query structure into two or more fragments automatically. The fragments with proper protecting groups can be prepared separately using Auto-CHO followed by selective deprotection to expose the hydroxyl group for the subsequent fragment-based one-pot synthesis. A fragment should contain 1 to 3 BBLs since too many synthetic steps in a one-pot operation results in a low-yield product (Supplementary Figure 4a). There are three possible cases. Case 1: there is one matched BBL in the fragment, the next BBL could be either a perfectly or precursor matched one; Case 2: there are two matched BBLs in the fragment, the next BBL can be either a perfectly or precursor matched one; Case 3: there are three matched BBLs in the fragment; we should initiate a new fragment and only a precursor matched BBL can be recorded.

### Approach for connecting fragments together

There are two strategies to put fragments together. The first is to use fragments with different leaving groups for each fragment and connect fragments step by step. For example, in the Supplementary Figure 4b, one can deprotect PG1 of fragment-2 and put fragment-1 and fragment-2 together to form product X. We can then deprotect PG2 of fragment 3 and put X and fragment 3 together. The second strategy is that each fragment can be regarded as a new BBL and use the one-pot approach to synthesize these fragments. The BBLs selected for the one-pot synthesis should have the RRVs ranging from large, medium, and zero, respectively. In the Supplementary Figure 4c, depth-first search can give the post-order traversal for fragment connections with different leaving groups easily. For fragment condensation by the one-pot approach, the algorithm finds a major chain (e.g. the longest path from a leaf residue to the root residue shown in red) of the target structure. One can connect fragment-2 and 3 to form X, and fragment 4 and 5 to form Y. We then put fragments 1, X, Y, and 6 together to form the final product. The estimated RRVs of fragment-1, X, Y and 6 should be large, medium, small, and zero, respectively. If there are more than four fragments on the major chain, strategies with different leaving groups and one-pot approach can be used together.

### Record of synthetic candidate in each query structure

The synthesis of a candidate records all possible synthetic solutions for the query structure. The record contains fragments and overall yield. In each fragment, it records fragment yield, BBLs, and type(s) of protecting group(s). Note that there will be only one fragment in each synthetic candidate by the search procedure without fragment synthesis. There is no need to deprotect any protecting group in this situation, and the fragment yield equals the overall yield of the synthetic candidate.

### RRV prediction

Due to the limited number of BBLs with validated RRVs collected in the library, it is difficult to search the library to find synthetic solutions for every oligosaccharide. It is unrealistic to measure the RRV of every BBLs experimentally for all possible combinations of protecting groups. Therefore, we propose a way to enlarge the library size by constructing virtual BBLs with predicted RRVs. We can build an RRV predicting model based on machine learning from certain experimental data and physical/chemical properties. In this research, we use the reported physical data such as NMR shifts and support vector machine as regression model to predict the RRV of numerous virtual BBLs. We have further validated the prediction experimentally for many virtual BBLs and found that the predictions are generally accurate, so we have enlarged the library size successfully to include more than 50,000 BBLs.

### Training set

In the library, we only collect monosaccharide BBLs including the galactose (Gals), glucose (Glcs), mannose (Mans), *N*-acetylgalactosamine (GalNAcs), *N*-acetylglucosamine (GlcNAcs), and *N*-acetylneuraminic acid or sialic acid (Neu5Acs or SA) type for RRV prediction or determination. Since the RRVs of sialic acid BBLs vary and are often used as the reducing end unit, we did not predict their RRVs. Thus, we only use 117 hexose and hexosamine BBLs for the final prediction model construction. The RRV of these BBLs ranges from 1 to 72,000. The summary can be found in Supplementary Table 1c. Since the RRV of SA is too low to be used as the first BBL for the one-pot synthesis of oligosaccharides with SA in the non-reducing end, we use sialyl disaccharide (i.e., the terminal SA linked to the next sugar) as BBL because the RRV of the disaccharide is mainly determined by the second sugar, so the RRV of the disaccharide will be much higher than SA.

### Feature extraction

We extract features from the 2D chemical structures of BBLs for RRV prediction. Three feature categories are used in this study, including basic properties, calculated chemical shifts, and molecular descriptors. Basic properties include sugar type: [Gal, Glc, Man, GalNAc, GlcNAc], sugar class: [Hex, HexNAc], anomeric state: [Alpha, Beta], protecting groups at positions 2, 3, 4, and 6. For calculated chemical shifts, ChemDraw^6, 7^ is used to calculate the ^1^H and ^13^C-NMR chemical shifts for each experimentally validated and virtual BBL. For ^1^H-NMR, we used “frequency = 400 MHz” and “solvent = CDCl_3_” settings in the ChemDraw program. (ChemDraw does not provide these parameter options for ^13^C-NMR). Supplementary Figure 5 shows that ^1^H and ^13^C-NMR chemical shifts of sugar rings (labeled in red boxes, H1, H2, H3, H4, H5, H6-1, H6-2, C1, C2, C3, C4, C5, C6) are extracted. For molecular descriptors, we use the software, PaDEL-Descriptor^9^, to generate molecular descriptors (features). Only 1D and 2D features are utilized in this research. The total feature number of molecular descriptors is 1444.

### Feature encoding and data transformation

For basic properties, we use their original and binarized values as features. For example, for the sugar class Hex, it can be binarized into [Hex: 1, HexNAc: 0] (Supplementary Figure 6). For calculated chemical shifts, we use normalized and binarized values as features. For molecular descriptors, we use their original values as features. All feature values are rescaled into [0, 1] by Weka. Since the RRV distribution is an exponential distribution (x-axis: RRV, y-axis: frequency), we transform the regression target Y from RRV to ln (RRV), whose distribution is similar to the normal distribution (Supplementary Figure 7).

### Feature selection

We use the wrapper approach for evaluation in Weka^29^. Support vector regression is selected from the wrapper, and the backward greedy stepwise method is employed in the feature selection algorithm. In the first iteration, *N* features are used (e.g., *N*, the total feature number, is 1595). We used 5-fold cross-validation and PCC as parameters to evaluate the performance. In the second iteration, each feature will be eliminated from the feature set once. If a feature set (feature number equals to *N* − 1) can increase the PCC most or decrease the PCC least, this feature set will be used in the next iteration. When the feature number achieves 222, the optimized PCC is reached. In order to keep the complete sugar-type information, we use the selected 225 features for the final RRV prediction.

### Support vector machine for regression

Support vector machine is a machine learning approach proposed by Vapnik^30^ based on the structural risk minimization principle of statistical learning theory. It can be used to solve the classification or regression problem. Predicting the RRV of a BBL can be regarded as a regression problem. The program Weka Developed by the research group at the University of Waikato, is a powerful and well-known machine learning package used by many researchers. We apply the Weka (version 3.8) SVM regression model (SMOreg module) for predicting the RRV of each virtual BBLs.

### Virtual BBLs

There are two sugar classes, Hex and HexNAc, in our dataset. The Hex class includes galactoses, glucoses and mannoses and the HexNAc class includes *N*-acetylgalactosamines and *N*-acetylglucosamines. We generated the chemical structures of virtual BBLs for these sugar types by enumerating different substituents (Supplementary Figure 8). Four positions of each virtual BBL can be hydroxyl or protected. The positions R_2_, R_3_, R_4_, and R_6_ of a Hex virtual BBL could be OH, OAc, OBn, OBz, OClAc, OLev, NO2Bz, OPMB, OTBDPS, OTBS, or OTIPS. The total number of virtual Hex BBLs is 43,923 (=3 × 11^4^). The position R_2_ of a HexNAc virtual BBL could be NHTroc, NPhth, or N_3_ and R_3_, R_4_, and R_6_ could be OH, OAc, OBn, OBz, OClAc, OLev, NO_2_Bz, OPMB, OTBDPS, OTBS, or OTIPS. The total number of virtual HexNAc BBLs is 7986 (=2 × 3 × 11^3^). We used our optimized RRV model to predict the RRVs of these virtual BBLs. More than 50,000 virtual BBLs with predicted RRVs are added into the library, and they can be selected and used in the search procedure. To dispel the doubt about BBL structure similarity, we use Open Babel^31^ (by default setting) to calculate the Tanimoto similarity^32^ matrix (~50,000 × 117) between BBLs in the virtual BBL library and the training set. The matrix shows (Supplementary Data 14) that 99.45% of virtual BBLs are similar (Tanimoto similarity ≥ 0.75) with at least one BBL in the training set. 91.93% and 66.55% of virtual BBLs are similar (Tanimoto similarity ≥ 0.80 and ≥ 0.85) with at least one BBL in the training set, respectively. This result shows that most virtual BBLs are similar to BBLs in the training set. Dissimilar virtual BBLs in the library are rare and we do not need to worry about the ability of the RRV predictor for the novel (dissimilar) BBLs. We plan to add more BBLs to the library in the future as new BBLs may be prepared and the numbers of sugar type and sugar class may be expanded.

### General procedure for the determination of RRV

The general procedure for the determination of RRV can be found in our previous publication^4^. Detailed spectroscopic and analytical data for new compounds can be found in Supplementary Notes 5-6 and Supplementary Figures 9-38.

### Code availability

The Auto-CHO source code can be accessed from https://github.com/CW-Wayne/Auto-CHO.

## Electronic supplementary material

Supplementary Information

Peer Review File

Description of Additional Supplementary Files

Supplementary Data 1

Supplementary Data 2

Supplementary Data 3

Supplementary Data 4

Supplementary Data 5

Supplementary Data 6

Supplementary Data 7

Supplementary Data 8

Supplementary Data 9

Supplementary Data 10

Supplementary Data 11

Supplementary Data 12

Supplementary Data 13

Supplementary Data 14

## Data Availability

The Auto-CHO software, optimized Weka RRV predictor, and machine learning feature profiles can be accessed from https://sites.google.com/view/auto-cho/home. Requests for other materials should be addressed to corresponding authors.

## References

[1] Apweiler R, Hermjakob H, Sharon N (1999). On the frequency of protein glycosylation, as deduced from analysis of the SWISS-PROT database. Biochim. Biophys. Acta.

[2] Sears P, Wong CH (2001). Toward automated synthesis of oligosaccharides and glycoproteins. Science.

[3] Krasnova L, Wong CH (2016). Understanding the chemistry and biology of glycosylation with glycan synthesis. Annu. Rev. Biochem..

[4] Zhang Z (1999). Programmable one-pot oligosaccharide synthesis. J. Am. Chem. Soc..

[5] Douglas NL, Ley SV, Lücking U, Warriner SL (1998). Tuning glycoside reactivity: new tool for efficient oligosaccharide synthesis. J. Chem. Soc. Perkin Trans..

[6] ChemDraw (PerkinElmer Informatics).

[7] Cheeseman, J. R. & Frisch, Æ. *Predicting Magnetic Properties with Chemdraw and Gaussian* (Gaussian, Inc, Wallingford, 2000).

[8] Todeschini, R. & Consonni, V. *Handbook of Molecular Descriptors*. Vol. 11 (John Wiley & Sons, New York, 2008).

[9] Yap CW (2011). PaDEL‐descriptor: An open source software to calculate molecular descriptors and fingerprints. J. Comput. Chem..

[10] Canevari S, Fossati G, Balsari A, Sonnino S, Colnaghi MI (1983). Immunochemical analysis of the determinant recognized by a monoclonal antibody (MBr1) which specifically binds to human mammary epithelial cells. Cancer Res..

[11] Huang YL (2013). Carbohydrate-based vaccines with a glycolipid adjuvant for breast cancer. Proc. Natl Acad. Sci. USA.

[12] Lou YW (2014). Stage-specific embryonic antigen-4 as a potential therapeutic target in glioblastoma multiforme and other cancers. Proc. Natl Acad. Sci. USA.

[13] Zhang S (1997). Selection of tumor antigens as targets for immune attack using immunohistochemistry: I. Focus on gangliosides. Int. J. Cancer.

[14] Danishefsky SJ, Shue YK, Chang MN, Wong CH (2015). Development of Globo-H cancer vaccine. Acc. Chem. Res..

[15] Gilewski T (2001). Immunization of metastatic breast cancer patients with a fully synthetic globo H conjugate: a phase I trial. Proc. Natl Acad. Sci. USA.

[16] Huang CS (2016). Randomized phase II/III trial of active immunotherapy with OPT-822/OPT-821 in patients with metastatic breast cancer. J. Clin. Oncol..

[17] Burkhart F, Zhang Z, Wacowich‐Sgarbi S, Wong CH (2001). Synthesis of the globo H hexasaccharide using the programmable reactivity‐based one‐pot strategy. Angew. Chem. Int. Ed..

[18] Wang CC (2008). Glycan microarray of Globo H and related structures for quantitative analysis of breast cancer. Proc. Natl Acad. Sci. USA.

[19] Hsu CH (2010). Highly alpha‐selective sialyl phosphate donors for efficient preparation of natural sialosides. Chem. Eur. J..

[20] Polat T, Wong CH (2007). Anomeric reactivity-based one-pot synthesis of heparin-like oligosaccharides. J. Am. Chem. Soc..

[21] Dey S, Wong CH (2018). Programmable one-pot synthesis of heparin pentasaccharides enabling access to regiodefined sulfate derivatives. Chem. Sci..

[22] Shivatare SS (2016). Modular synthesis of N-glycans and arrays for the hetero-ligand binding analysis of HIV antibodies. Nat. Chem..

[23] Plante OJ, Palmacci ER, Seeberger PH (2001). Automated solid-phase synthesis of oligosaccharides. Science.

[24] Wang Z (2013). A general strategy for the chemoenzymatic synthesis of asymmetrically branched N-glycans. Science.

[25] Li L (2015). Efficient chemoenzymatic synthesis of an N-glycan isomer library. Chem. Sci..

[26] Damerell, D. et al. The GlycanBuilder and GlycoWorkbench glycoinformatics tools: updates and new developments. *Biol. Chem.* **393**, 1357–1362 (2012). 10.1515/hsz-2012-0135 23109548

[27] Ceroni A, Dell A, Haslam SM (2007). The GlycanBuilder: a fast, intuitive and flexible software tool for building and displaying glycan structures. Source Code Biol. Med..

[28] Wiederschain GY (2009). Essentials of glycobiology. Biochemistry.

[29] Witten, I. H., Frank, E., Hall, M. A. & Pal, C. J. *Data Mining: Practical Machine Learning Tools and Techniques* (Morgan Kaufmann, San Francisco, 2016).

[30] Vapnik, V. *The Nature of Statistical Learning Theory* (Springer Science & Business Media, New York, 2013).

[31] Open Babel: The Open Source Chemistry Toolbox. http://openbabel.org/wiki/Main_Page. (2016).

[32] Tanimoto, T. T. *An Elementary Mathematical Theory of Classification and Prediction* (International Business Machines Corporation, 1958).

