# Supplementary material for: Hierarchical and programmable one-pot synthesis of oligosaccharides
Source: Nat Commun. 2018 Dec 6;9:5202. doi: 10.1038/s41467-018-07618-8 (PMC6283847; doi:10.1038/s41467-018-07618-8)
Supplement: Supplementary file 1 — Supplementary Information [file 41467_2018_7618_MOESM1_ESM.pdf]

Supplementary Information

Hierarchical and Programmable One-pot Synthesis of Oligosaccharides

Cheng-Wei Cheng[1,2,3], Yixuan Zhou[4], Wen-Harn Pan[5], Supriya Dey[4], Chung-Yi Wu[4], Wen-Lian Hsu[2]*, and Chi-Huey Wong[4,6]*

[1]Bioinformatics Program, Taiwan International Graduate Program, Academia Sinica, Taipei 11529, Taiwan.
[2]Institute of Information Science, Academia Sinica, Taipei 11529, Taiwan.
[3]Institute of Biomedical Informatics, National Yang-Ming University, Taipei 11221, Taiwan.
[4]Genomics Research Center, Academia Sinica, Taipei 11529, Taiwan.
[5]Institute of Biomedical Sciences, Academia Sinica, Taipei 11529, Taiwan.
[6]Department of Chemistry, The Scripps Research Institute, La Jolla, CA 92037, USA.
*Corresponding Authors.

Supplementary Note 1 | Auto-CHO software manipulation

The program manipulation starts from the control panel of "(1) Query Glycan Structure" in Supplementary Figure 1. When a user clicks on the "Edit Glycan by GlycanBuilder" button, the program pops up the GlycanBuilder diagram that allows the user to edit the query glycan by Consortium for Functional Glycomics (CFG) notation or other notations provided by the GlycanBuilder. Note that any missing or ambiguous anomeric linkage is not acceptable by the software. After completing the editing and closing the GlycanBuilder diagram, the query structure on the panel changes to the latest one.

As the user clicks on the "Search Building Block Library" button, the program takes the query glycan structure to search the building block library. Then, it returns with all possible synthetic solutions at the "(2) Search Results" region in Supplementary Figure 1. The reducing end acceptors of different residue numbers are displayed in the reducing end acceptor column. When a reducing end acceptor is selected, solutions are displayed on the "Synthetic Solution List." Also, fragments are shown in the "Fragment List" to suggest how many fragments should be used in the synthesis. The system gives detailed information of each fragment, including the RRV of the fragment, computational yield, and protecting groups on the fragment as well as which protecting group should be deprotected for the subsequent use of the fragment in the one-pot reaction. The building blocks used to assemble the selected fragment are shown in the bottom. The software automatically displays chemical structures and detailed information of the selected building blocks in the regions of "(3) Chemical Structure of Building Block" and "(4) Building Block Browser", respectively.

Users can also browse other building block information on the "(4) Building Block Browser" region. The chemical structure of the selected building block is then shown on the "(3) Chemical Structure of Building Block" panel. The IUPAC name of the selected building block is shown below the chemical structure. Users can hide this description through adjusting the setting function. Details of the selected building block, including RRV, acceptor position, product anomer, and sugar type are shown in the bottom-right region of the screen. This section is for the Auto-CHO version 1.0 and could be updated in the future. Please check our website for the latest description.

Supplementary Note 2 | Comparison between experimental and calculated ^{1}H-NMR chemical shifts

It is difficult to get all experimental NMR chemical shifts of building blocks with each atom assignment. Here, we collect 16 building blocks (12 galactoses and 4 mannoses) that have complete ^{1}H-NMR chemical shift assignments for each atom in the training set. For each building block, we have 7 values of experimental and calculated chemical shifts, respectively. Then we compare the experimental and calculated ^{1}H-NMR chemical shifts of these building blocks and it shows that there is no significant difference between the experimental and calculated ones by t-test and F-test. The chemical shift profile can be found in Supplementary Data 9.

- Data
  - 16 Hex building blocks
    - 12 galactoses (Product Anomer: 4 alpha forms, 8 beta forms)
    - 4 mannoses (Product Anomer: 4 alpha forms, no beta forms)
  - RRV range: 1 ~ 52000
- Experimental ^{1}H-NMR chemical shifts
  - Average = 4.45
  - Standard deviation = 0.680069
- Calculated ^{1}H-NMR chemical shifts by ChemDraw
  - Average = 4.46
  - Standard deviation = 0.79028
- T-test (α = 0.05)
  - P-value = 0.935713 (two-tailed)
  - P-value = 0.844348 (two-tailed, paired t-test)
- F-test (α = 0.025)
  - P-value = 0.060855 (one-tailed)
- PCC = 0.839252

Supplementary Note 3 | Cross-validation

Leave-one-out cross-validation (LOOCV) is a commonly used approach for validating the performance of a model. We can apply it to optimize the parameters of the trained model. For each particular parameter setting, N-1 (e.g. N = 117) instances are used for training, and the remaining one is used for testing. This procedure repeats N times, and each instance in the data set is used for testing. PCC and MAE have been used for evaluating the performance of SVM regression models, and the optimized model is used for RRV prediction.

Since LOOCV is time-consuming, we also adopt 10-fold cross-validation to evaluate feature selection performance. In each iteration, 9-folds are trained for feature selection and one-fold is tested with selected features from training part (9-folds). In other words, this evaluation avoids seeing data from test part in feature selection. The same feature selection strategy mentioned above is used here.

Supplementary Note 4 | Performance evaluation

Pearson's Correlation Coefficient (PCC) can help us measure the correlation between the observed and predicted RRVs. PCC is a value between +1 and -1, where +1 means total positive linear correlation, 0 means no correlation, and -1 means total negative linear correlation. Supplementary Equation 1 shows the formula.

$$\mathrm{PCC} = r_{xy} = (n\sum x_i y_i - \sum x_i \sum y_i)/\sqrt{n\sum x_i^2 - (\sum x_i)^2}\sqrt{n\sum y_i^2 - (\sum y_i)^2} \quad (1)$$

where x_i is a predicted value and y_i is an observed value.

Mean Absolute Error (MAE) is an average error measurement for the predicted and observed values. The smaller the MAE is; the better accuracy is achieved. Supplementary Equation 2 shows the definition.

$$\mathrm{MAE} = \frac{1}{n}\sum_{i=1}^{n} |\hat{\theta}_i - \theta_i| \quad (2)$$

where $\hat{\theta}_i$ is a predicted value and θ_i is an observed value.

Relative Absolute Error (RAE) is an error measurement for the predicted and observed values. The difference between RAE and MAE is that RAE is a value between 0 and 1, where 0 means the perfect prediction. Supplementary Equation 3 is the RAE definition.

$$\mathrm{RAE} = \frac{\sum_{i=1}^{n} |\hat{\theta}_i - \theta_i|}{\sum_{i=1}^{n} |\bar{\theta} - \theta_i|} \quad (3)$$

where $\hat{\theta}_i$ is a predicted value, θ_i is an observed value, and $\bar{\theta} = \frac{1}{n}\sum_{i=1}^{n} \theta_i$.

Since the one-pot glycan synthesis usually uses the most reactive, less reactive and least reactive BBLs sequentially, the RRV values can be classified into three categories which are [>15,000], [1000, 15,000], and [0, 1000] according to the rule of thumb. Accuracy (ACC) is used for evaluating the RRV category prediction. Supplementary Equation 4 shows the ACC formula.

$$ACC = \frac{TP+TN}{TP+FP+FN+TN} \quad (4)$$

where TP, TN, FP, FN represent true positive, true negative, false positive, and false negative, respectively.

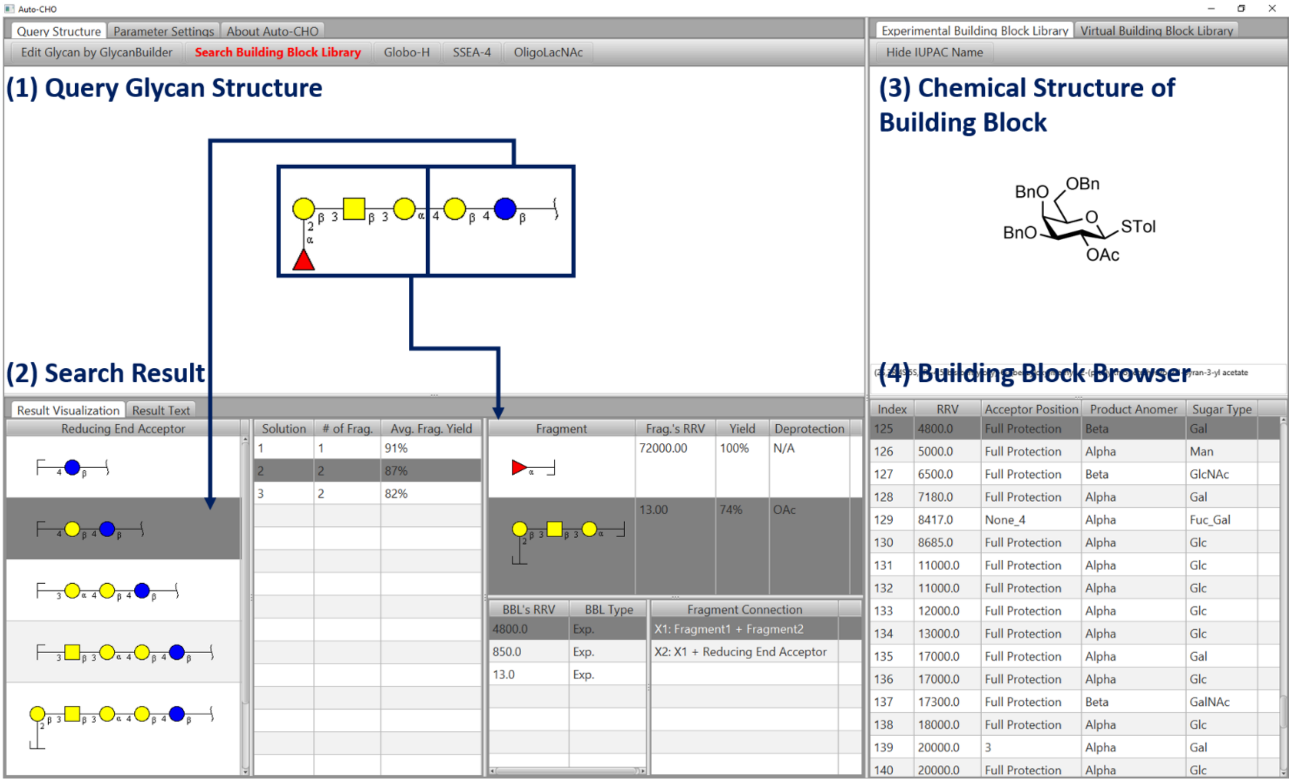

Supplementary Figure 1 | User interface of Auto-CHO software (version 1.0) showing the synthesis of Globo-H. The interface can be divided into four parts, including (1) Query of Glycan Structure, (2) Search Result, (3) Structure of Building Block, and (4) Building Block Browser.

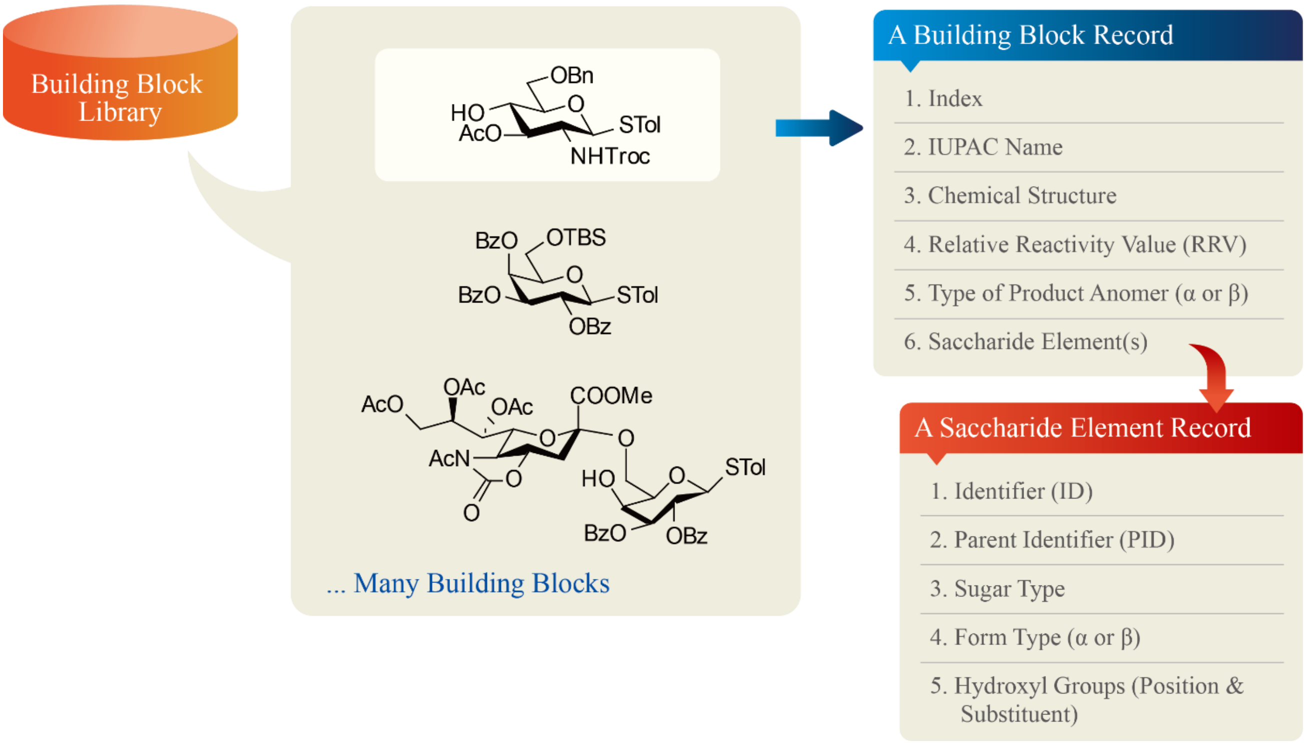

Supplementary Figure 2 | Records of experimentally validated building blocks in the library.

a

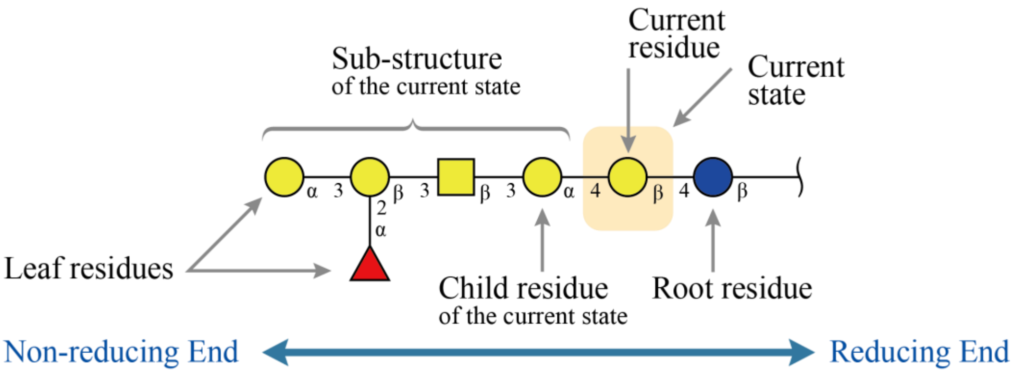

b

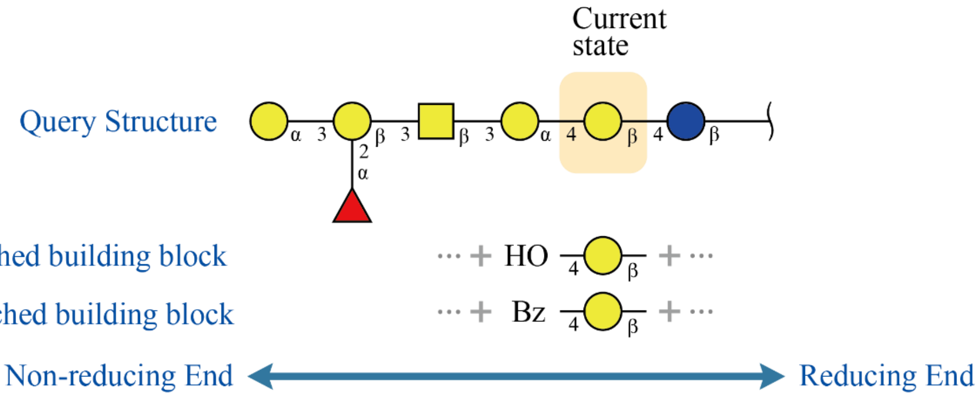

Supplementary Figure 3 | Illustration for the search algorithm. a, Glycan data structure description in the algorithm. **b**, Perfectly matched building block and precursor matched building block.

a

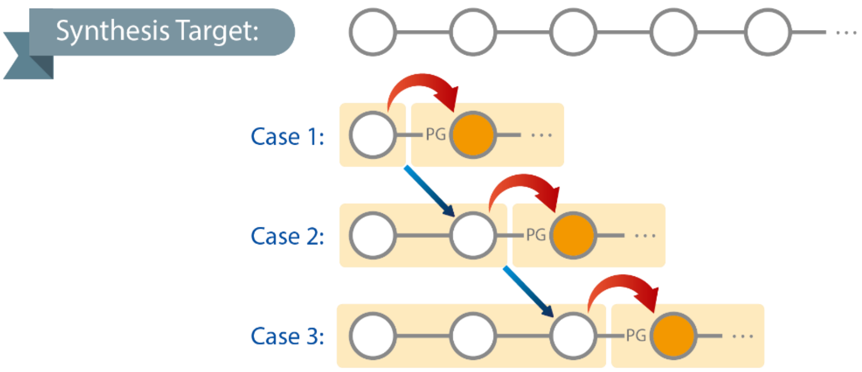

b

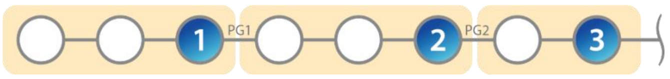

c

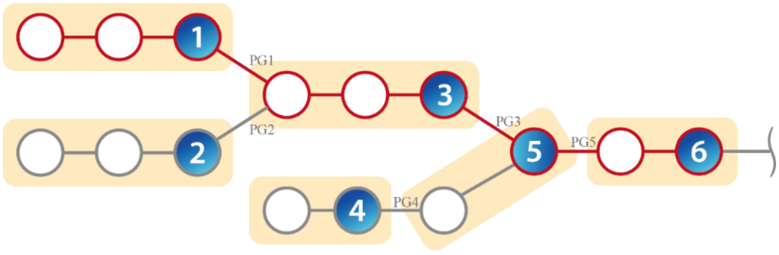

Supplementary Figure 4 | Illustration for fragment elongation and connection. a, Three cases of fragment elongation. The orange circles represent precursor matched building block. The orange boxes represent fragments. PG means protecting group which can be deprotected before fragment connection. **b,** Illustration of a linear saccharide target synthesized by three fragments. **c,** This tree-shaped saccharide target can be synthesized by six fragments.

Building Block #40 (Man, RRV=123)

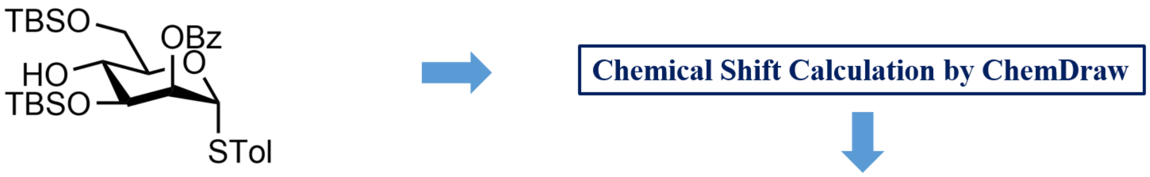

^{1}H-NMR Chemical Shift

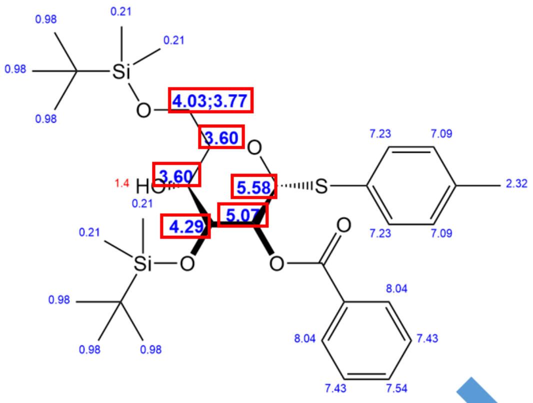

^{13}C-NMR Chemical

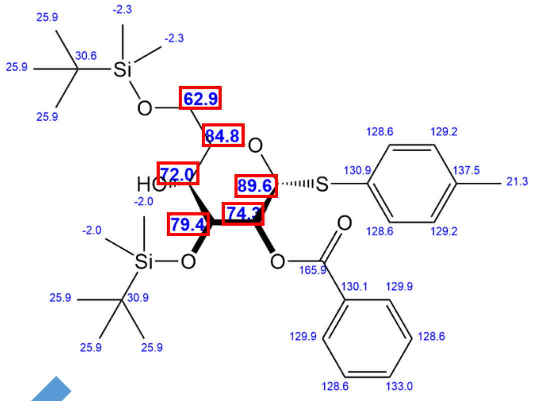

Feature vector

Chemical Shift	H1	H2	H3	H4	H5	H6_1	H6_2	C1	C2	C3	C4	C5	C6
Value	5.58	5.07	4.29	3.60	3.60	4.03	3.77	89.6	74.3	79.4	72.0	84.8	62.9

Supplementary Figure 5 | An example of feature extraction from the chemical structure of a building block.

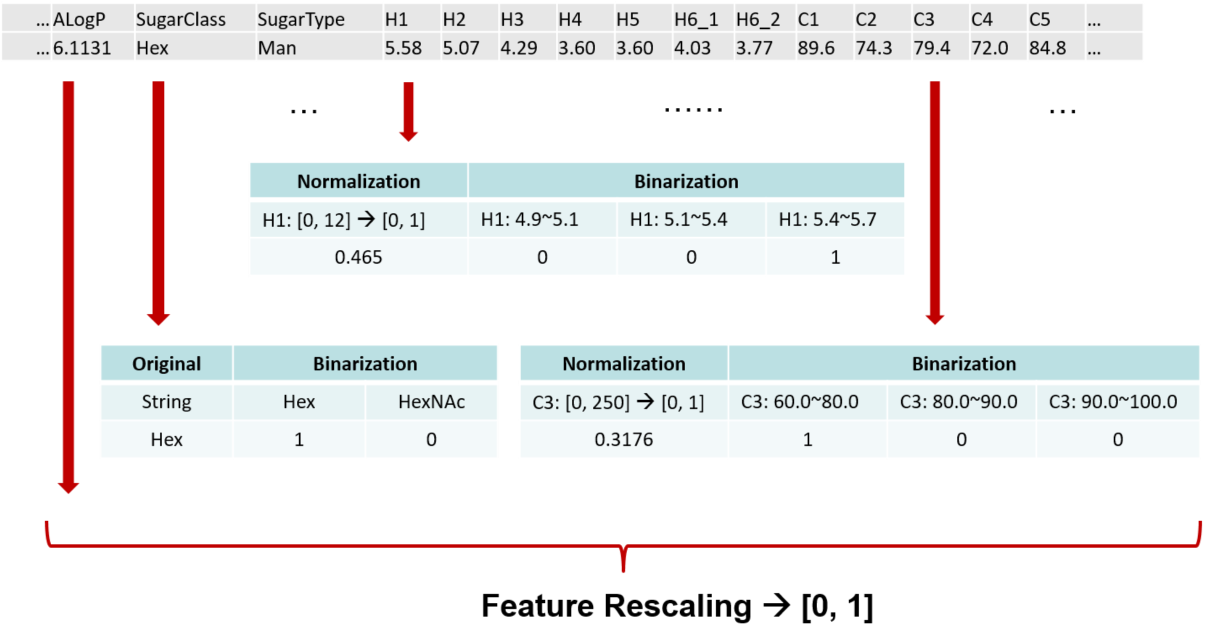

Supplementary Figure 6 | An illustration of a feature vector encoding.

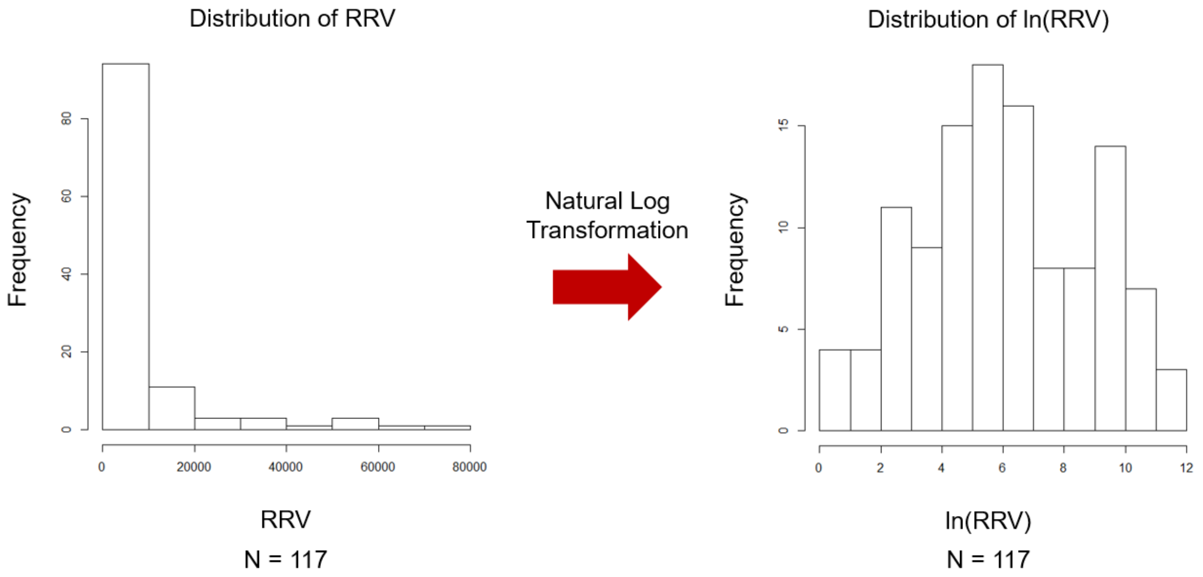

Supplementary Figure 7 | The distributions of RRV and ln(RRV). The ln(RRV) distribution is similar to a normal distribution.

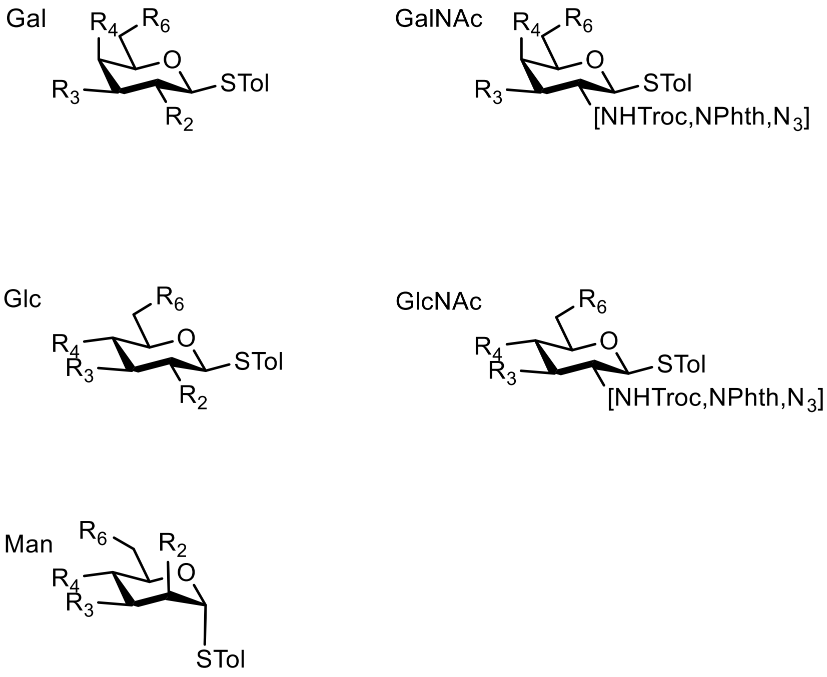

Supplementary Figure 8 | Chemical structures of virtual building blocks.

Supplementary Table 1 | RRV prediction performance and data set summary. a, The performance with different number of selected features by feature selection with 10-fold cross-validation. **b,** RRV prediction performance according to RRV class (LOOCV). **c,** Training set summary for the final RRV predictor.

a

# of Selected Features	PCC	MAE	RAE	Accuracy
**222**	0.8906	2558.37	0.2684	86.32%
**100**	0.8976	2552.62	0.2678	84.62%
**75**	0.8871	2780.27	0.2917	84.62%
**50**	0.8898	2910.36	0.3053	81.20%
**25**	0.8856	2798.14	0.2935	78.63%

b

Class	PCC	MAE	Accuracy	# of Instance
**All range**	0.9701	1,253.31	97.44%	117
**[>15,000]**	0.8863	6,191.78	94.74%	19
**[1,000~15,000]**	0.9436	1,156.93	95.24%	21
**[0~1,000]**	0.8886	61.01	98.70%	77

c

Sugar Type	Gal	Glc	Man	GalNAc	GlcNAc	Total
**# of building block**	26	36	13	13	29	117

Supplementary Note 5 | Compound information of building blocks for RRV independent test

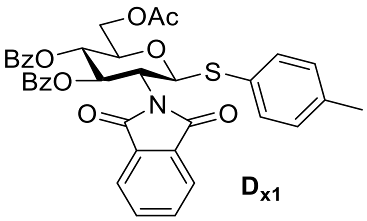

D_{x1}

^{1}H NMR (600 MHz, $CDCl_3$): δ 7.87-7.85 (m, 3H), 7.72-7.65 (m, 5H), 7.47-7.44 (m, 1H), 7.39-7.36 (m, 1H), 7.34-7.30 (m, 4H), 7.23-7.21(m, 2H), 7.08 (d, J = 7.8 Hz, 2H), 6.22 (dd, J_1= 10.2, J_2= 9.6 Hz, 1H), 5.80 (d, J = 10.2 Hz, 1H), 5.55 (dd, J $_1$= 9.6, J_2= 9.6 Hz, 1H), 4.55 (dd, J_1 = 10.2, J_2= 9.6 Hz, 1H), 4.31-4.30 (m, 2H), 4.12 (m, 1H), 3.71 (dd, J_1= 12.0, J_2= 3.6 Hz, 1H), 2.49 (br, 1H), 2.31 (s, 3H); ^{13}C NMR (150 MHz, $CDCl_3$): δ 170.5, 168.0, 166.9, 165.6, 165.1, 138.7, 134.3, 134.2, 133.8, 133.4, 133.2, 131.6, 131.1, 129.7, 129.7, 129.6, 128.7, 128.4, 128.4, 128.2, 127.1, 123.6, 83.4, 76.0, 72.0, 69.6, 62.6, 53.8, 21.2, 20.7; HRMS (m/z): $[M+Na]^+$ calcd. for $C_{37}H_{31}NO_9SNa$, 688.1612; Found, 688.1645.

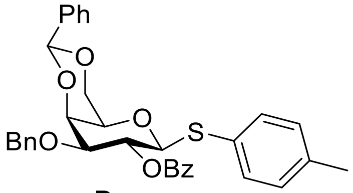

D_{x2}

Please see the reference[1].

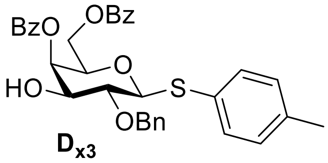

D_{x3}

^{1}H NMR (600 MHz, $CDCl_3$): δ 8.07-8.04 (m, 4H), 7.65-7.59 (m, 2H), 7.57-7.55 (m, 2H), 7.50-7.42 (m, 6H), 7.37-7.31 (m, 3H), 7.07 (d, J = 7.98 Hz, 2H), 5.71 (d, J = 3.24 Hz, 1H), 5.01 (d, J = 10.5 Hz, 1H), 4.74-4.70 (m, 2H), 4.58 (dd, J_1 = 11.6 Hz, J_2 = 7.3 Hz, 1H), 4.43 (dd, J_1 = 11.6 Hz, J_2 = 5.5 Hz, 1H), 4.09 (m, 1H), 3.99 (dd, J_1 = 9.1 Hz, J_2 = 3.4 Hz, 1H), 3.72 (t, J_1 = 9.3 Hz, J_2 = 9.4 Hz, 1H), 2.36 (s, 3H). ^{13}C NMR (150 MHz, $CDCl_3$): δ 166.3, 166.1, 137.9, 137.9, 133.5, 133.2, 133.0, 130.1, 129.8, 129.7, 129.6, 129.3, 128.6, 128.5, 128.4, 128.3, 128.1, 87.6, 77.8, 75.5, 75.0, 74.0, 70.5, 62.9, 21.2; HRMS (m/z): $[M+H]^+$ calcd. for $C_{34}H_{33}O_7S$, 585.1942; found, 585.1944.

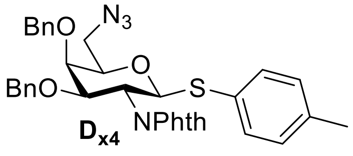

D_{x4}

^{1}H NMR (600 MHz, $CDCl_3$): δ 7.89 (m, 2H), 7.73 (m, 3H), 7.36-7.24 (m, 7H), 7.07-6.98 (m, 7H), 5.46 (d, J = 10.5 Hz, 1H), 5.02 (d, J = 11.8 Hz, 1H), 4.79 (t, J_1 = 11.2 Hz, J_2 = 10.5 Hz, 1H), 4.60 (m, 2H), 4.33 (m, 2H), 3.90 (d, J = 2.6 Hz, 1H), 3.63 (m, 2H), 3.18 (m, 1H), 2.26 (s, 1H). ^{13}C NMR (150 MHz, $CDCl_3$): δ 168.4, 167.5, 138.1, 138.0, 137.3, 134.3, 133.1, 131.7, 129.6, 128.6, 128.4, 128.3, 128.3, 127.8, 127.8, 127.7, 123.6, 84.8, 77.6, 77.4, 77.3, 77.1, 76.9, 74.4, 72.2, 72.8, 51.5, 51.4, 21.1; HRMS (m/z): $[M+Na]^+$ calcd. for $C_{35}H_{32}N_4O_5SNa$, 643.1986; found, 643.1990.

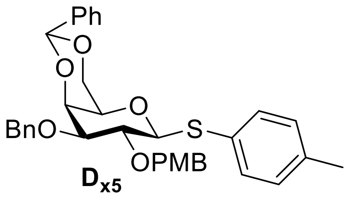

D$_{x5}$

^{1}H NMR (600 MHz, $CDCl_3$): δ 7.60-7.59 (m, 2H), 7.52-7.50 (m, 2H), 7.39-7.33 (m, 7H), 7.30-7.26 (m, 3H), 7.00 (d, J = 8.1 Hz, 2H), 6.87-6.86 (m, 2H), 5.46 (s, 1H), 4.70 (dd, J_1 = 12.2 Hz, J_2 = 5.5 Hz, 1H), 4.63 (dd, J_1 = 9.9 Hz, J_2 = 2.5 Hz, 1H), 4.53 (d, J = 9.5 Hz, 1H), 4.34 (dd, J_1 = 12.2 Hz, J_2 = 1.5 Hz, 1H), 4.11 (d, J = 3.0 Hz, 1H), 3.95 (dd, J_1 = 12.2 Hz, J_2 = 1.5 Hz, 1H), 3.82 (t, J_1 = 9.3 Hz, J_2 = 9.3 Hz, 1H), 3.79 (s, 3H), 3.59 (dd, J_1 = 9.3 Hz, J_2 = 3.4 Hz, 1H), 3.37 (s, 1H), 2.29 (s, 3H). ^{13}C NMR (150 MHz, $CDCl_3$): δ 159.5, 138.4, 138.1, 137.8, 133.6, 131.0, 130.0, 129.8, 129.2, 129.0, 128.6, 128.3, 128.0, 127.9, 126.9, 114.0, 101.5, 86.9, 81.6, 75.4, 75.3, 73.9, 72.0, 69.9, 69.6, 55.5, 21.3; HRMS (m/z): $[M+Na]^+$ calcd. for $C_{35}H_{36}O_6SNa$, 607.2125; found, 607.2149.

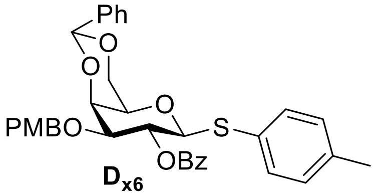

D$_{x6}$

^{1}H NMR (600 MHz, $CDCl_3$): δ 8.02-8.00 (m, 2H), 7.59-7.56 (m, 1H), 7.47-7.41 (m, 6H), 7.34-7.32 (m, 3H), 7.06 (m, 2H), 7.02 (d, J = 7.86 Hz, 2H), 6.64 (m, 2H), 5.46 (m, 2H), 4.73 (d, J = 9.78 Hz, 1H), 4.53 (d, J = 12.5 Hz, 1H), 4.45 (d, J = 12.5 Hz, 1H), 4.35 (dd, J_1 = 12.2 Hz, J_2 = 1.4 Hz, 1H), 4.18 (d, J = 3.0 Hz, 1H), 4.00 (dd, J_1 = 12.2 Hz, J_2 = 1.4 Hz, 1H), 3.71 (m, 4H), 3.45 (d, J = 0.8 Hz, 1H), 2.30 (s, 3H). ^{13}C NMR (150 MHz, $CDCl_3$): δ 165.1, 159.3, 138.3, 137.9, 134.6, 133.1, 130.5, 130.3, 130.0, 129.9, 129.6, 129.5, 129.2, 128.6, 128.5, 128.2, 127.8, 126.9, 113.8, 101.5, 85.7, 77.9, 73.4, 70.8, 70.2, 69.5, 69.3, 55.3, 21.4; HRMS (m/z): $[M+Na]^+$ calcd. for $C_{35}H_{34}O_7SNa$, 621.1917; found, 621.1911.

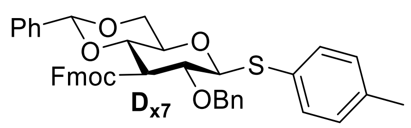

^{1}H NMR (600 MHz, $CDCl_3$): δ 7.74-7.72 (m, 2H), 7.56-7.51 (m, 2H), 7.44-7.43 (m, 2H), 7.41-7.39 (m, 2H), 7.34 (m, 2H), 7.30-7.26 (m, 5H), 7.25-7.13 (m, 8H), 5.50 (s, 1H), 5.21 (t, J_1 = 9.1 Hz, J_2 = 9.5 Hz, 1H), 4.91 (d, J = 10.7 Hz, 1H), 4.75 (d, J = 9.7 Hz, 1H), 4.64 (d, J = 10.7 Hz, 1H), 4.42 (dd, J_1 = 10.6 Hz, J_2 = 7.2 Hz, 1H), 4.37 (dd, J_1 = 10.5 Hz, J_2 = 5.0 Hz, 1H), 4.27 (dd, J_1 = 10.4 Hz, J_2 = 7.5 Hz, 1H), 4.17 (t, J_1 = 7.3 Hz, J_2 = 7.3 Hz, 1H), 3.79 (t, J_1 = 10.3 Hz, J_2 = 10.3 Hz, 1H), 3.68 (t, J_1 = 9.6 Hz, J_2 = 9.7 Hz, 1H), 3.59 (t, J_1 = 9.4 Hz, J_2 = 9.1 Hz, 1H), 3.51 (m, 1H), 2.35 (s, 3 H). ^{13}C NMR (150 MHz, $CDCl_3$): δ 154.6, 143.6, 143.4, 141.4, 138.7, 137.7, 137.0, 133.3, 130.1, 129.2, 129.0, 128.5, 128.4, 128.3, 128.1, 128.0, 127.4, 127.3, 126.4, 125.3, 125.2, 120.2, 101.6, 88.7, 79.4, 79.2, 78.6, 75.6, 70.4, 70.4, 68.8, 46.8, 21.4; HRMS (m/z): $[M+Na]^+$ calcd. for $C_{42}H_{38}O_7SNa$, 709.2230; found, 709.2233.

Supplementary Note 6 | Compound information for the Figure 3

a

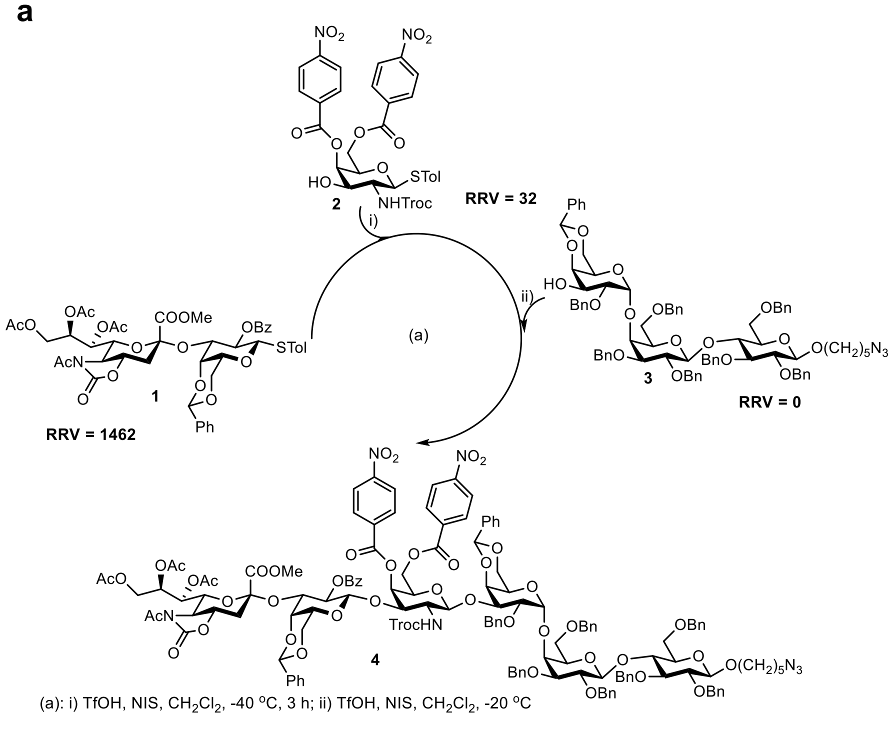

Compound 1 and All Sialylated Disaccharides in Figure 3
Please see reference[2].

Compound 2
^{1}H NMR (600 MHz, $CDCl_3$): δ 8.30 (d, J = 9.5 Hz, 2H), 8.17 (m, 2H), 8.00 (m, 4H), 7.55 (d, J = 8.0 Hz, 2H), 7.23 (d, J = 8.0 Hz, 2H), 5.83 (m, 1H), 5.56 (dd, J_1 = 10.8 Hz, J_2 = 2.7 Hz, 1H), 5.36 (d, J = 9.4 Hz, 1H), 4.93 (d, J = 10.1 Hz, 1H), 4.76 (d, J = 12.2 Hz, 1H), 4.65 (d, J = 12.2 Hz, 1H), 4.22 (m,

1H), 4.07 (m, 1H), 3.87 (m, 1H), 3.66 (m, 1H), 2.46 (s, 3H). ^{13}C NMR (150 MHz, $CDCl_3$): δ 164.2, 164.0, 154.1, 151.0, 139.3, 134.9, 133.9, 133.8, 131.0, 130.9, 129.9, 126.4, 123.8, 123.6, 95.3, 85.8, 74.4, 73.6, 69.0, 60.9, 51.3, 21.4; HRMS (m/z): $[M+H]^+$ calcd. for $C_{30}H_{26}Cl_3N_3O_{12}S$, 757.0303; found, 758.0394.

Compound 3

Please see reference[3].

Compound 4

A solution of disaccharide **1** donor (38 mg, 1.1 eq., 0.057 mmol), the first acceptor **2** (40 mg, 1.0 eq., 0.053 mmol) and pulverized activated 4 Å MS (3 $gmmol^{-1}$) in dry DCM (1.0 mL) was stirred under argon at room temperature for 2 h. The mixture was then cooled to -40°C followed by addition of NIS (13 mg, 1.1 eq., 0.057 mmol) and TfOH (34 µL, 0.3 eq., 0.017 mmol, 0.5 M in ether). After 3 h when TLC indicated that the first acceptor **2** is almost consumed, the acceptor **3** (80 mg, 1.2 eq., 0.062 mmol), NIS (19 mg, 1.6 eq., 0.083 mmol), TfOH (34 µL, 0.3 eq., 0.017 mmol, 0.5 M in ether) were added. The reaction was kept stirred at -20°C for 3 h. The reaction was neutralized by saturated $NaHCO_3$ (aq.), diluted with DCM and filtered with a pad of Celite. The filtrate was poured into a mixture of saturated $NaHCO_3$ (aq.) and saturated $Na_2S_2O_3$ (aq.). The aqueous layer was extracted with two portion of DCM. The combined extracts were washed with brine, dried over Mg_2SO_4, filtered, and concentrated in vacuo. The residue was purified by silica gel column chromatogrghy (EtOAc/ Toluene 1 :4 to 1:2), to afford fully protected SSEA-4 derivative **4** (74 mg, 50 % based on acceptor **2**) as white foam. ^{1}H NMR (600 MHz, $CDCl_3$): δ 8.15-7.90 (m, 9H), 7.54-7.16 (m, 49H), 5.70-5.62 (m, 2H), 5.50 (s, 1H), 5.35-5.29 (m, 3H), 5.11 (s, 1H), 5.03-5.01 (m, 2H), 4.90-4.47 (m, 18H), 4.39-3.94 (m, 25H), 3.87-3.84 (m, 2H), 3.76-3.70 (m, 2H), 3.64-3.30 (m, 19H), 3.21 (t, J=6.9 Hz, 2H), 2.87 (dd, J=11.9, 2.7 Hz, 1H), 2.47 (s, 3H), 2.24 (s, 3H), 2.05-2.03 (m, 7H), 1.66-1.60 (m, 4H), 1.50-1.45 (m, 2H); ^{13}C NMR (150 MHz, $CDCl_3$): δ 172.1, 171.0, 170.5, 170.1, 168.6, 164.8, 164.2, 164.0, 153.9, 153.4, 150.6, 150.3, 139.5, 138.7, 138.4, 138.3, 138.0, 137.9, 137.6, 135.2, 134.8, 133.1, 131.0, 130.9, 130.2, 129.6, 129.1, 128.9, 128.6, 128.5, 128.4, 128.3, 128.2, 128.1, 128.0, 127.9, 127.8, 127.7, 127.6, 127.5, 126.5, 126.1, 123.6, 123.3, 103.5, 103.1, 101.9, 100.6, 100.5, 99.8, 96.6, 95.8, 82.1, 81.7, 81.2, 78.9, 75.5, 75.0, 74.9, 74.8, 74.7, 74.3, 74.1, 73.7, 73.1, 73.0, 72.9, 72.4, 72.1, 72.0, 71.3, 70.8, 70.1, 70.0, 69.6, 69.0, 68.6, 68.4, 67.8, 67.0, 66.3, 63.7, 63.0, 62.4, 58.8, 54.5, 52.8, 51.3, 37.3, 29.7, 29.3, 28.6, 24.7, 23.4, 22.7, 21.4, 20.9, 14.1; HRMS (m/z): $[M+Na]^+$ calcd. for $C_{141}H_{146}Cl_3N_7O_{46}Na$, 2803.8262; found, 2803.8119.

b

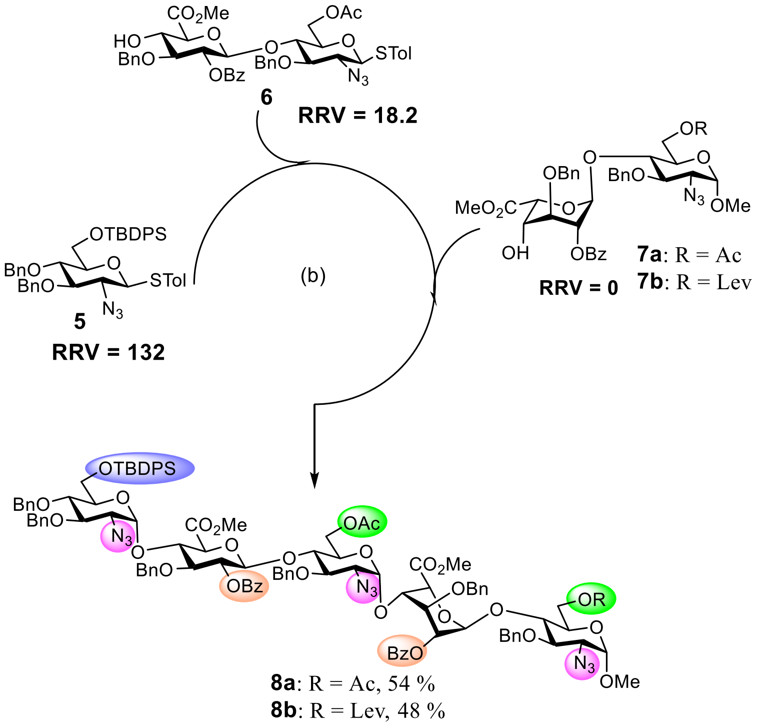

(b): NIS/TfOH, CH_2Cl_2, -45 °C to -25 °C

Compound 5

^{1}H NMR (600 MHz, $CDCl_3$): δ 7.80 (d, J = 7.2 Hz, 2H), 7.70 (d, J = 7.2 Hz, 2H), 7.54 (d, J = 7.8 Hz, 2H), 7.44-7.26 (m, 14H), 7.16 (app d, 2H), 7.05 (d, J = 7.8 Hz, 2H), 4.86 (app t, J = 6.0 Hz, 3H), 4.70 (d, J = 10.8 Hz, 1H), 4.38 (d, J = 10.2 Hz, 1H), 4.02 (d, J = 11.4 Hz, 1H), 3.94 (dd, J = 11.4, 3.0 Hz, 1H), 3.78 (t, J = 9.6 Hz, 1H), 3.54 (t, J = 9.6 Hz, 1H), 3.36 (app t, J = 9.6 Hz, 2H), 2.32 (s, 3H), 1.10 (s, 9H); ^{13}C NMR (150 MHz, $CDCl_3$): δ 138.0, 137.6, 135.9, 134.2, 133.4, 132.8, 129.8-127.7, 127.2, 86.1, 85.3, 80.0, 76.1, 75.1, 64.8, 62.3, 26.9, 21.2, 19.3; HRMS (m/z): $[M+Na]^+$ calcd. for $C_{43}H_{47}N_3O_4SSiNa$, 752.2949; found, 752.2984.

Compound 6

^{1}H NMR (600 MHz, $CDCl_3$): δ 8.12 (d, J = 7.8 Hz, 2H), 7.78 (t, J = 7.2 Hz, 1H), 7.58 (t, J = 7.8 Hz, 2H), 7.50-7.44 (m, 7H), 7.40-7.38 (m, 2H), 7.28-7.26 (m, 3H), 7.18 (d, J = 7.8 Hz, 2H), 5.40 (t, J = 8.4 Hz, 1H), 5.20 (d, J = 11.4 Hz, 1H), 4.88 (d, J = 12.0 Hz, 2H), 4.82 (d, J = 11.4 Hz, 1H), 4.77 (d, J = 7.8 Hz, 1H), 4.38-4.36 (m, 1H), 4.32 (d, J = 10.2 Hz, 1H), 4.22 (dd, J = 12.0, 4.8 Hz, 1H), 4.14 (t, J = 9.0 Hz, 1H), 3.90 (d, J = 9.6 Hz, 1H), 3.81-3.77 (m, 2H), 3.65 (s, 3H), 3.55 (t, J = 9.0 Hz, 1H), 3.40-3.34 (m, 2H), 2.44 (s, 3H), 2.05 (s, 3H); ^{13}C NMR (150 MHz, $CDCl_3$): δ 170.4, 169.3, 164.8, 138.9, 138.3, 137.7, 134.6, 133.5, 129.9-126.6, 101.4, 85.7, 83.0, 80.9, 77.6, 76.8, 75.7, 74.7, 74.4, 73.1, 72.3, 64.6, 62.1, 52.8, 21.2, 20.8; HRMS (m/z): $[M+Na]^+$ calcd. for $C_{43}H_{45}N_3O_{12}SNa$, 850.2622; found, 850.2627.

Compound 7a

^{1}H NMR (600 MHz, $CDCl_3$): δ 7.97 (m, 2H), 7.59 (t, J = 7.2 Hz, 1H), 7.44 (t, J = 7.8 Hz, 2H), 7.40-7.22 (m, 9H), 7.28-7.15 (m, 1H), 5.27 (br s, 1H), 5.16 (br s, 1H), 4.94 (d, J = 2.4 Hz, 1H), 4.81 (d, J = 4.8 Hz, 1H), 4.80-4.78 (m, 2H), 4.71-4.67 (m, 2H),4.43 (dd, J = 12.6, 1.8 Hz, 1H), 4.32 (dd, J = 12.0, 4.2 Hz, 1H), 4.00 (app dd, J = 10.2, 2.4 Hz, 1H), 3.94-3.92 (m, 1H), 3.90 (app t, J = 3.0 Hz, 1H), 3.86-3.83 (m, 2H), 3.53-3.51 (m, 1H), 3.50 (s, 3H), 3.43 (app s, 3H), 2.66 (app d , J = 10.8 Hz, 1H), 2.07 (s, 3H);^{13}C NMR (150 MHz, $CDCl_3$): δ 170.7, 169.7, 165.1, 137.9, 137.8, 137.5, 133.9, 129.9-125.4, 98.7, 98.2, 78.8, 75.4, 75.1, 74.7, 72.7, 69.0, 68.9, 68.1, 68.0, 63.8, 62.3, 55.5, 52.1, 20.9; HRMS (m/z): $[M+Na]^+$ calcd. for $C_{37}H_{41}N_3O_{13}Na$, 758.2537; found, 758.2548.

Compound 7b

^{1}H NMR (600 MHz, $CDCl_3$): δ 7.95-7.90 (m, 2H), 7.55-7.49 (m, 1H), 7.39-7.36 (m, 2H), 7.34-7.30 (m, 4H), 7.30-7.27 (m, 2H), 7.24 (dt, J = 7.5, 1.8 Hz, 1H), 7.23-7.20 (m, 2H), 7.18-7.14 (m, 1H), 5.18 (d, J = 1.5 Hz, 1H), 5.10 (ddd, J = 3.1, 2.0, 1.0 Hz, 1H), 4.94 (s, 1H), 4.87 (d, J = 2.3 Hz, 1H), 4.75 (d, J = 2.1 Hz, 1H), 4.73 (dd, J = 5.5, 3.2 Hz, 3H), 4.62 (dd, J = 11.1, 8.1 Hz, 2H), 4.36 (dd, J = 12.4, 2.2 Hz, 1H), 4.27 (dd, J = 12.4, 3.8 Hz, 1H), 3.97 (s, 1H), 3.88 (dd, J = 10.0, 8.9 Hz, 1H), 3.84 – 3.82 (m, 1H), 3.79 (dd, J = 3.9, 2.3 Hz, 1H), 3.79-3.76 (m, 1H), 3.44 (s, 3H), 3.38 (d, J = 3.6 Hz, 1H), 3.37 (s, 3H), 2.65 (td, J = 6.5, 2.8 Hz, 2H), 2.60-2.53 (m, 1H), 2.50 (dt, J = 17.3, 6.6 Hz, 1H), 2.02 (s, 3H);^{13}C NMR (150 MHz, $CDCl_3$): δ 206.5, 177.0, 172.4, 169.7, 165.1, 137.9, 137.3, 133.7, 129.9-127.4, 98.6, 98.0, 79.3, 78.7, 75.1, 75.0, 74.8, 72.6, 69.1, 68.9, 68.1, 68.0, 63.8, 62.5, 55.5, 52.1, 37.9, 29.7, 29.6, 27.6; HRMS (m/z): $[M+Na]^+$ calcd. for $C_{40}H_{45}N_3O_{14}Na$, 814.2799; found, 814.2792.

Compound 8a

Glucosyl donor **5** (0.042 g, 0.058 mmol), azidoglucosyl acceptor **6** (0.04 g, 0.048 mmol) and flame activated AW-300 MS (0.3 g) were suspended in dry CH_2Cl_2 (1 mL) for 1 h at room temperature under N_2 atmosphere. It was then cooled to to -45 °C. NIS (0.019 g, 0.087 mmol) and 1 M TfOH in CH_2Cl_2 (12 μL, 0.018 mmol) were added and the reaction was allowed to warm up to -30 °C. The disaccharide acceptor **7a** (0.035 g, 0.043 mmol) in dry CH_2Cl_2 (1 mL) and MS AW-300 (0.1 g) were stirred for 30 min at room temperature. After consumption of donor **5** and acceptor **6** (TLC, $PhCH_3$/EtOAc, 20:1), the mixture was again cooled to -45 °C and followed by addition of acceptor **7a**. NIS (0.017 g, 0.072 mmol) and TfOH (3 μL, 0.040 mmol) were added and the reaction mixture was allowed to warm up to -25 °C. After complete consumption of the starting materials, it was quenched with sat. $NaHCO_3$ and solid $Na_2S_2O_3$. The mixture was filtered and washed with sat. $NaHCO_3$, H_2O and brine, then dried ($MgSO_4$) and concentrated for silica gel column chromatography purification ($PhCH_3$/EtOAc, 20:1) to give pentasaccharide **8a** (0.054 g, 54 %) as colourless gum. R_f 0.41 ($PhCH_3$/EtOAc 20:1); ^{1}H NMR (600 MHz, $CDCl_3$): δ 8.09-8.07 (m, 4H), 7.65 (dd, J = 7.8, 1.2 Hz, 2H), 7.62 (dd, J = 7.8, 1.2 Hz, 2H), 7.57 (d, J = 7.2 Hz, 1H), 7.54 (d, J = 7.2 Hz, 1H), 7.46 (td, J = 7.8, 3.6 Hz, 4H), 7.39-7.35 (m, 5H), 7.34 (d, J = 5.4 Hz, 2H), 7.29-7.26 (m, 10H), 7.25-7.22 (m, 6H), 7.21-7.17 (m, 10H), 7.15-7.13 (m,

4H), 5.42 (app t, J = 4.8 Hz, 1H), 5.40 (d, J = 9.0 Hz, 1H), 5.38 (d, J = 3.6 Hz, 1H), 5.14 (t, J = 5.4 Hz, 1H), 4.94 (d, J = 10.8 Hz, 1H), 4.88 (dd, J = 10.8, 2.4 Hz, 2H), 4.86-4.83 (m, 3H), 4.76-4.72 (m, 2H), 4.70 (dd, J = 10.2, 3.0 Hz, 3H), 4.68-4.67 (m, 2H), 4.63 (t, J = 7.2 Hz, 2H), 4.40 (d, J = 5.4 Hz, 1H), 4.27 (dd, J = 10.8, 3.6 Hz, 2H), 4.21-4.18 (m, 2H), 4.16 (app d, J = 3.6 Hz, 1H), 4.12 (app dd, J = 12.0, 1.8 Hz, 2H), 4.02 (t, J = 6.6 Hz, 1H), 3.97-3.96 (m, 1H), 3.94-3.93 (m, 1H), 3.91 (app d, J = 5.4 Hz, 1H), 3.90-3.85 (m, 3H), 3.80 (ddd, J = 3.6, 6.6, 10.2 Hz, 2H), 3.72 (q, J = 8.4 Hz, 1H), 3.69-3.67 (m, 2H), 3.53-3.49 (m, 2H), 3.43 (dd, J = 9.6, 3.6 Hz, 2H), 3.34 (s, 3H), 3.15-3.12 (m, 1H), 3.23 (s, 3H), 3.18 (dd, J = 3.6, 10.2 Hz, 1H), 2.35 (s, 3H), 2.05 (s, 3H), 1.03 (s, 9H); ^{13}C NMR (150 MHz, $CDCl_3$): δ 170.8, 170.0, 169.6, 167.6, 165.3, 164.8, 138.3, 138.0, 137.9 (2), 137.8, 137.4, 137.3, 135.9, 135.7, 135.6, 133.6 (2), 133.0, 130.1, 130.0, 129.8-127.8, 125.3, 101.1, 98.5, 98.0, 97.8, 82.5, 80.0, 78.4, 77.8, 77.7, 77.6, 76.2, 75.8, 75.7, 75.3, 75.1, 75.0, 74.8, 74.7, 74.6, 74.1, 73.5, 72.9, 72.5, 71.1, 70.7, 69.4, 68.8, 63.6, 63.3, 62.9, 62.1, 61.8, 61.5, 55.8, 52.4, 51.8, 29.7, 26.8, 21.5, 20.9, 20.8, 19.4; HRMS (m/z): $[M+Na]^+$ calcd. for $C_{109}H_{117}N_9O_{29}SiNa$, 2066.7649; found, 2066.7656.

Compound 8b

Glucosyl donor **5** (0.134 g, 0.183 mmol), azidoglucosyl acceptor **6** (0.105 g, 0.127 mmol) and flame activated AW-300 MS (1.5 g) were suspended in dry CH_2Cl_2 (4 mL) for 1 h at room temperature under N_2 atmosphere. It was then cooled to to -45 °C. NIS (0.052 g, 0.227 mmol) and TfOH (8 μL, 0.073 mmol) were added and the reaction was allowed to warm up slowly to -30 °C. The disaccharide acceptor **7b** (0.100 g, 0.127 mmol) in dry CH_2Cl_2 (2 mL) and MS AW-300 (0.5 g) were stirred for 30 min at room temperature. After consumption of donor **5** (TLC, $PhCH_3$/EtOAc, 20:1), the mixture was again cooled to -45 °C, followed by addition of acceptor **7b**. NIS (0.052 g, 0.227 mmol) and TfOH (8 μL, 0.073 mmol) were added and the reaction mixture was allowed to warm up to -25 °C. After complete consumption of the starting materials, it was quenched with sat. $NaHCO_3$ and solid $Na_2S_2O_3$. The mixture was filtered and washed with sat. $NaHCO_3$, H_2O and brine,then dried ($MgSO_4$) and concentrated for silica gel column chromatography purification ($PhCH_3$/EtOAc, 20:1) to give pentasaccharide **8b** (0.133 g, 49 %) as yellow oil. R_f 0.36 ($PhCH_3$/EtOAc 9:1); ^{1}H NMR (600 MHz, $CDCl_3$): δ 8.16-8.10 (m, 4H), 7.72-7.65 (m, 5H), 7.65-7.58 (m, 2H), 7.52 (dt, J = 9.3, 7.7 Hz, 4H), 7.45-7.39 (m, 5H), 7.39-7.31 (m, 12H), 7.31-7.27 (m, 6H), 7.25-7.22 (m, 6H), 7.20 (dd, J = 10.8, 4.2 Hz, 6H), 5.47-5.44 (m, 2H), 5.44 (d, J = 3.7 Hz, 1H), 5.19 (t, J = 5.4 Hz, 1H), 4.98 (d, J = 10.9 Hz, 1H), 4.93 (dd, J = 10.9, 1.8 Hz, 2H), 4.91-4.86 (m, 4H), 4.80 (d, J = 11.1 Hz, 1H), 4.72 (t, J = 10.1 Hz, 2H), 4.68 (d, J = 8.0 Hz, 1H), 4.50 (d, J = 4.9 Hz, 1H), 4.31-4.26 (m, 2H), 4.24-4.16 (m, 3H), 4.09 (t, J = 5.8 Hz, 1H), 4.02-3.95 (m, 3H), 3.94-3.90 (m, 3H), 3.91-3.84 (m, 3H), 3.76 (ddd, J = 14.3, 10.3, 5.8 Hz, 2H), 3.70 (dt, J = 10.0, 2.7 Hz, 1H), 3.57-3.52 (m, 1H), 3.44 (q, J = 3.9, 3.5 Hz, 2H), 3.42 (s, 3H), 3.40 (s, 3H), 3.27 (s, 3H), 3.22 (dd, J = 10.2, 3.7 Hz, 1H), 2.79 (m, 2H), 2.65 (m, 2H), 2.40 (s, 6H), 2.19 (s, 3H), 2.05 (s, 3H), 1.09 (s, 9H); ^{13}C NMR (150 MHz, $CDCl_3$): δ 206.4, 172.4, 170.4, 169.5, 167.6, 165.3, 164.8, 138.9-137.2, 135.9-135.6, 133.9, 133.6, 133.5, 132.9, 129.8-127.4, 125.3, 101.0, 98.5, 98.5, 98.0, 97.7, 82.5, 80.1, 78.4, 77.8, 77.7, 77.5, 75.9, 75.7, 75.2, 75.1, 75.0, 74.9, 74.8,

74.7, 74.6, 73.9, 73.5, 72.5, 70.6, 70.3, 69.4, 68.8, 63.5, 63.4, 62.8, 62.3, 61.8, 61.3, 55.4, 52.4, 51.8, 37.9, 29.9, 27.9, 26.8, 21.5, 20.8, 19.4; HRMS (m/z): $[M+Na]^+$ calcd. for $C_{112}H_{121}N_9O_{30}SiNa$, 2123.7912; found, 2123.7870.

c

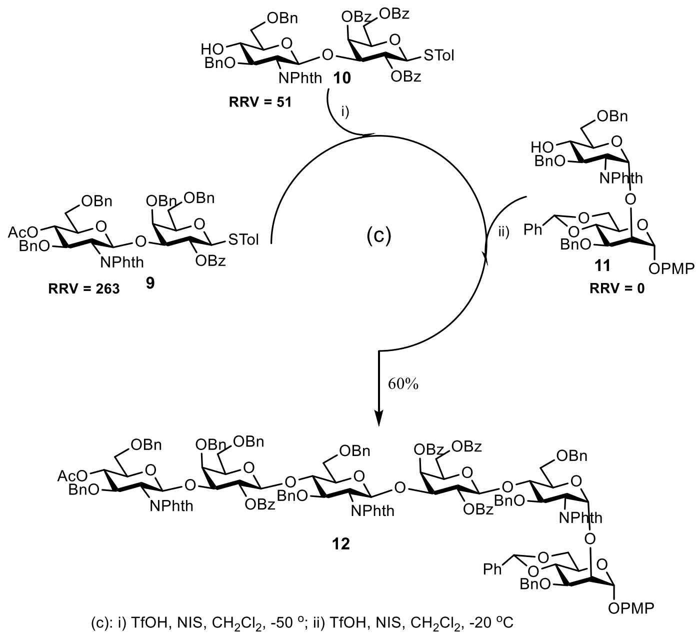

(c): i) TfOH, NIS, CH_2Cl_2, -50 °; ii) TfOH, NIS, CH_2Cl_2, -20 °C

Compounds 9-11

Please see reference[4].

Compound 12

A mixture of compound **9** (0.12 g, 0.111 mmol), compound **10** (0.10 g, 0.093 mmol) and freshly activated molecular sieves was mixed with anhydrous CH_2Cl_2 (1 mL) and the mixture was stirred for 0.5 h at room temperature under argon. The solution was cooled down to -50 °C, and NIS (25 mg, 0.111 mmol) and TfOH (2 μL, 0.028 mmol) were added. After stirring for 1 h, compound **10** was consumed completely as monitored by TLC. A solution of compound **11** (0.087 g, 0.093 mmol) in anhydrous CH_2Cl_2 (1 mL) was then added to the mixture. The reaction temperature was warmed up to -20 °C and NIS (42 mg, 0.186 mmol) and TfOH (2 μL, 0.028 mmol) were added sequentially, and the reaction mixture was stirred for 1 h and quenched by Et_3N. The mixture was filtered through Celite, washed with aqueous $Na_2S_2O_3$, aqueous $NaHCO_3$ and water, and the solution was dried over Na_2SO_4 and concentrated in *vacuo*. The crude residue was purified by flash column chromatography (acetone/toluene = 8/92, R_f = 0.25) to provide compound **12** as oil in 60% overall yield. ^1H NMR (600 MHz, $CDCl_3$): δ 8.06 – 8.01 (m, 2H), 7.98-7.93 (m, 2H), 7.63-7.50 (m, 9H), 7.47-7.39 (m, 8H), 7.38-7.25 (m, 22H), 7.23-7.07 (m, 23H), 6.88-6.80 (m, 8H), 6.76-6.72 (m, 2H), 6.70-6.62 (m, 7H), 6.63-6.57 (m, 2H), 6.55-6.49 (m, 2H), 5.55 (d, J = 3.6 Hz, 1H), 5.37-5.32 (m, 2H), 5.24 (dd, J = 9.8, 7.8 Hz, 1H), 5.17 (d, J = 8.5 Hz, 1H), 5.06-4.91 (m, 5H), 4.73 (d, J = 12.5 Hz, 1H), 4.66 (d, J = 11.9 Hz, 1H),

4.63 (d, J = 12.2 Hz, 1H), 4.55 (d, J = 12.2 Hz, 1H), 4.51-4.36 (m, 11H), 4.23-4.12 (m, 6H), 4.12-4.05 (m, 3H), 4.03-3.96 (m, 2H), 3.94-3.80 (m, 7H), 3.79-3.74 (m, 1H), 3.74-3.65 (m, 6H), 3.63-3.53 (m, 3H), 3.52-3.45 (m, 2H), 3.44-3.35 (m, 3H), 3.30-3.19 (m, 4H), 3.13-3.04 (m, 2H), 2.96-2.90 (m, 1H), 1.92 (s, 3H, -C(O)CH_3); ^{13}C NMR (150 MHz, $CDCl_3$): δ 169.7, 168.6, 167.9, 167.4, 166.8, 166.3, 165.7, 164.3, 164.1, 154.8, 149.5, 139.0, 138.7, 138.6, 138.5, 138.4, 138.2, 138.1, 137.7, 137.6, 137.4, 133.6, 133.5, 133.1, 132.9, 132.8, 131.8, 131.3, 130.8, 130.0, 129.8, 129.8, 129.7, 129.5, 129.4, 128.8, 128.7, 128.5, 128.4, 128.4, 128.3, 128.3, 128.2, 128.2, 128.0, 128.00, 128.0, 127.8, 127.6, 127.6, 127.6, 127.6, 127.5, 127.3, 127.3, 127.2, 126.7, 126.4, 126.0, 123.3, 122.9, 116.9, 114.4, 101.4, 100.6, 100.3, 99.4, 99.3, 96.8, 96.1, 80.1, 78.0, 77.9, 77.9, 76.6, 76.4, 76.3, 76.1, 74.8, 74.8, 74.7, 74.7, 74.4, 74.2, 73.9, 73.8, 73.5, 73.3, 73.3, 73.3, 72.4, 72.0, 71.7, 71.2, 70.4, 70.0, 68.4, 68.1, 67.5, 64.1, 62.8, 55.7, 55.6, 55.4, 55.3, 20.8. HRMS (m/z): $[M+2Na]^+$ calcd. for $C_{167}H_{153}N_3O_{40}Na_2$, 2885.9826; found, 1443.4924.

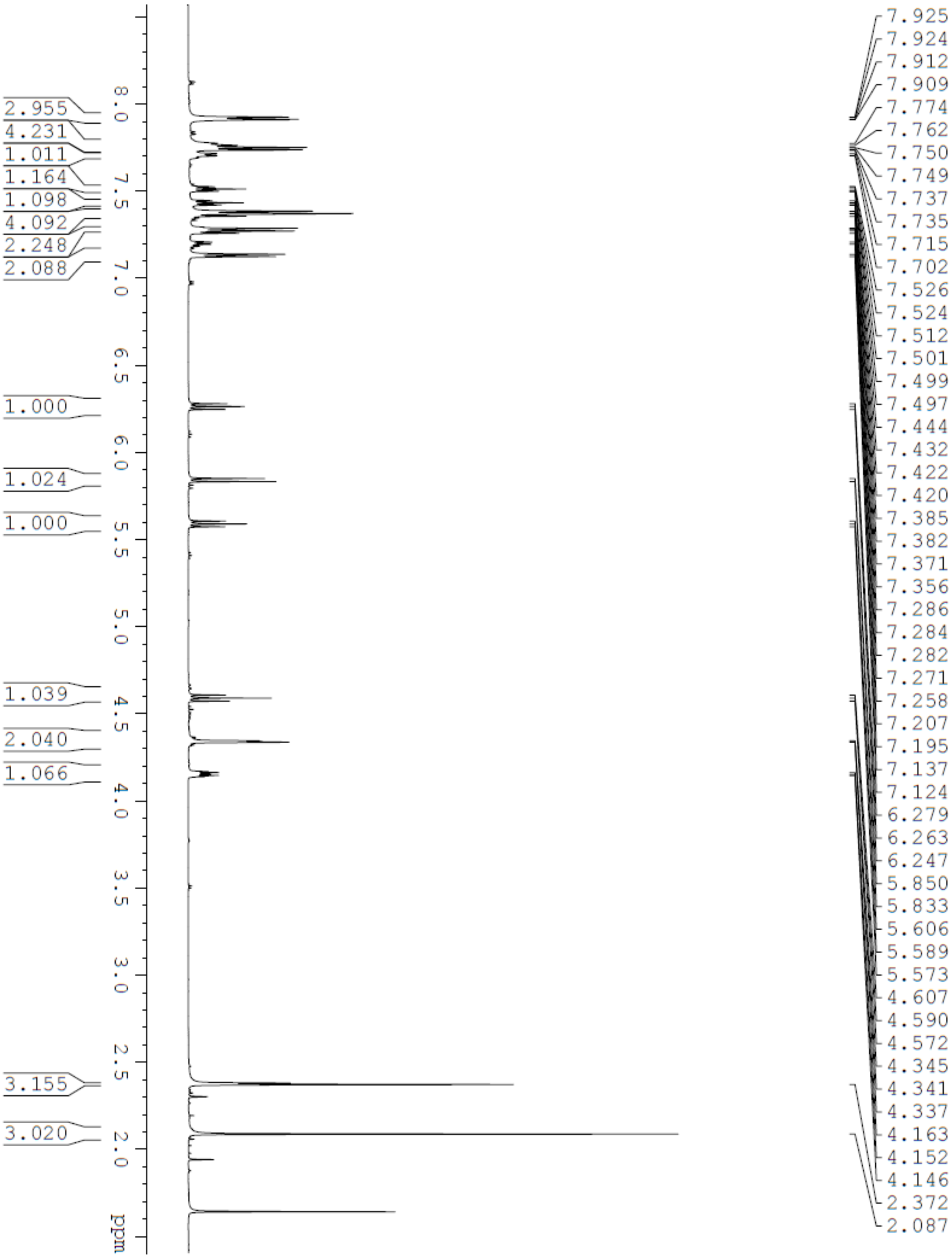

Supplementary Figure 9 | ^{1}H-NMR Spectrum of Dx1

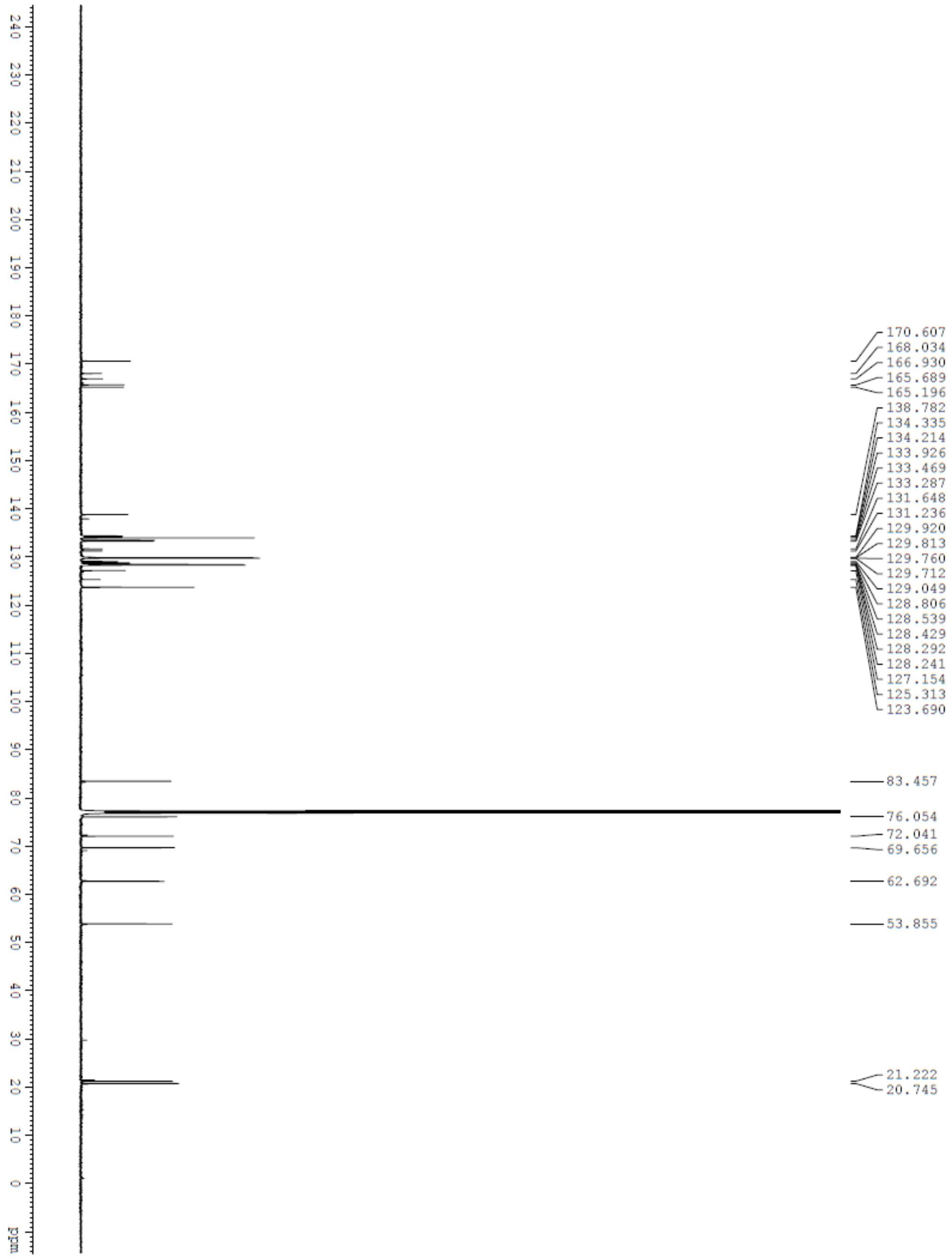

Supplementary Figure 10 | ^{13}C-NMR Spectrum of Dx1

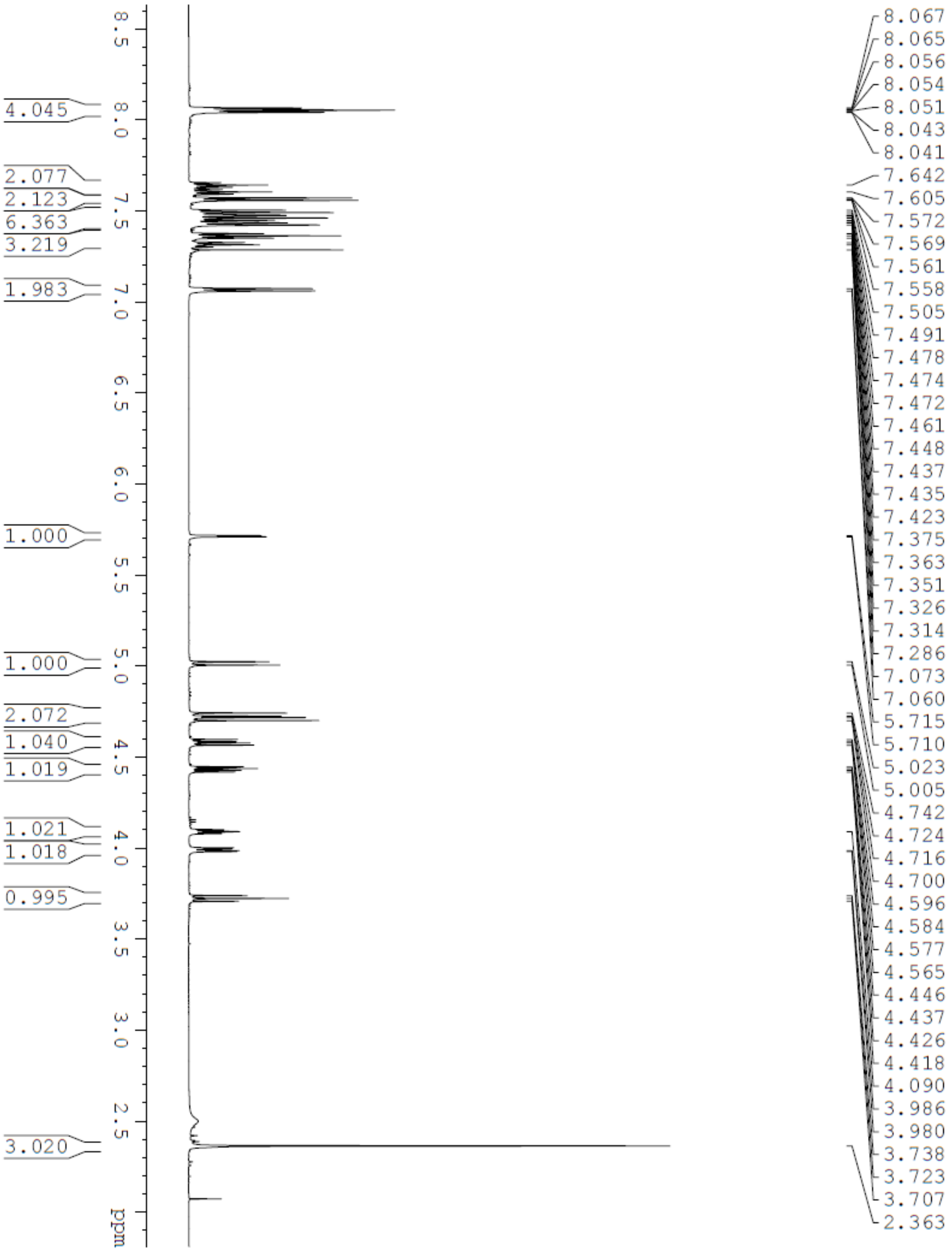

Supplementary Figure 11 | ^{1}H-NMR Spectrum of Dx3

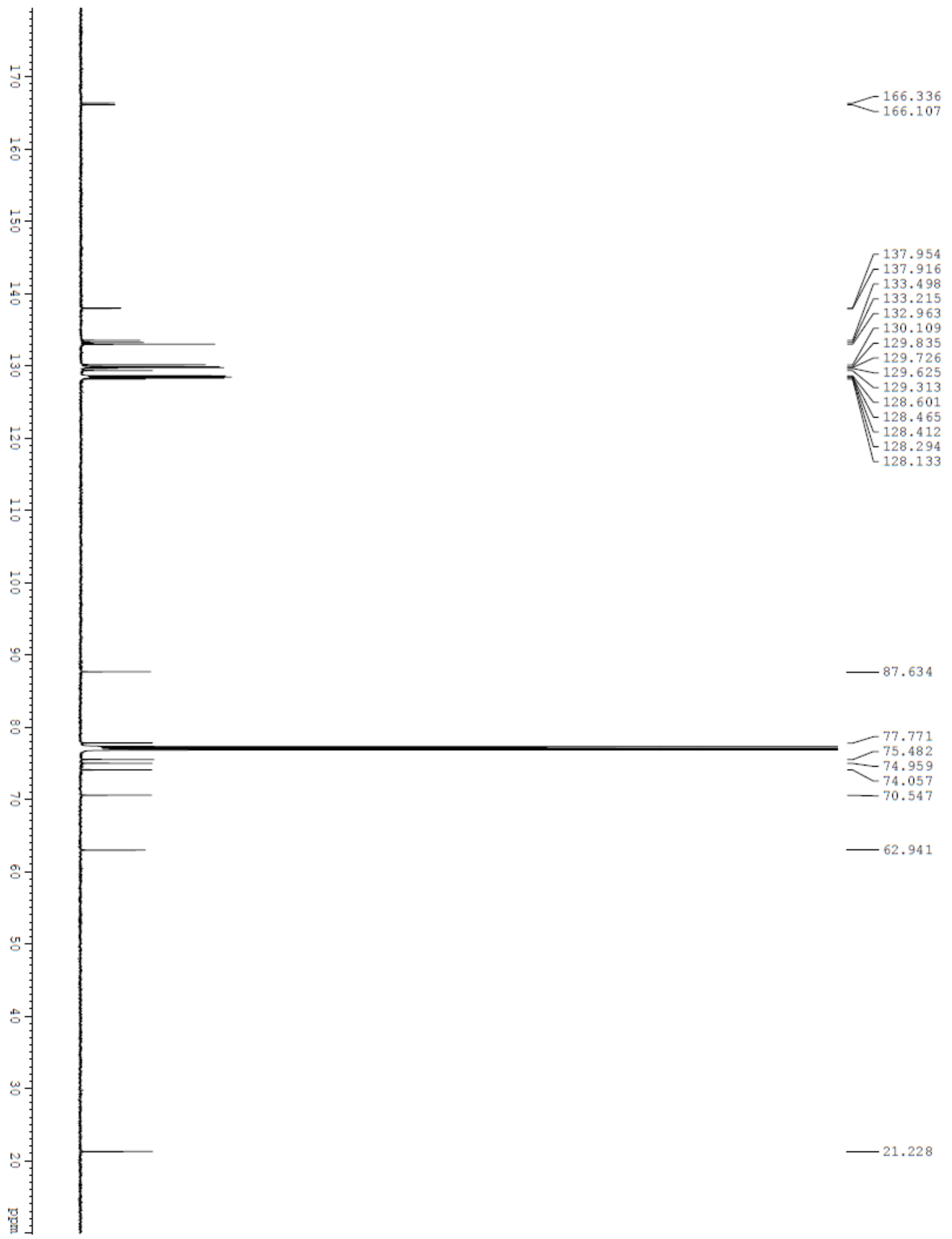

Supplementary Figure 12 | ^{13}C-NMR Spectrum of Dx3

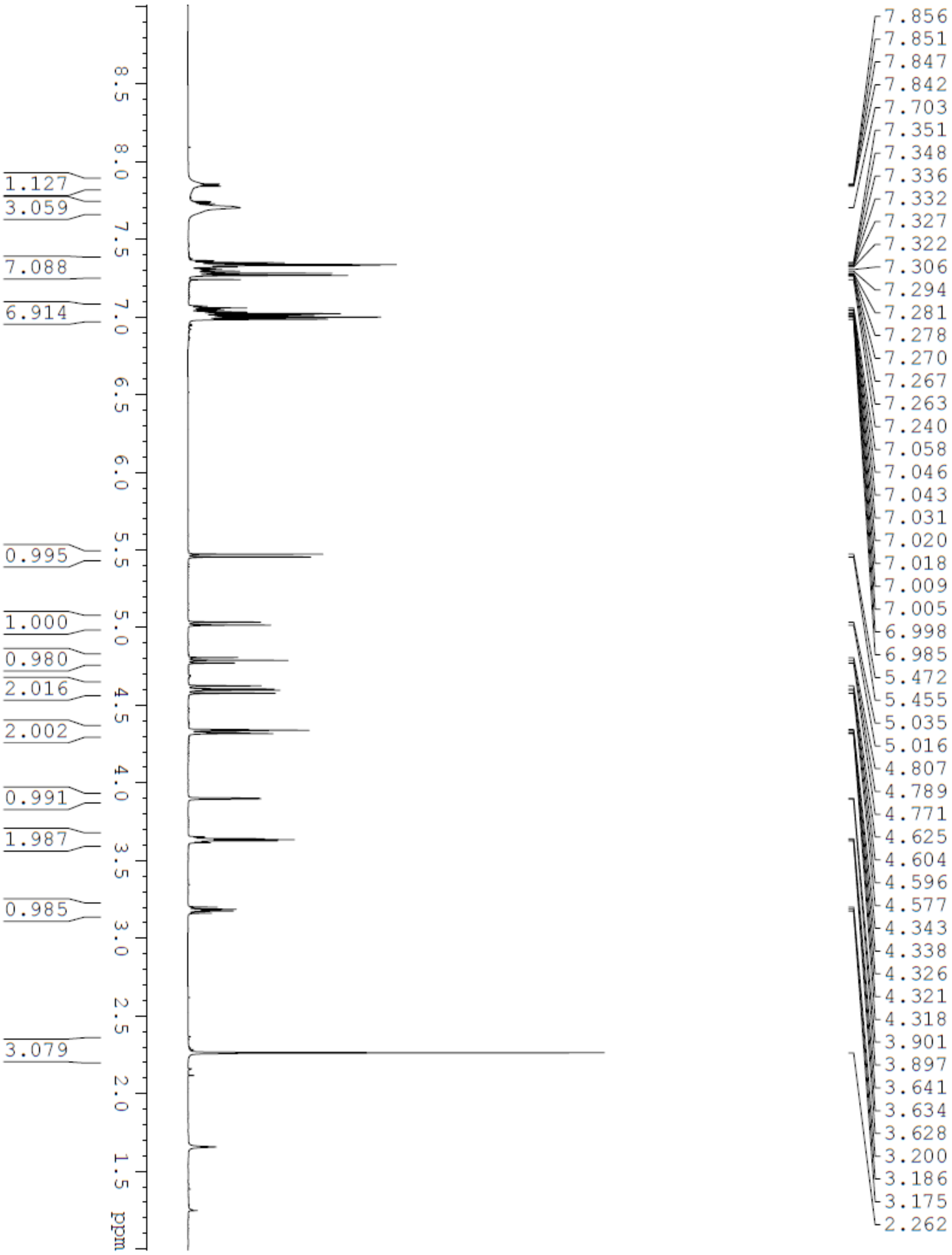

Supplementary Figure 13 | ^{1}H-NMR Spectrum of Dx4

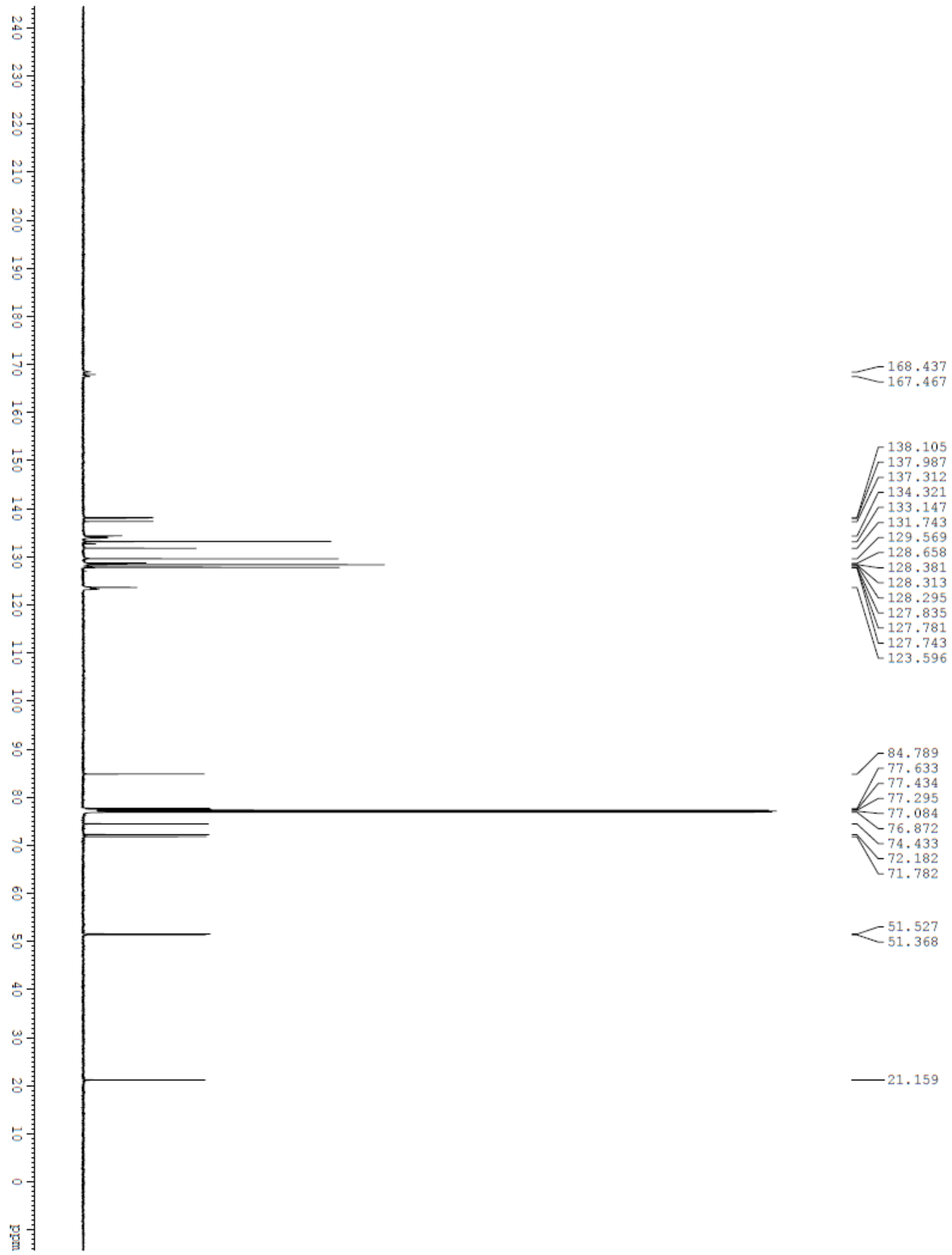

Supplementary Figure 14 | ^{13}C-NMR Spectrum of Dx4

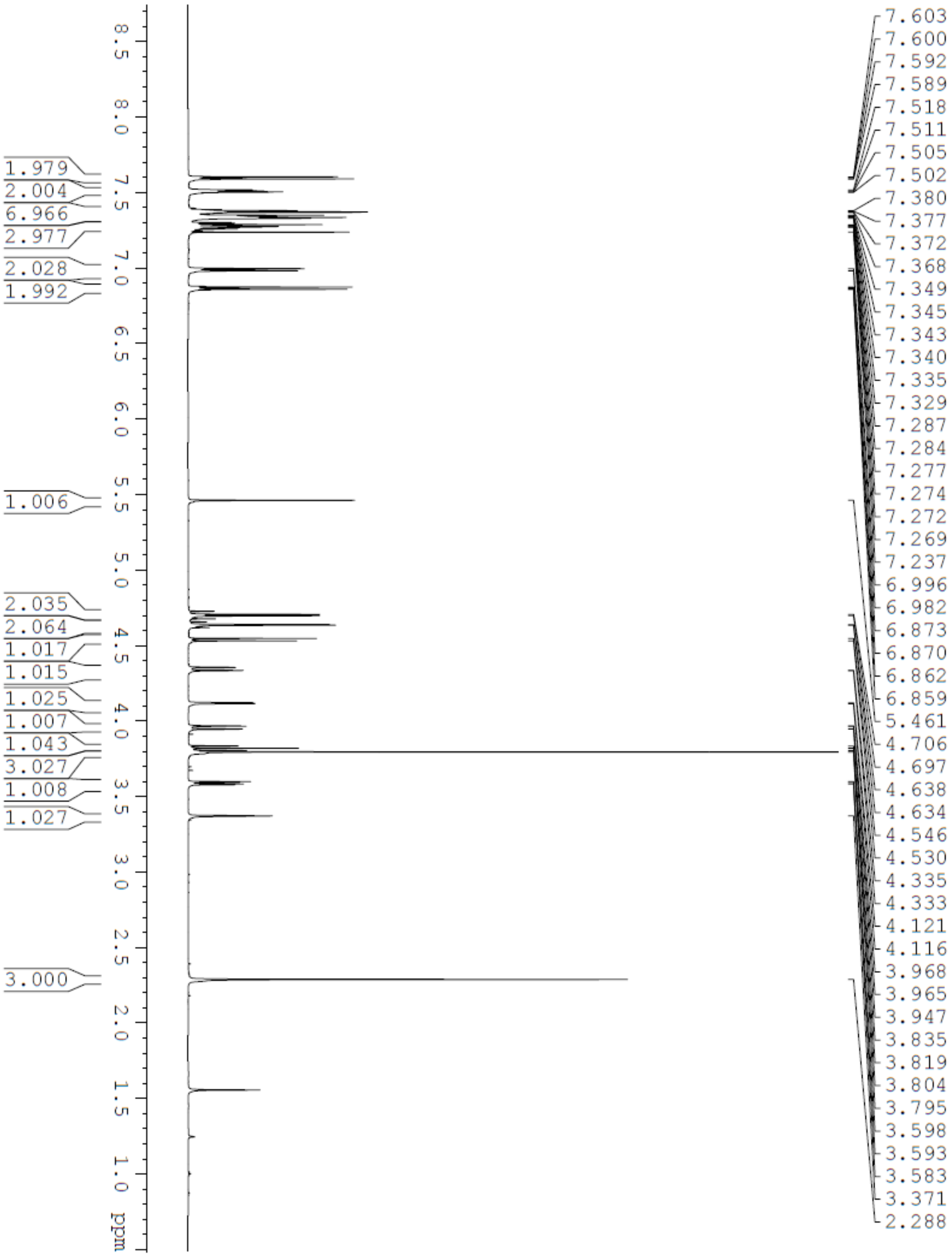

Supplementary Figure 15 | ^{1}H-NMR Spectrum of Dx5

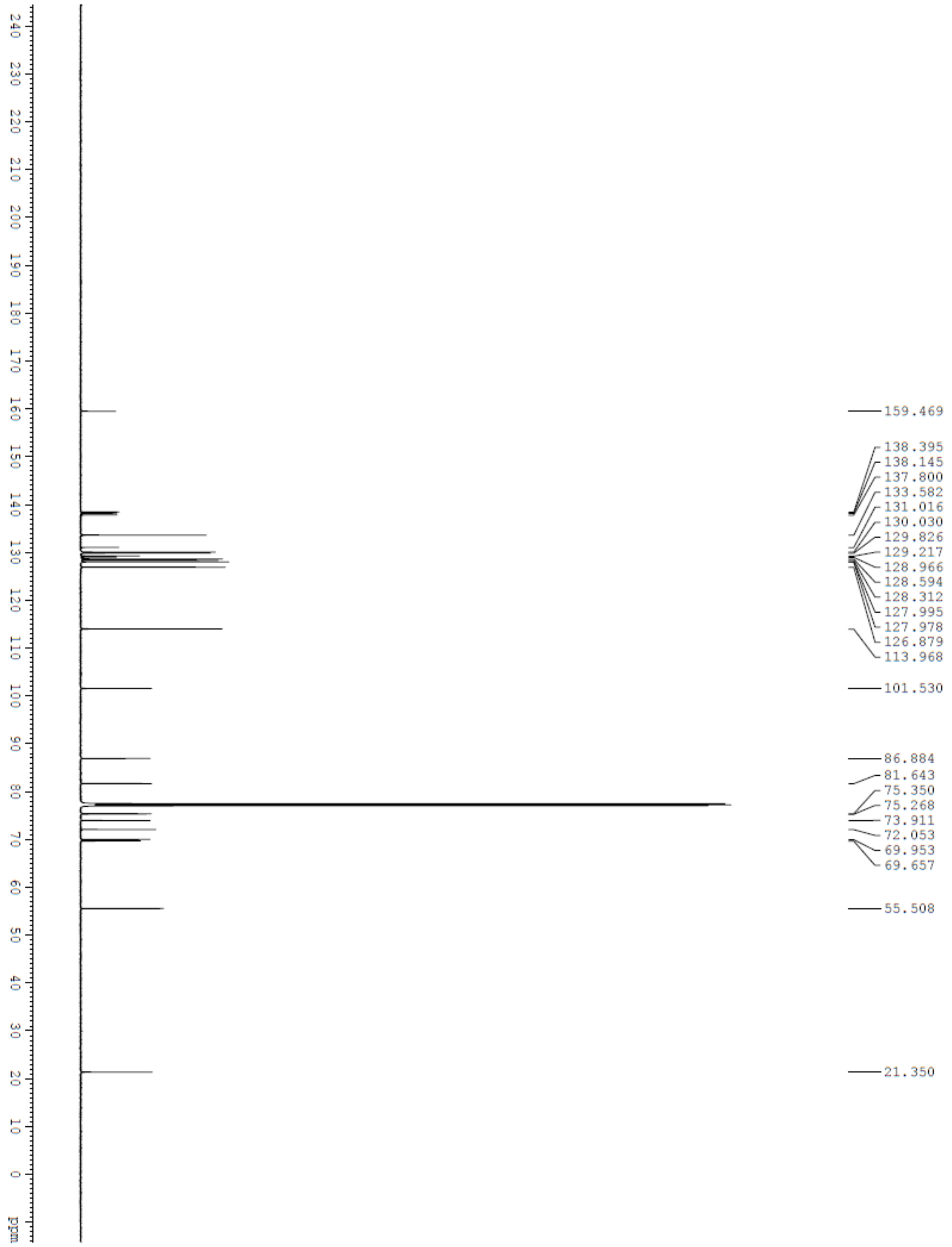

Supplementary Figure 16 | ^{13}C-NMR Spectrum of Dx5

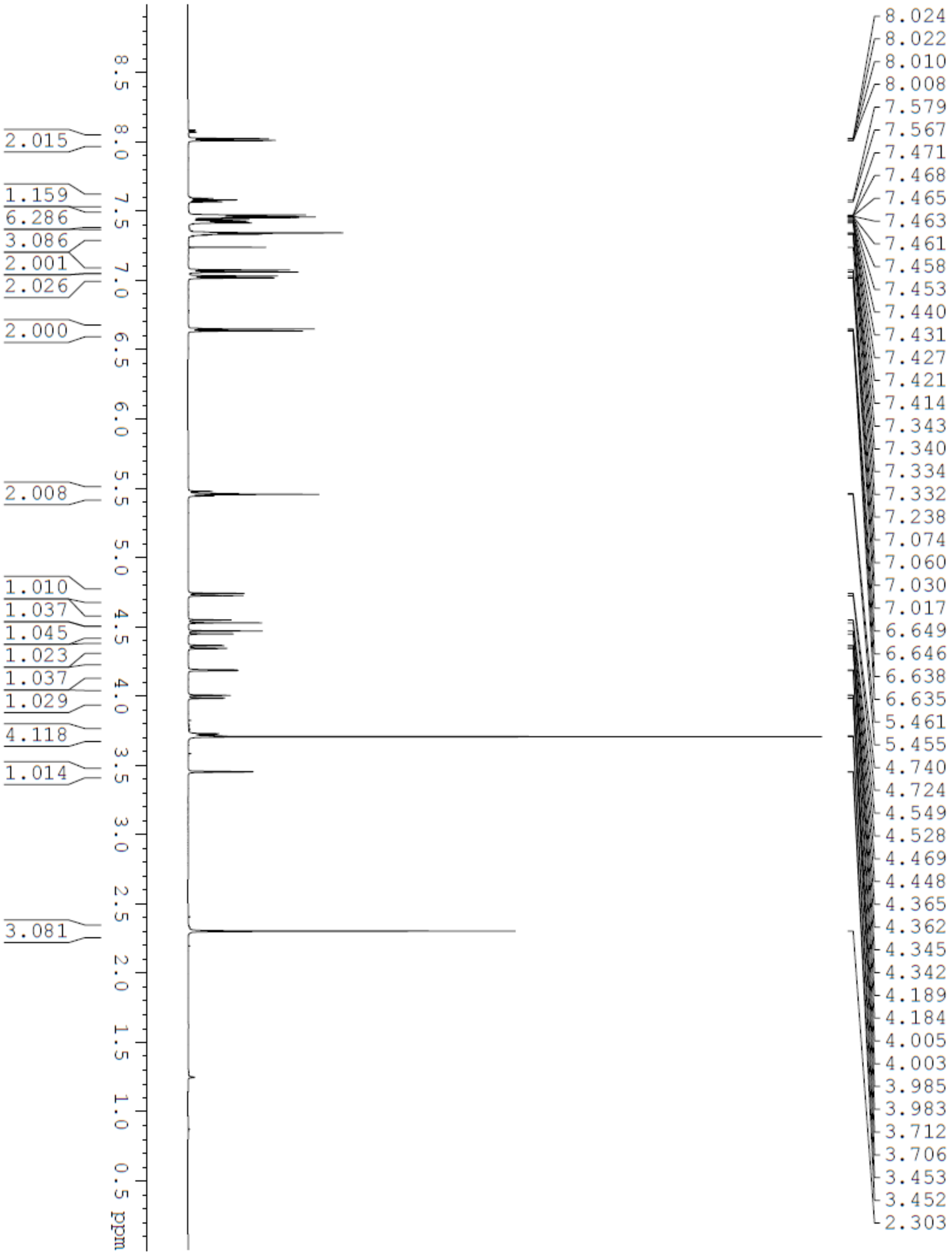

Supplementary Figure 17 | ^{1}H-NMR Spectrum of Dx6

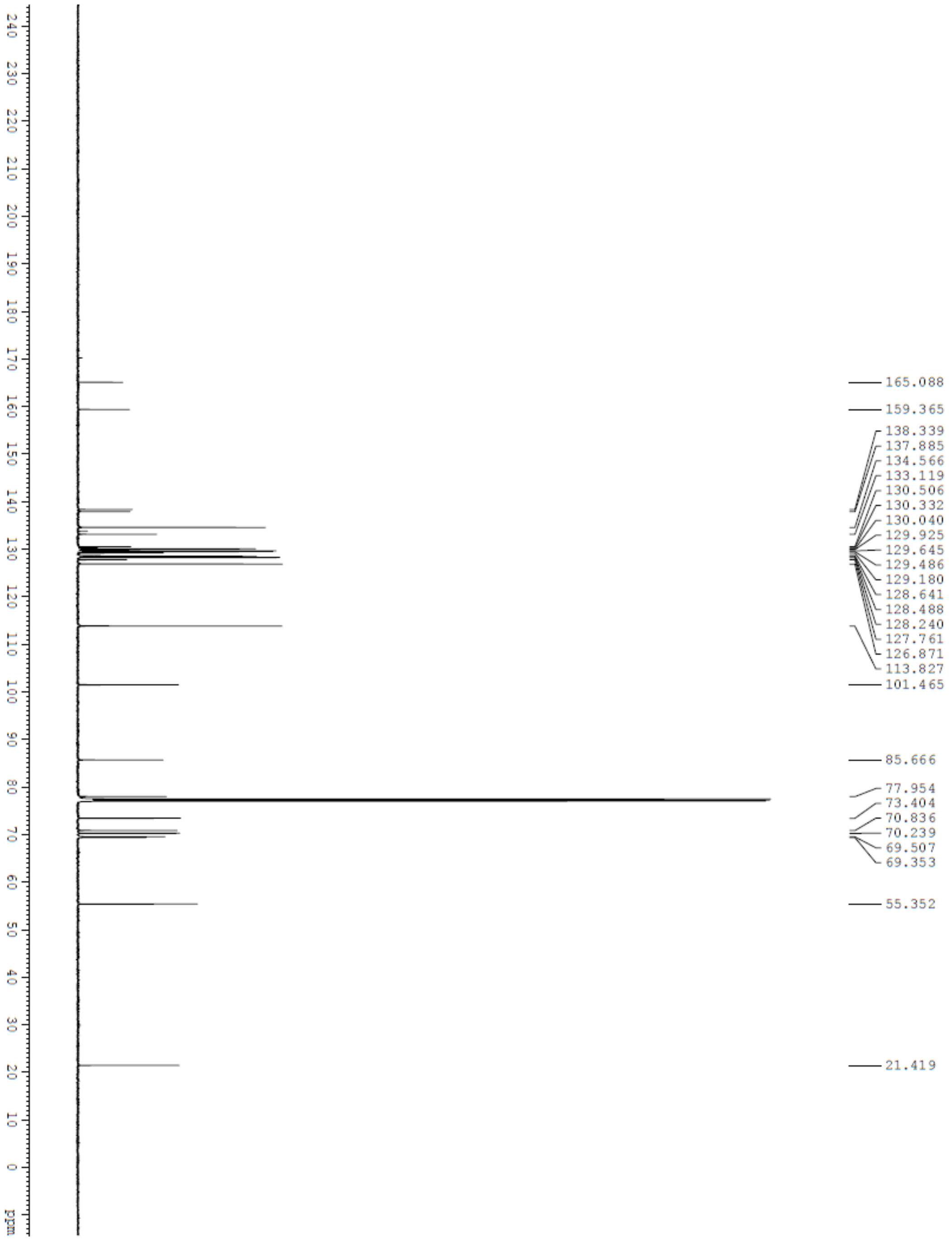

Supplementary Figure 18 | ^{13}C-NMR Spectrum of Dx6

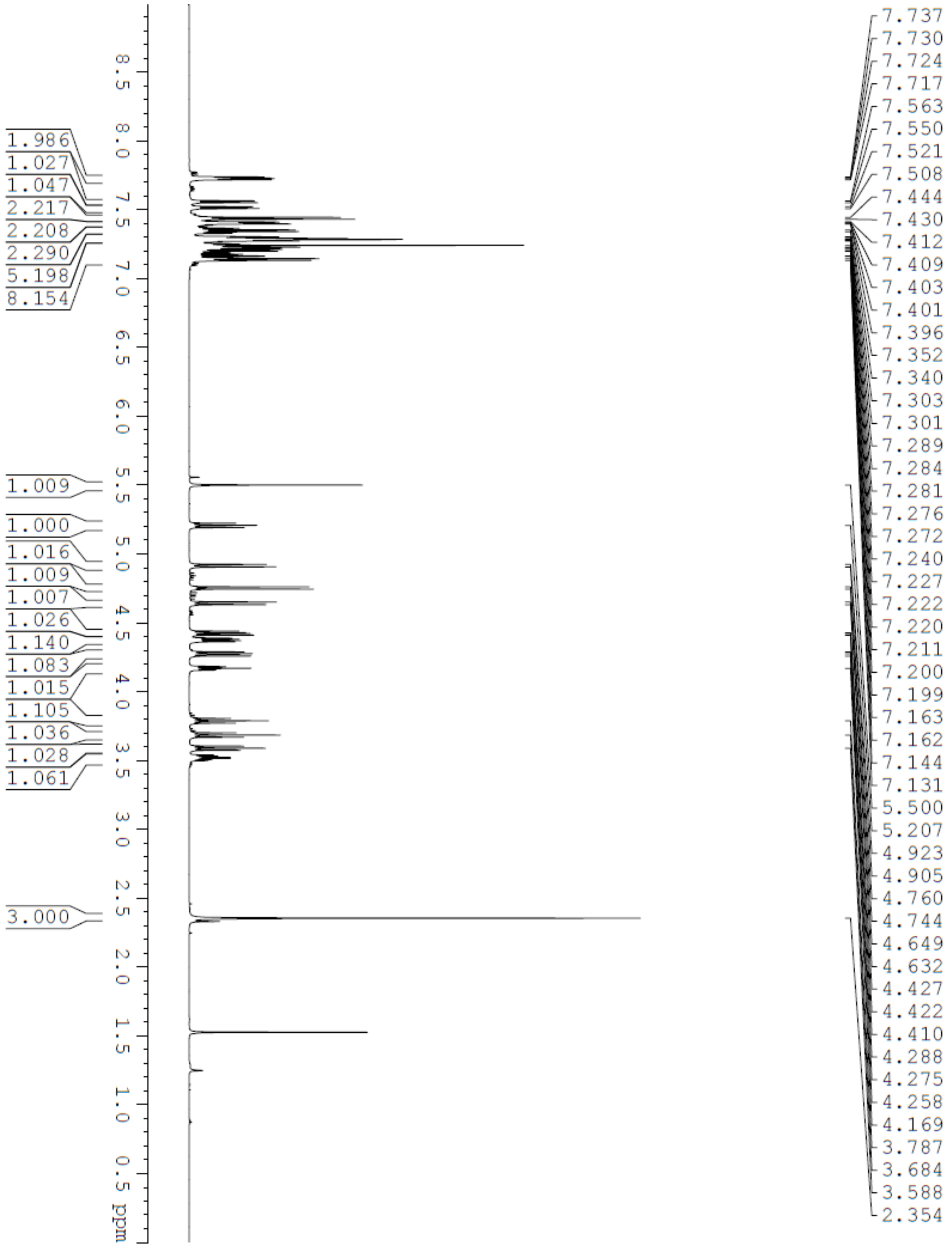

Supplementary Figure 19 | ^{1}H-NMR Spectrum of Dx7

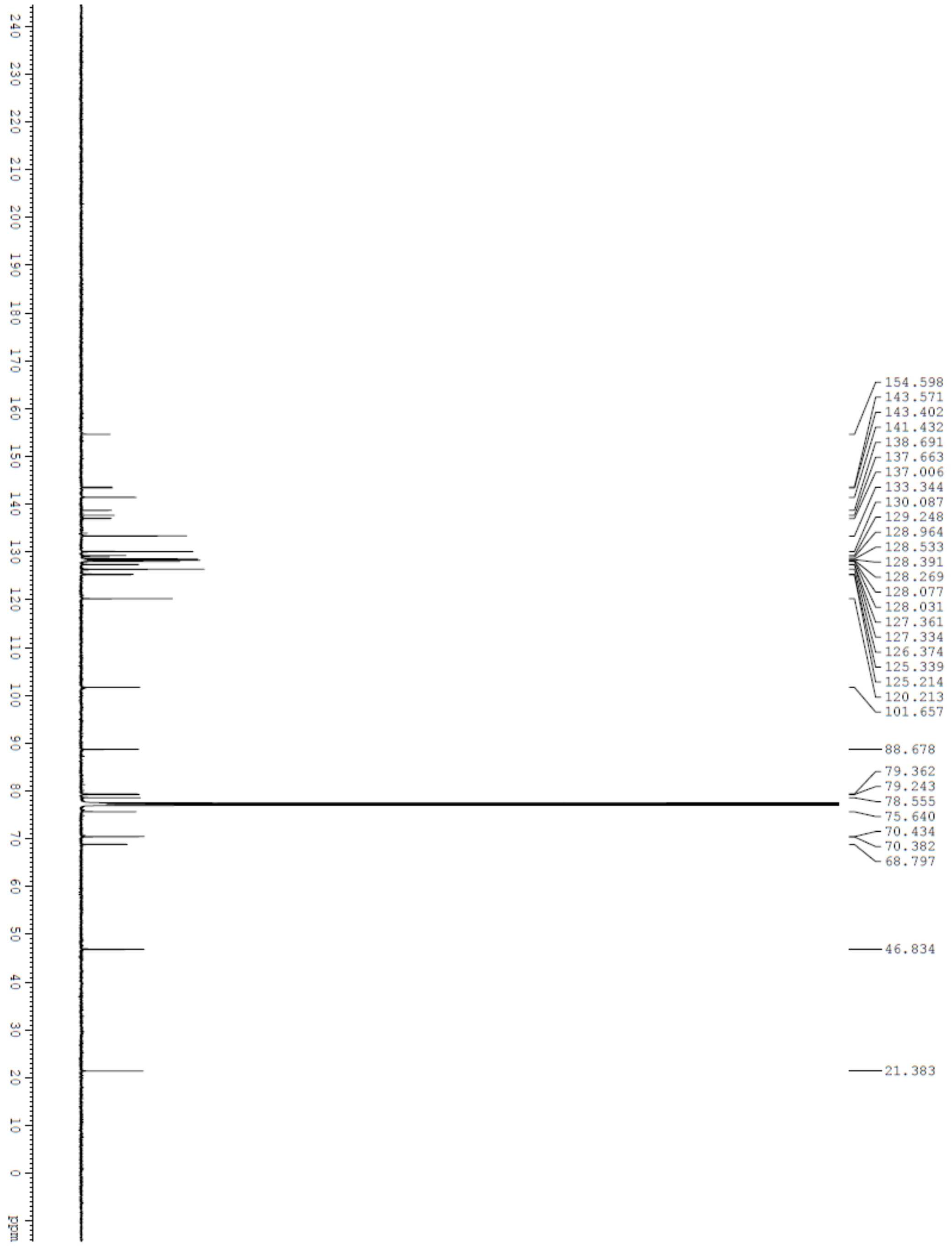

Supplementary Figure 20 | ^{13}C-NMR Spectrum of Dx7

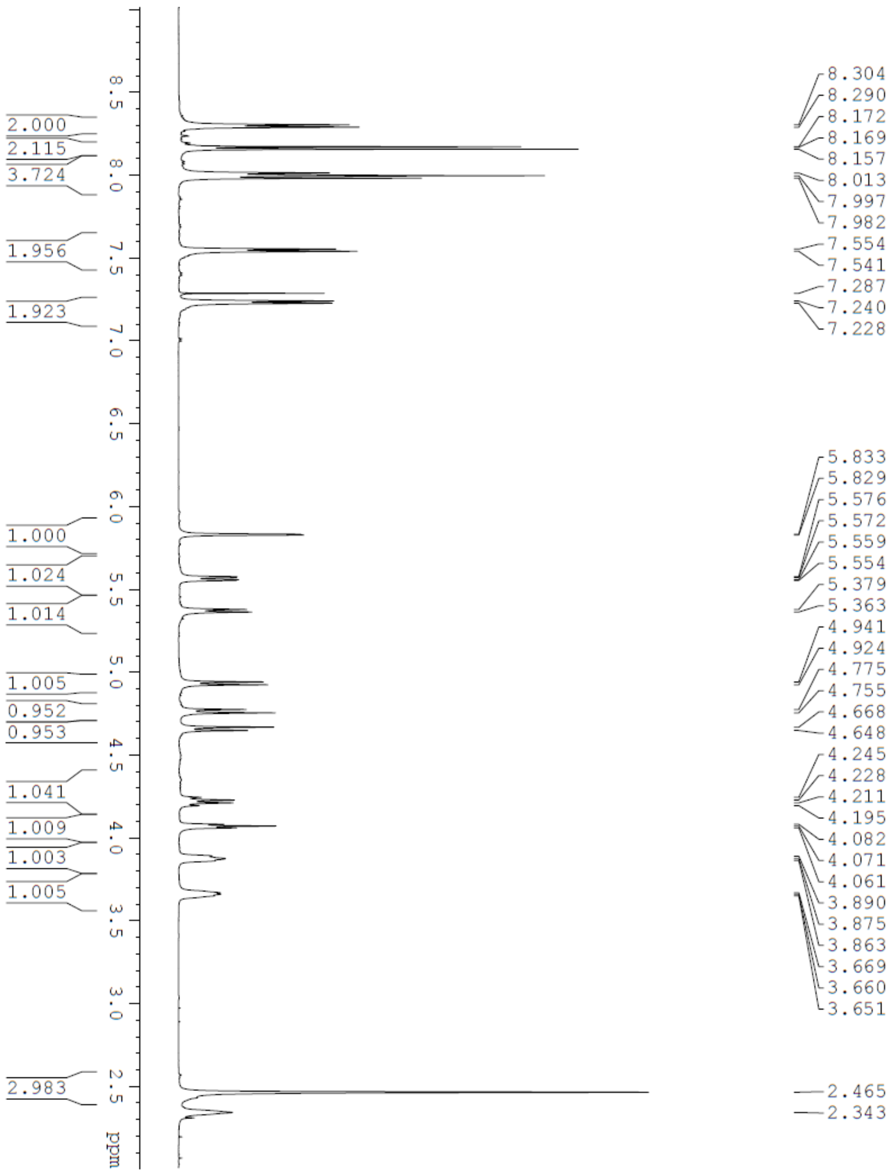

Supplementary Figure 21 | ^{1}H-NMR Spectrum of Compound 2

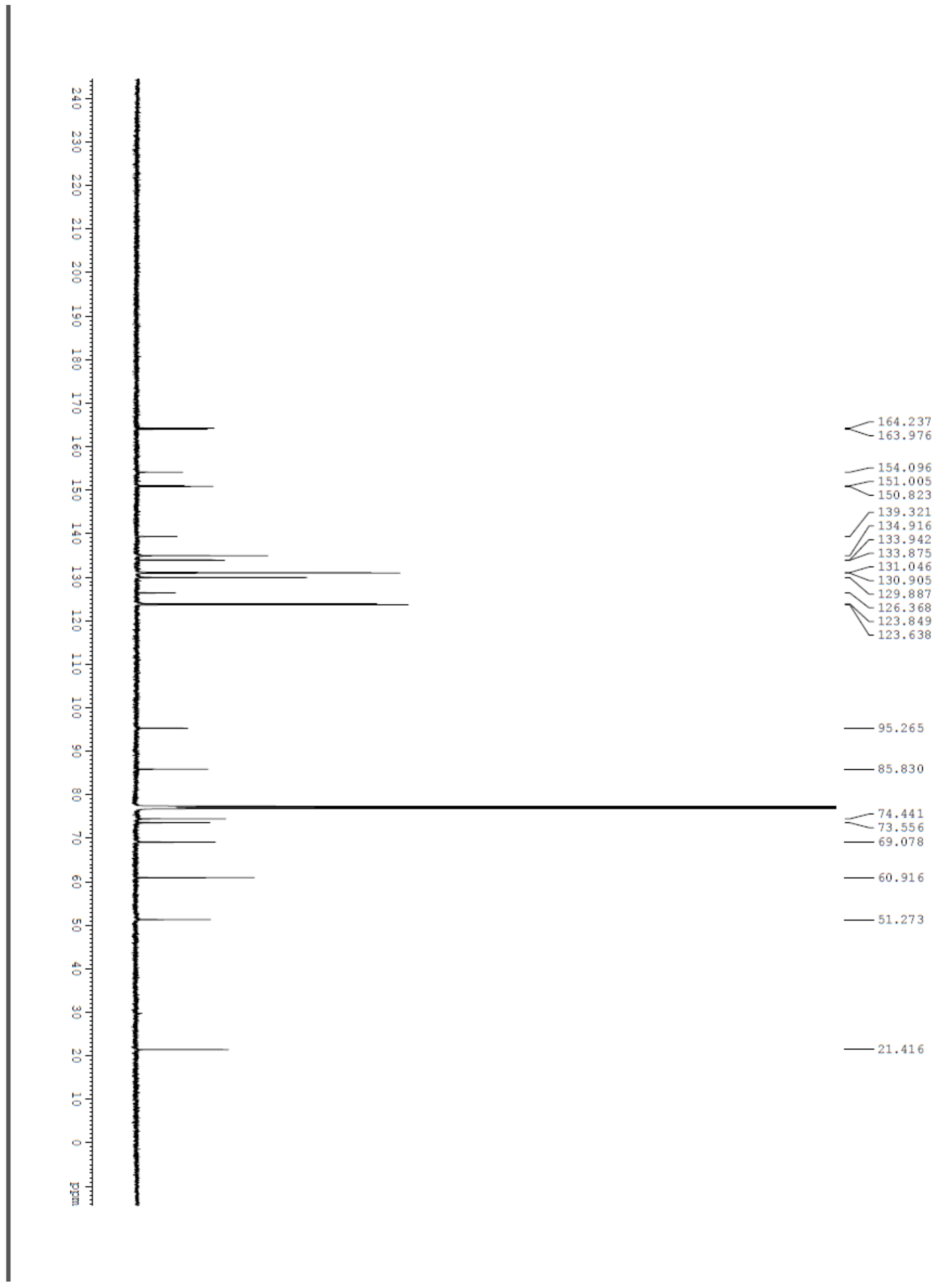

Supplementary Figure 22 | ^{13}C-NMR Spectrum of Compound 2

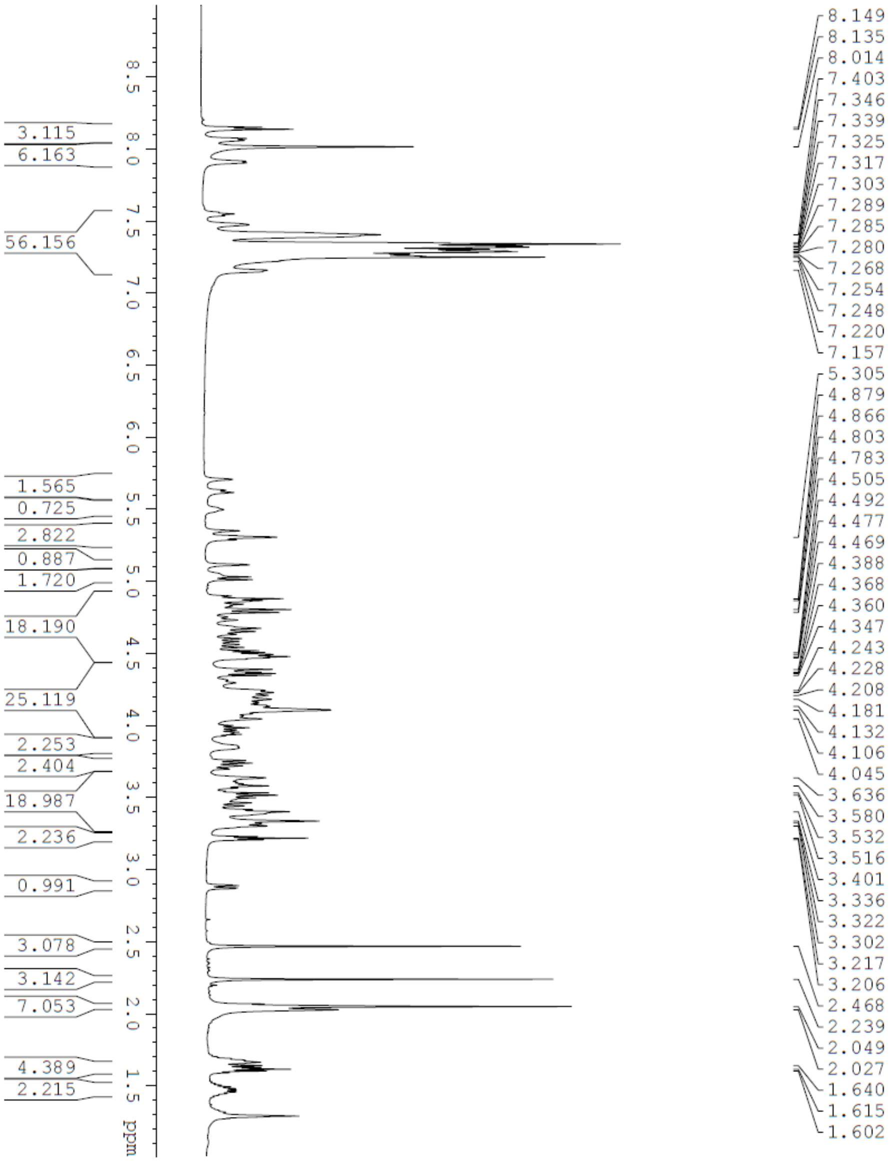

Supplementary Figure 23 | ^{1}H-NMR Spectrum of Compound 4

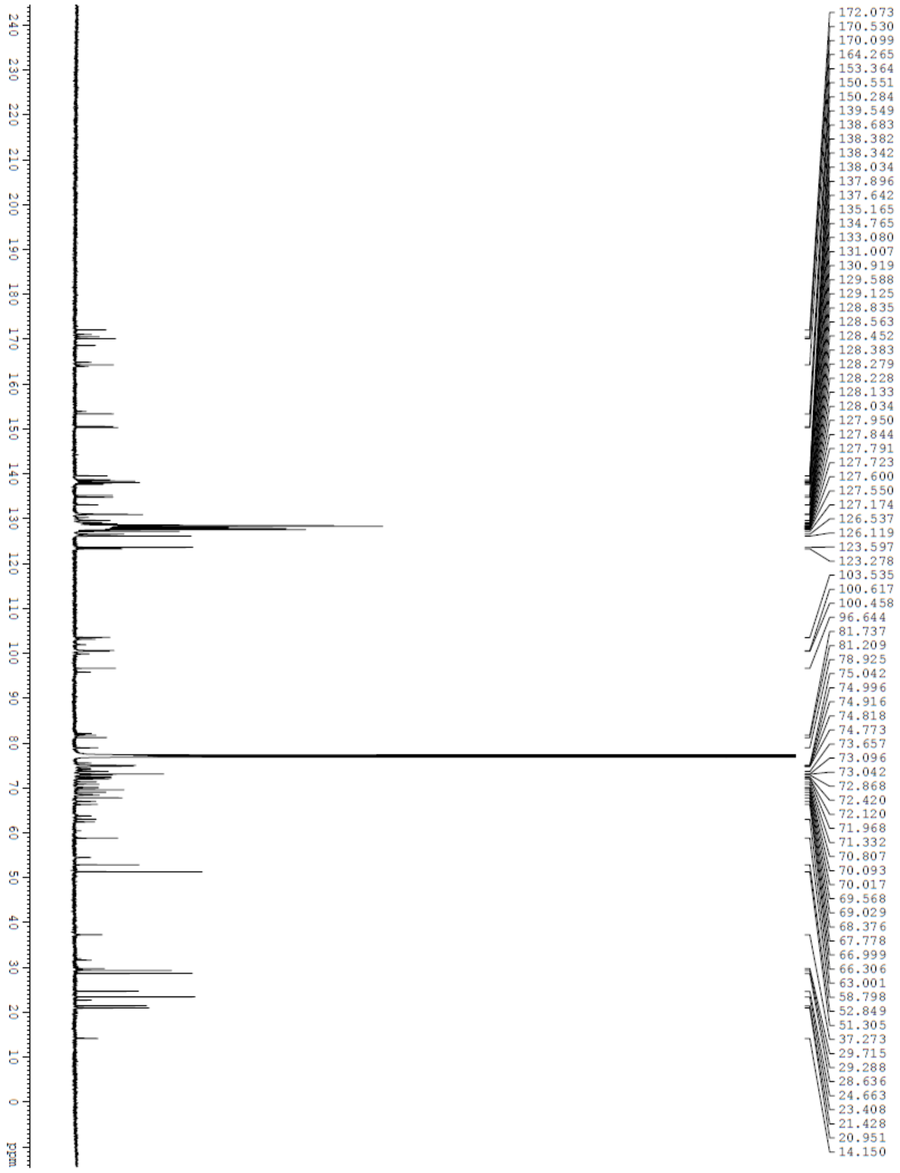

Supplementary Figure 24 | ^{13}C-NMR Spectrum of Compound 4

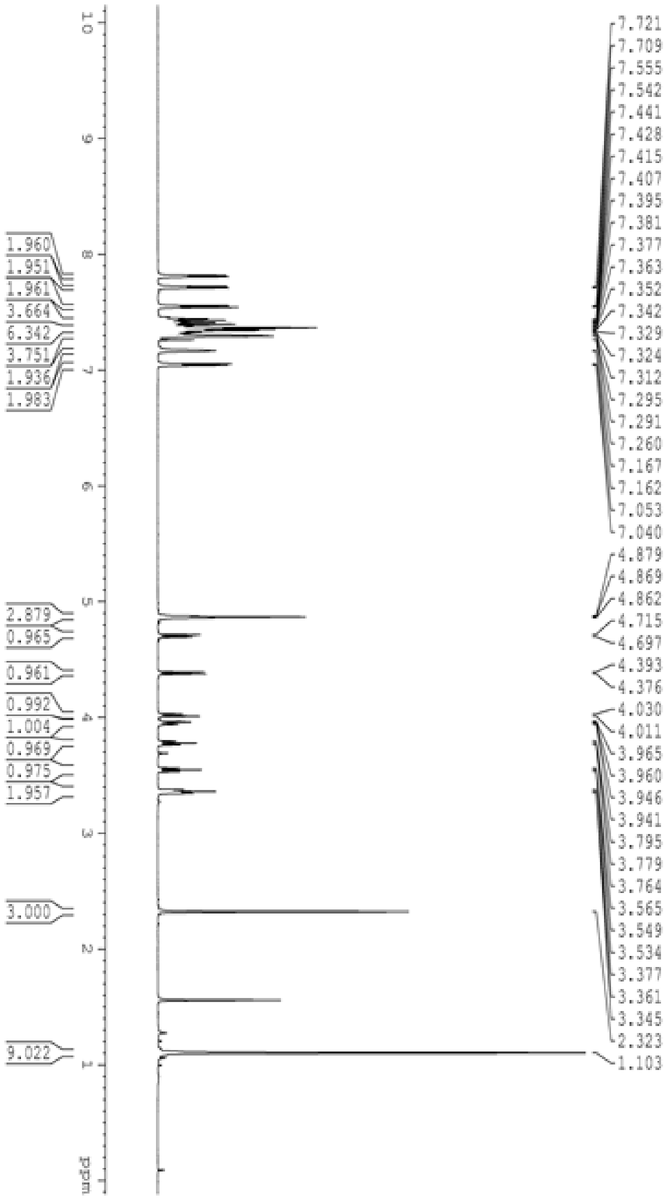

Supplementary Figure 25 | ^{1}H-NMR Spectrum of Compound 5

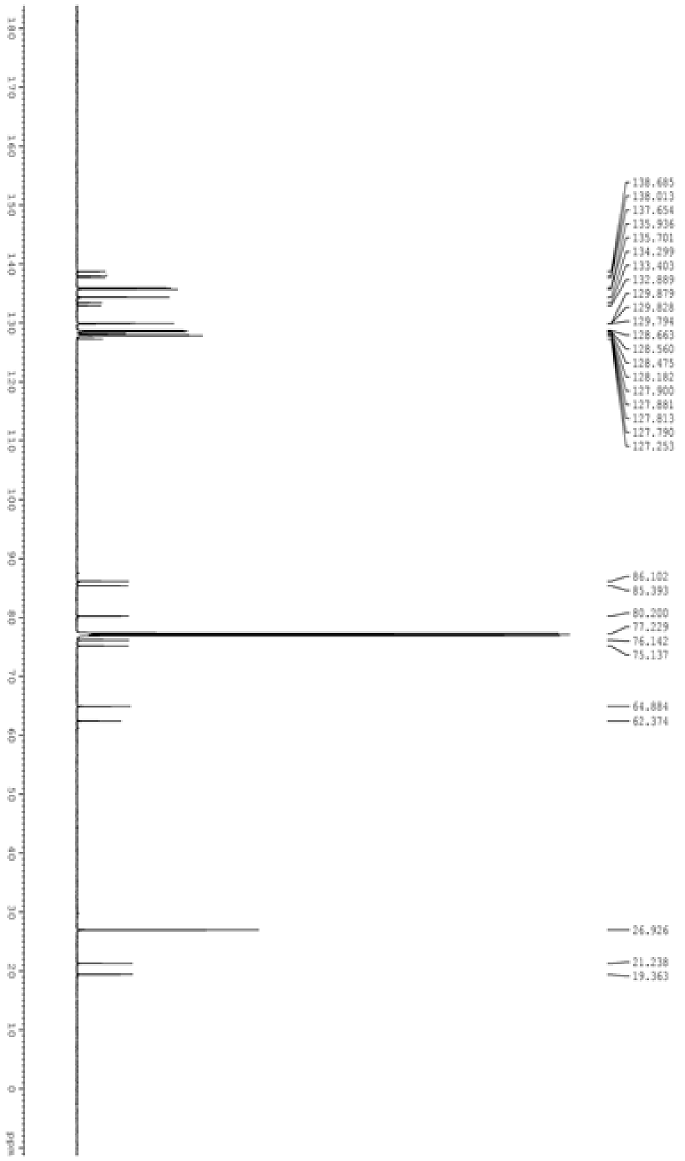

Supplementary Figure 26 | ^{13}C-NMR Spectrum of Compound 5

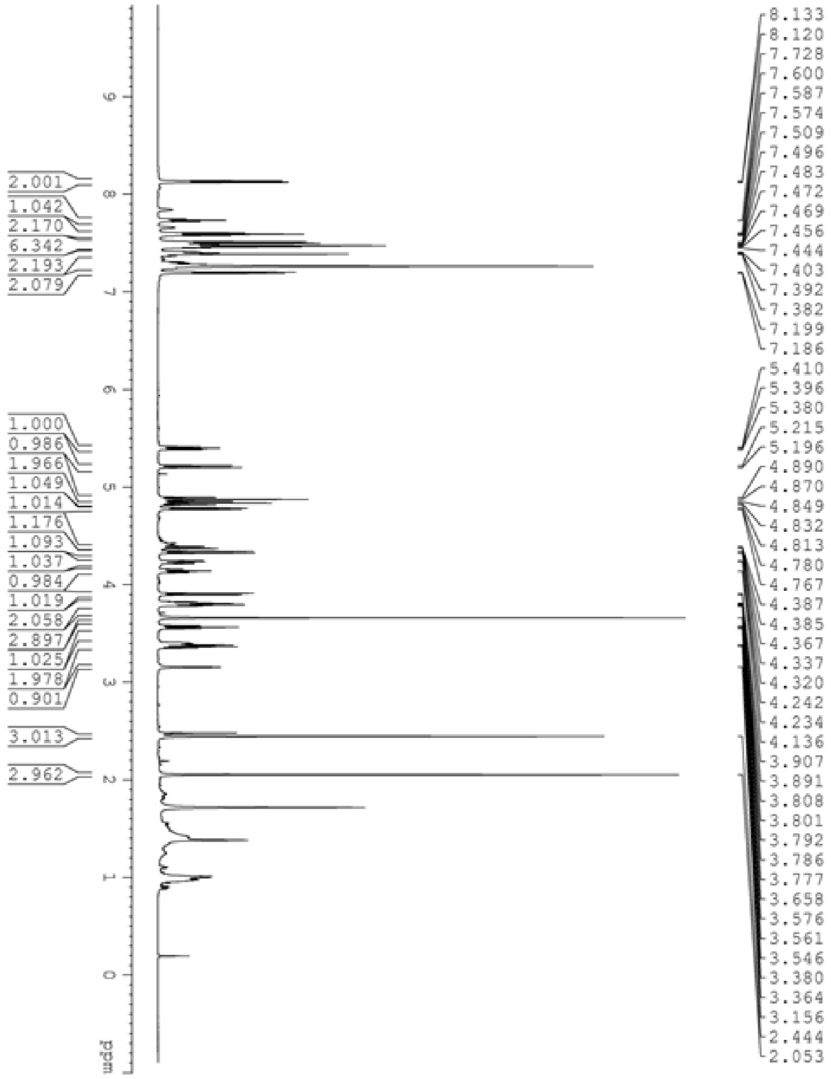

Supplementary Figure 27 | ^{1}H-NMR Spectrum of Compound 6

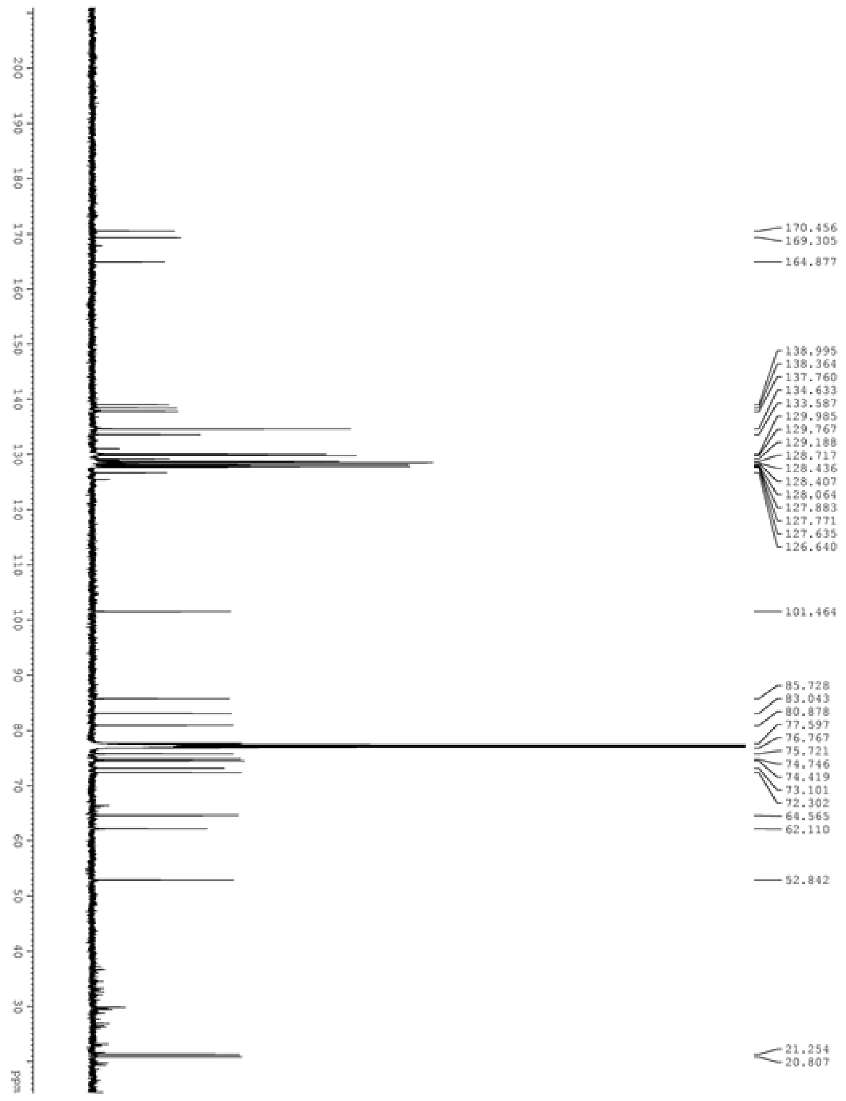

Supplementary Figure 28 | ^{13}C-NMR Spectrum of Compound 6

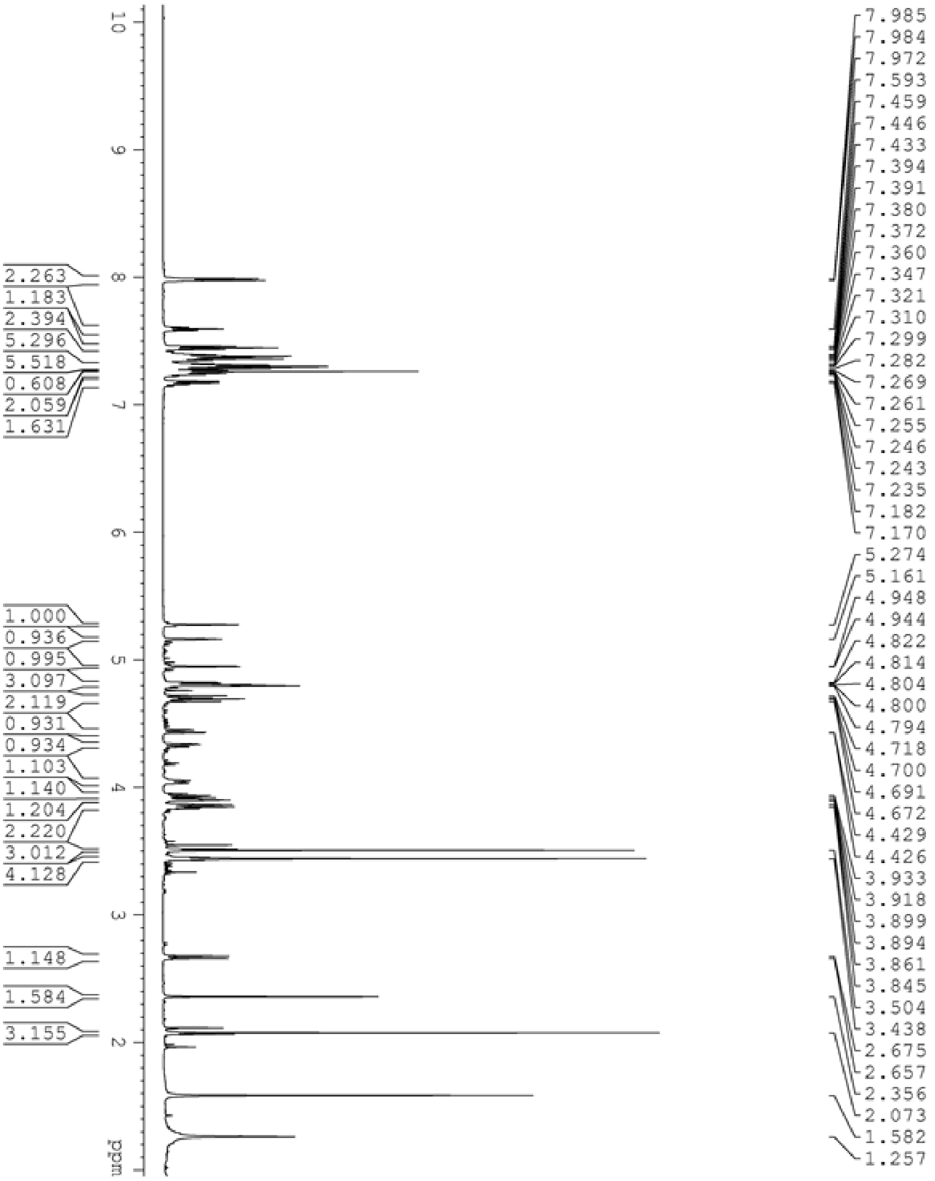

Supplementary Figure 29 | ^{1}H-NMR Spectrum of Compound 7a

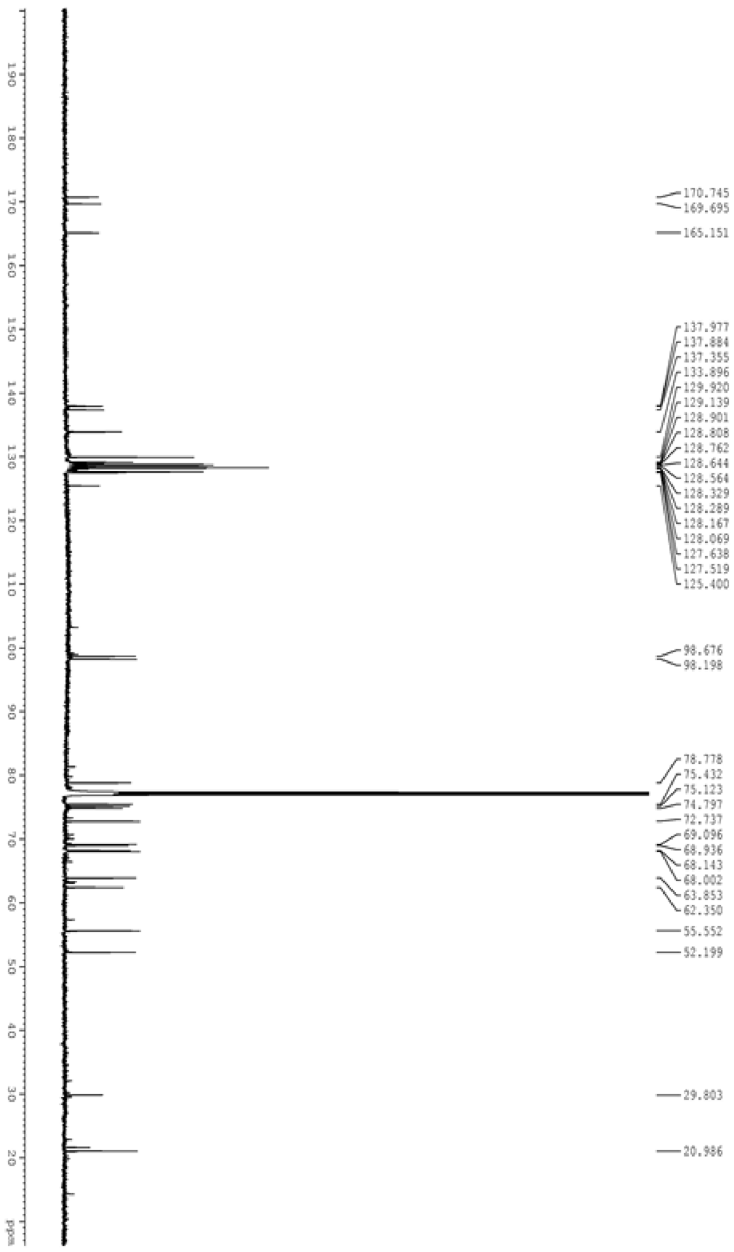

Supplementary Figure 30 | ^{13}C-NMR Spectrum of Compound 7a

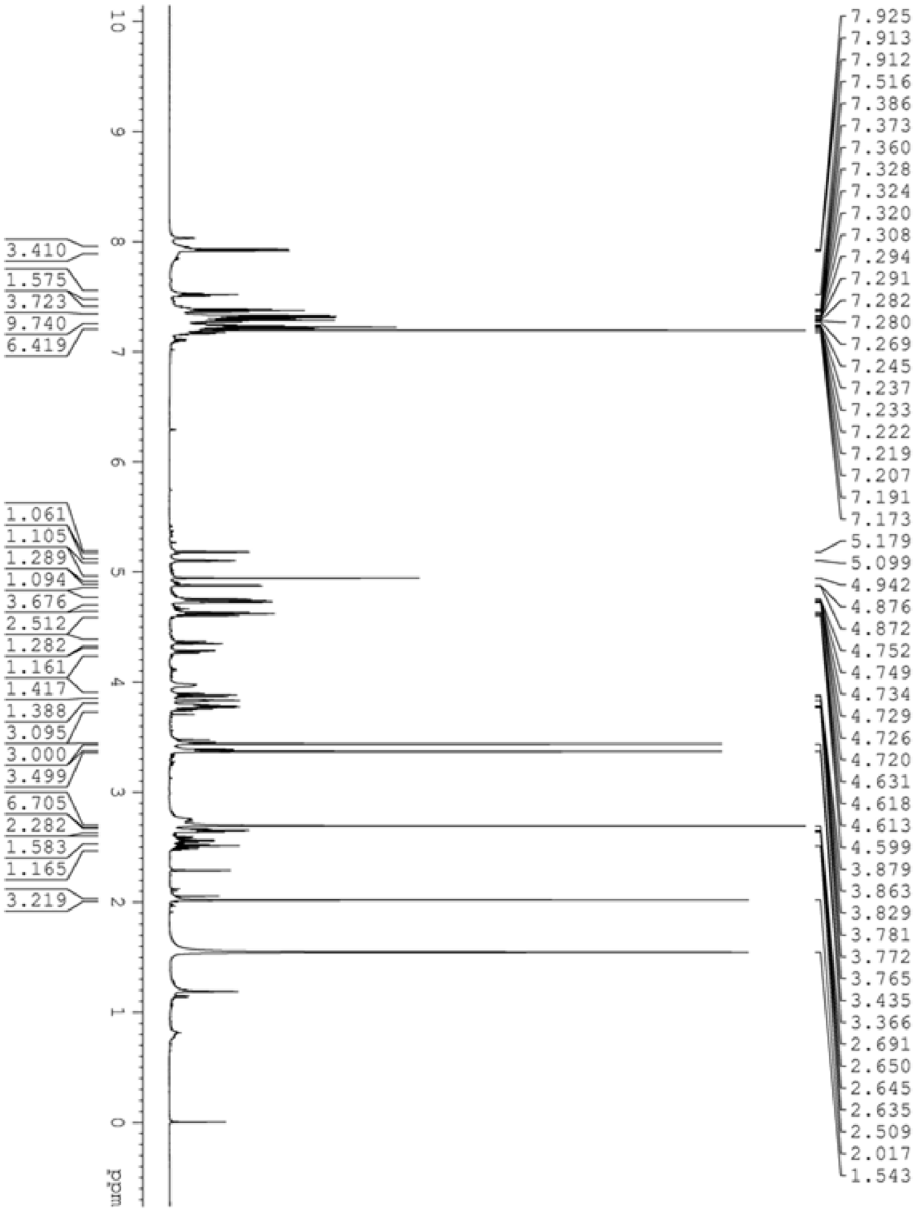

Supplementary Figure 31 | ^{1}H-NMR Spectrum of Compound 7b

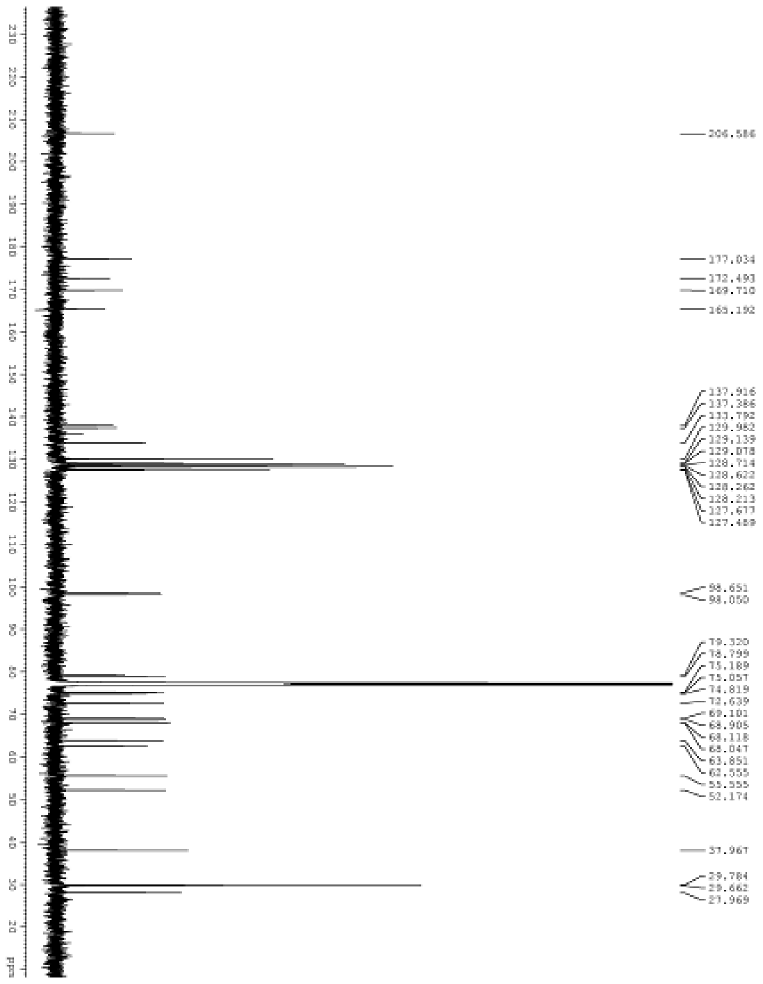

Supplementary Figure 32 | ^{13}C-NMR Spectrum of Compound 7b

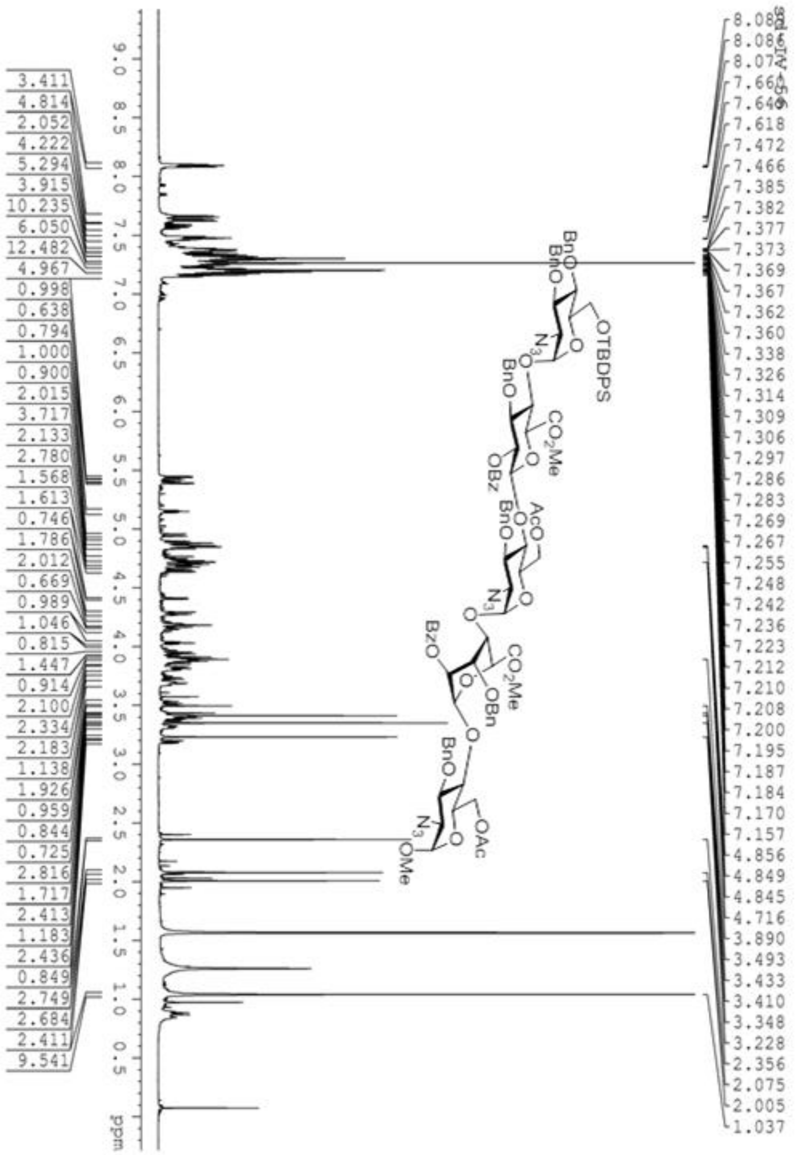

Supplementary Figure 33 | ^{1}H-NMR Spectrum of Compound 8a

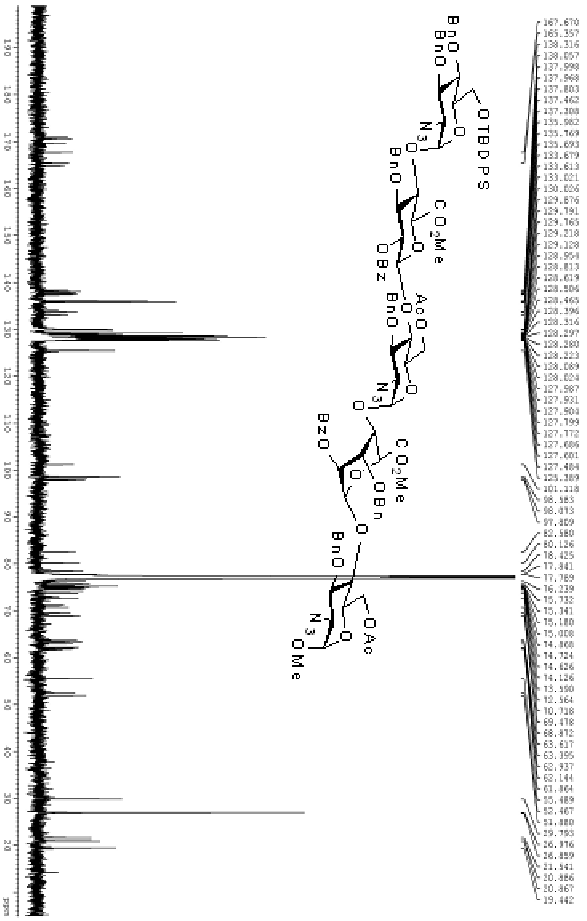

Supplementary Figure 34 | ^{13}C-NMR Spectrum of Compound 8a

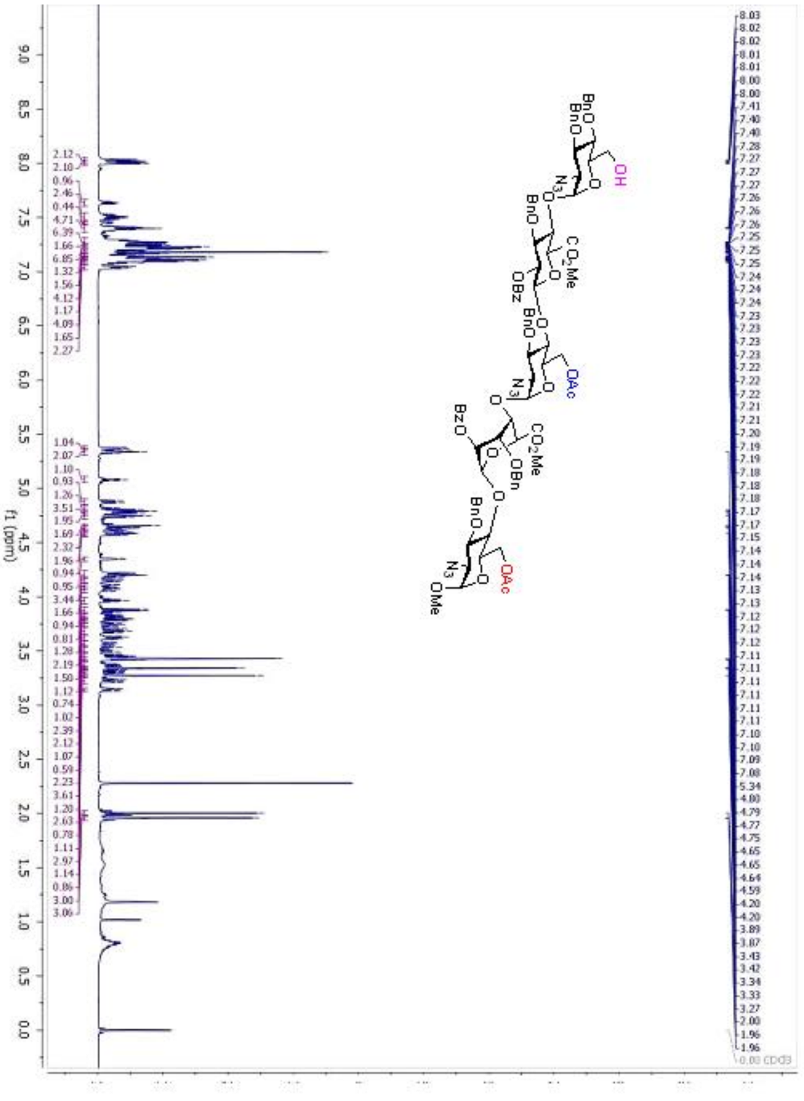

Supplementary Figure 35 | ^{1}H-NMR Spectrum of Compound 8b

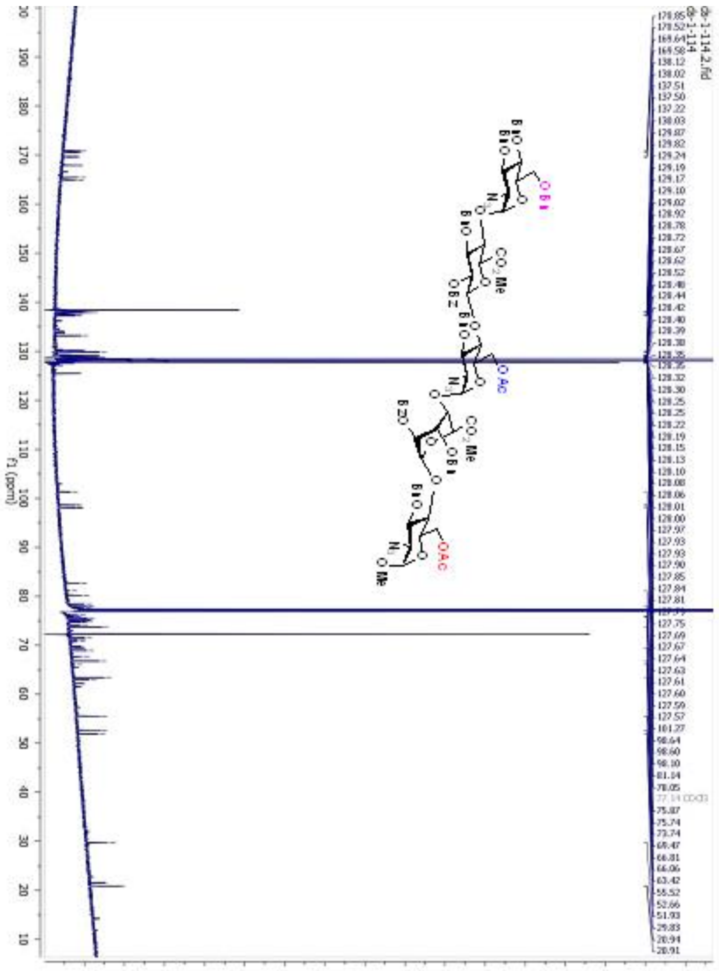

Supplementary Figure 36 | ^{13}C-NMR Spectrum of Compound 8b

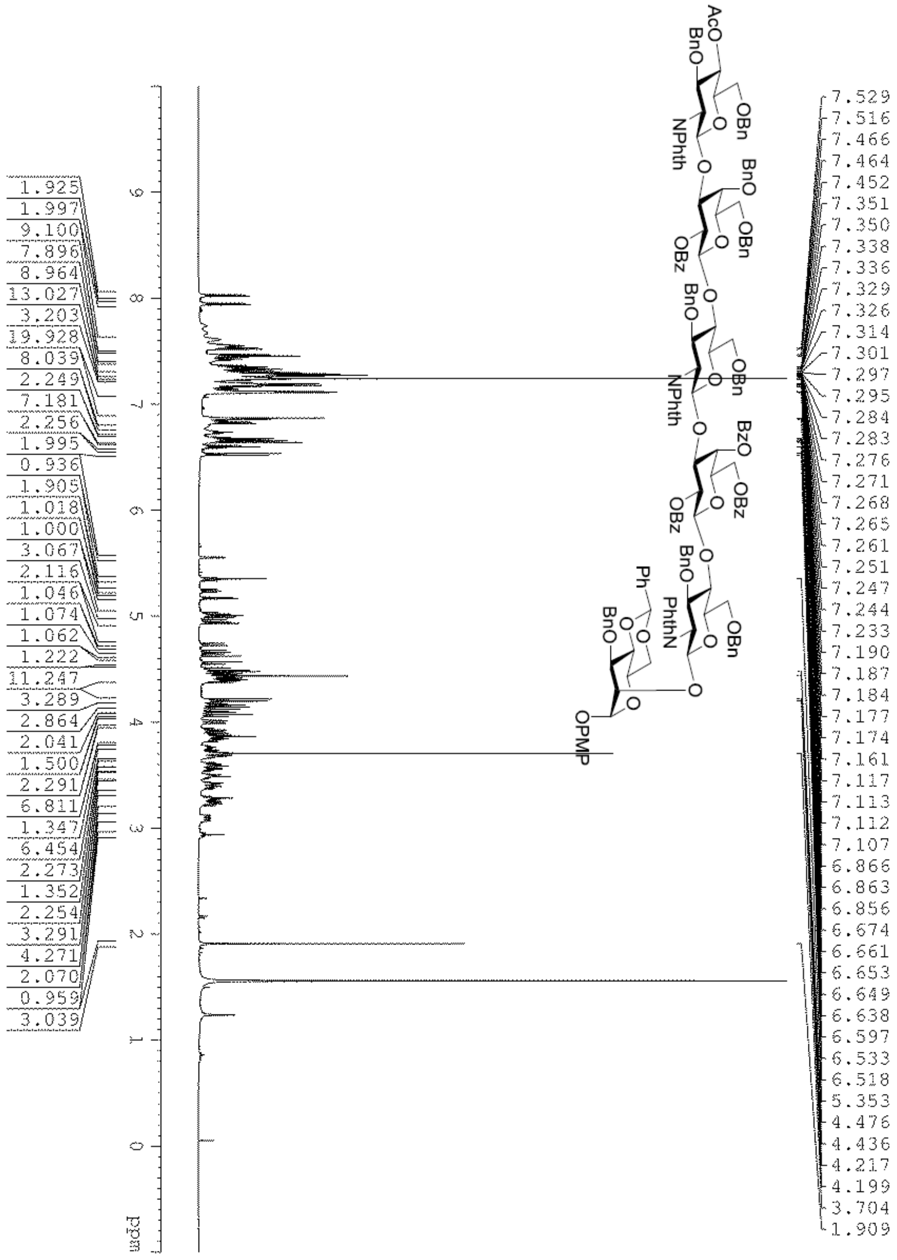

Supplementary Figure 37 | ^{1}H-NMR Spectrum of Compound 12

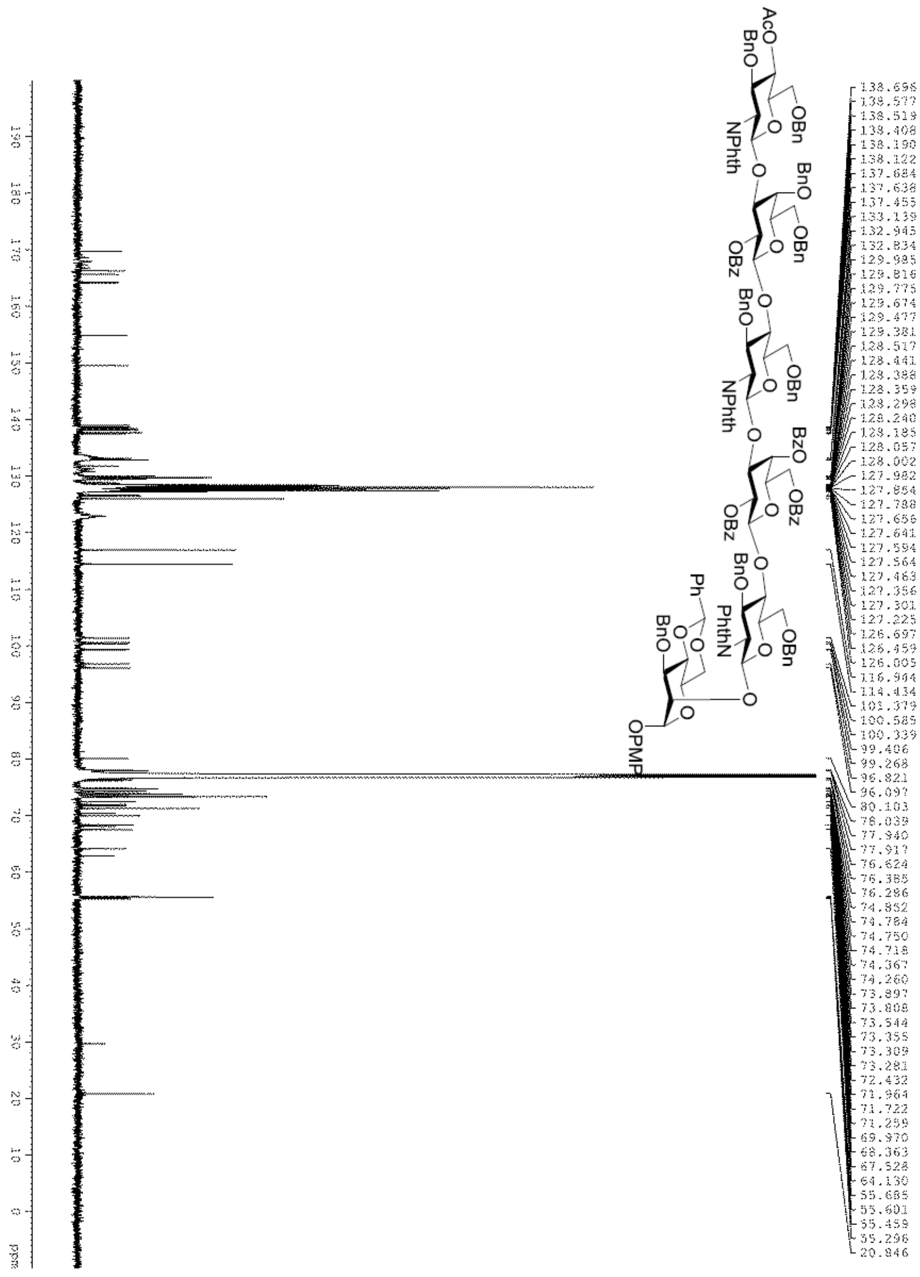

Supplementary Figure 38 | ^{13}C-NMR Spectrum of Compound 12

Supplementary References

1 Weishaupt, M. W. *et al.* Automated glycan assembly of a S. pneumoniae serotype 3 CPS antigen. *Beilstein J. Org. Chem.* **12**, 1440 (2016).

2 Hsu, C. H. *et al.* Highly Alpha-Selective Sialyl Phosphate Donors for Efficient Preparation of Natural Sialosides. *Chemistry-A European Journal* **16**, 1754-1760 (2010).

3 Huang, Y.-L. *et al.* Carbohydrate-based vaccines with a glycolipid adjuvant for breast cancer. *Proc. Natl. Acad. Sci. U. S. A.* **110**, 2517-2522 (2013).

4 Ting, C. Y., Lin, Y. W., Wu, C. Y. & Wong, C. H. Design of Disaccharide Modules for a Programmable One-Pot Synthesis of Building Blocks with LacNAc Repeating Units for Asymmetric N-Glycans. *Asia J. Org. Chem* **6**, 1800-1807 (2017).
