# Supplementary material for: Hierarchical and programmable one-pot synthesis of oligosaccharides
Source: Nat Commun. 2018 Dec 6;9:5202. doi: 10.1038/s41467-018-07618-8 (PMC6283847; doi:10.1038/s41467-018-07618-8)
Supplement: Supplementary file 3 — Description of Additional Supplementary Files [file 41467_2018_7618_MOESM3_ESM.pdf]

Description of Additional Supplementary Files

File Name: Supplementary Datasets 1-6
Description: Building Block Structures

File Name: Supplementary Dataset 7
Description: Building Block List

File Name: Supplementary Dataset 8
Description: Virtual Building Blocks with Predicted RRVs

File Name: Supplementary Dataset 9
Description: Experimental and Calculated Chemical Shifts

File Name: Supplementary Dataset 10
Description: Feature Selection Performance by 10-fold CV

File Name: Supplementary Dataset 11
Description: Performance of RRV Prediction by LOOCV

File Name: Supplementary Dataset 12
Description: Feature Selection for the Final RRV Predictor

File Name: Supplementary Dataset 13
Description: Rules in the Search Algorithm

File Name: Supplementary Dataset 14
Description: Tanimoto Similarity Matrix
