# Supplementary material for: Hierarchical and programmable one-pot synthesis of oligosaccharides
Source: Nat Commun. 2018 Dec 6;9:5202. doi: 10.1038/s41467-018-07618-8 (PMC6283847; doi:10.1038/s41467-018-07618-8)
Supplement: Supplementary file 3 [file 41467_2018_7618_MOESM4_ESM.docx]

1

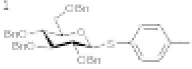

(2*R*,3*R*,4*S*,5*R*,6*S*)-3,4,5-tris(benzyloxy)-2-((benzyloxy)methyl)-6-(*p*-tolylthio)tetrahydro-2*H*-pyran

2

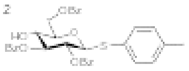

(2*S*,3*R*,4*S*,5*R*,6*R*)-6-((benzoyloxy)methyl)-5-hydroxy-2-(*p*-tolylthio)tetrahydro-2*H*-pyran-3,4-diyl dibenzoate

3

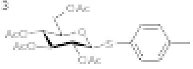

(2*R*,3*R*,4*S*,5*R*,6*S*)-2-(acetoxymethyl)-6-(*p*-tolylthio)tetrahydro-2*H*-pyran-3,4,5-triyl triacetate

4

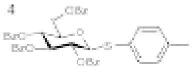

(2*R*,3*R*,4*S*,5*R*,6*S*)-2-((benzoyloxy)methyl)-6-(*p*-tolylthio)tetrahydro-2*H*-pyran-3,4,5-triyl tribenzoate

5

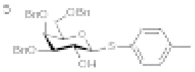

(2*S*,3*R*,4*R*,5*S*,6*R*)-4,5-bis(benzyloxy)-6-((benzyloxy)methyl)-2-(*p*-tolylthio)tetrahydro-2*H*-pyran-3-ol

6

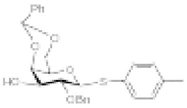

(2*S*,4a*R*,6*S*,7*R*,8*S*,8a*R*)-7-(benzyloxy)-2-phenyl-6-(*p*-tolylthio)hexahydropyrano[3,2-*d*][1,3]dioxin-8-ol

7

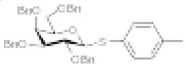

(2*R*,3*S*,4*S*,5*R*,6*S*)-3,4,5-tris(benzyloxy)-2-((benzyloxy)methyl)-6-(*p*-tolylthio)tetrahydro-2*H*-pyran

8

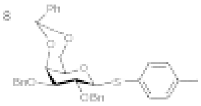

(2*S*,4a*R*,6*S*,7*R*,8*S*,8a*S*)-7,8-bis(benzyloxy)-2-phenyl-6-(*p*-tolylthio)hexahydropyrano[3,2-*d*][1,3]dioxine

9

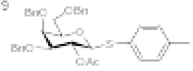

(2*S*,3*R*,4*S*,5*S*,6*R*)-4,5-bis(benzyloxy)-6-((benzyloxy)methyl)-2-(*p*-tolylthio)tetrahydro-2*H*-pyran-3-yl acetate

10

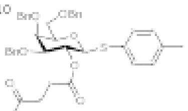

(2*S*,3*R*,4*S*,5*S*,6*R*)-4,5-bis(benzyloxy)-6-((benzyloxy)methyl)-2-(*p*-tolylthio)tetrahydro-2*H*-pyran-3-yl 4-oxopentanoate

11

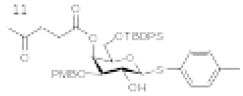

(2*R*,3*S*,4*R*,5*R*,6*S*)-2-(((*tert*-butyldiphenylsilyl)oxy)methyl)-5-hydroxy-4-((4-methoxybenzyl)oxy)-6-(*p*-tolylthio)tetrahydro-2*H*-pyran-3-yl 4-oxopentanoate

12

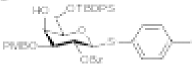

(2*S*,3*R*,4*S*,5*S*,6*R*)-6-(((*tert*-butyldiphenylsilyl)oxy)methyl)-5-hydroxy-4-((4-methoxybenzyl)oxy)-2-(*p*-tolylthio)tetrahydro-2*H*-pyran-3-yl benzoate

13

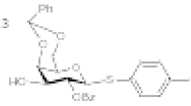

(2*S*,4a*R*,6*S*,7*R*,8*S*,8a*R*)-8-hydroxy-2-phenyl-6-(*p*-tolylthio)hexahydropyrano[3,2-*d*][1,3]dioxin-7-yl benzoate

14

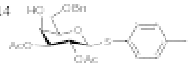

(2*S*,3*R*,4*S*,5*S*,6*R*)-6-((benzyloxy)methyl)-5-hydroxy-2-(*p*-tolylthio)tetrahydro-2*H*-pyran-3,4-diyl diacetate

15

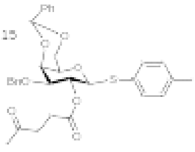

(2*S*,4a*R*,6*S*,7*R*,8*S*,8a*S*)-8-(benzyloxy)-2-phenyl-6-(*p*-tolylthio)hexahydropyrano[3,2-*d*][1,3]dioxin-7-yl 4-oxopentanoate

16

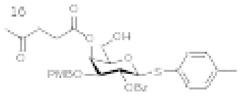

(2*S*,3*R*,4*S*,5*S*,6*R*)-6-(hydroxymethyl)-4-((4-methoxybenzyl)oxy)-5-((4-oxopentanoyl)oxy)-2-(*p*-tolylthio)tetrahydro-2*H*-pyran-3-yl benzoate

17

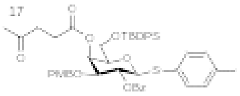

(2*S*,3*R*,4*S*,5*S*,6*R*)-6-(((*tert*-butyldiphenylsilyl)oxy)methyl)-4-((4-methoxybenzyl)oxy)-5-((4-oxopentanoyl)oxy)-2-(*p*-tolylthio)tetrahydro-2*H*-pyran-3-yl benzoate

18

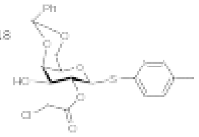

(2*S*,4a*R*,6*S*,7*R*,8*S*,8a*R*)-8-hydroxy-2-phenyl-6-(*p*-tolylthio)hexahydropyrano[3,2-*d*][1,3]dioxin-7-yl 2-chloroacetate

19

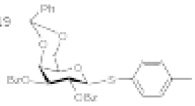

(2*S*,4a*R*,6*S*,7*R*,8*S*,8a*S*)-2-phenyl-6-(*p*-tolylthio)hexahydropyrano[3,2-*d*][1,3]dioxine-7,8-diyl dibenzoate

20

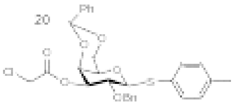

(2*S*,4a*R*,6*S*,7*R*,8*S*,8a*S*)-7-(benzyloxy)-2-phenyl-6-(*p*-tolylthio)hexahydropyrano[3,2-*d*][1,3]dioxin-8-yl 2-chloroacetate

21

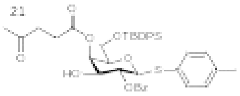

(2*S*,3*R*,4*S*,5*R*,6*R*)-6-(((*tert*-butyldiphenylsilyl)oxy)methyl)-4-hydroxy-5-((4-oxopentanoyl)oxy)-2-(*p*-tolylthio)tetrahydro-2*H*-pyran-3-yl benzoate

22

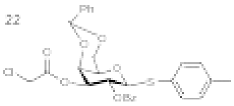

(2*S*,4a*R*,6*S*,7*R*,8*S*,8a*S*)-8-(2-chloroacetoxy)-2-phenyl-6-(*p*-tolylthio)hexahydropyrano[3,2-*d*][1,3]dioxin-7-yl benzoate

23

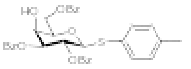

(2*S*,3*R*,4*S*,5*S*,6*R*)-6-((benzoyloxy)methyl)-5-hydroxy-2-(*p*-tolylthio)tetrahydro-2*H*-pyran-3,4-diyl dibenzoate

24

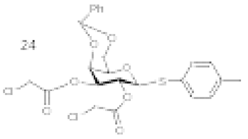

(2*S*,4a*R*,6*S*,7*R*,8*S*,8a*S*)-2-phenyl-6-(*p*-tolylthio)hexahydropyrano[3,2-*d*][1,3]dioxine-7,8-diyl bis(2-chloroacetate)

25

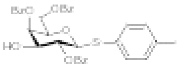

(2*R*,3*R*,4*S*,5*R*,6*S*)-2-((benzoyloxy)methyl)-4-hydroxy-6-(*p*-tolylthio)tetrahydro-2*H*-pyran-3,5-diyl dibenzoate

26

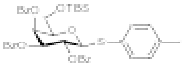

(2*R*,3*S*,4*S*,5*R*,6*S*)-2-(((*tert*-butyldimethylsilyl)oxy)methyl)-6-(*p*-tolylthio)tetrahydro-2*H*-pyran-3,4,5-triyl tribenzoate

27

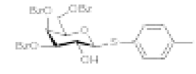

(2*R*,3*S*,4*R*,5*R*,6*S*)-2-((benzoyloxy)methyl)-5-hydroxy-6-(*p*-tolylthio)tetrahydro-2*H*-pyran-3,4-diyl dibenzoate

28

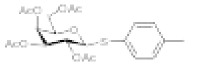

(2*R*,3*S*,4*S*,5*R*,6*S*)-2-(acetoxymethyl)-6-(*p*-tolylthio)tetrahydro-2*H*-pyran-3,4,5-triyl triacetate

29

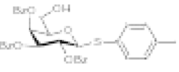

(2*R*,3*S*,4*S*,5*R*,6*S*)-2-(hydroxymethyl)-6-(*p*-tolylthio)tetrahydro-2*H*-pyran-3,4,5-triyl tribenzoate

30

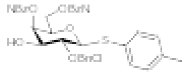

(2R,3R,4S,5R,6S)-5-((4-chlorobenzyl)oxy)-4-hydroxy-2-(((4-nitrobenzoyl)oxy)methyl)-6-(p-tolylthio)tetrahydro-2H-pyran-3-yl 4-nitrobenzoate

31

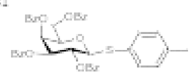

(2*R*,3*S*,4*S*,5*R*,6*S*)-2-((benzoyloxy)methyl)-6-(*p*-tolylthio)tetrahydro-2*H*-pyran-3,4,5-triyl tribenzoate

32

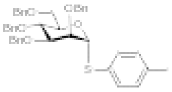

(2*R*,3*R*,4*S*,5*S*,6*R*)-3,4,5-tris(benzyloxy)-2-((benzyloxy)methyl)-6-(*p*-tolylthio)tetrahydro-2*H*-pyran

33

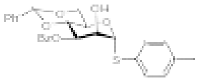

(2*R*,4a*R*,6*R*,7*S*,8*R*,8a*R*)-7-hydroxy-2-phenyl-6-(*p*-tolylthio)hexahydropyrano[3,2-*d*][1,3]dioxin-8-yl benzoate

34

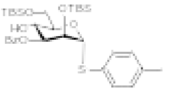

(2*R*,3*S*,4*S*,5*R*,6*R*)-3-((*tert*-butyldimethylsilyl)oxy)-6-(((*tert*-butyldimethylsilyl)oxy)methyl)-5-hydroxy-2-(*p*-tolylthio)tetrahydro-2*H*-pyran-4-yl benzoate

35

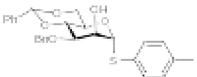

(2*R*,4a*R*,6*R*,7*S*,8*R*,8a*R*)-8-(benzyloxy)-2-phenyl-6-(*p*-tolylthio)hexahydropyrano[3,2-*d*][1,3]dioxin-7-ol

36

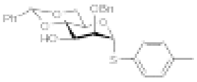

(2*R*,4a*R*,6*R*,7*S*,8*S*,8a*S*)-7-(benzyloxy)-2-phenyl-6-(*p*-tolylthio)hexahydropyrano[3,2-*d*][1,3]dioxin-8-ol

37

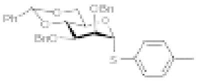

(2*R*,4a*R*,6*R*,7*S*,8*S*,8a*R*)-7,8-bis(benzyloxy)-2-phenyl-6-(*p*-tolylthio)hexahydropyrano[3,2-*d*][1,3]dioxine

38

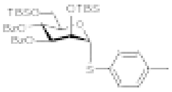

(2*R*,3*R*,4*S*,5*S*,6*R*)-5-((*tert*-butyldimethylsilyl)oxy)-2-(((*tert*-butyldimethylsilyl)oxy)methyl)-6-(*p*-tolylthio)tetrahydro-2*H*-pyran-3,4-diyl dibenzoate

39

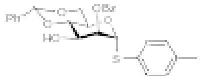

(2*R*,4a*R*,6*R*,7*S*,8*S*,8a*S*)-8-hydroxy-2-phenyl-6-(*p*-tolylthio)hexahydropyrano[3,2-*d*][1,3]dioxin-7-yl benzoate

40

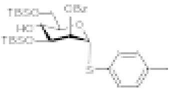

(2*R*,3*S*,4*S*,5*R*,6*R*)-4-((*tert*-butyldimethylsilyl)oxy)-6-(((*tert*-butyldimethylsilyl)oxy)methyl)-5-hydroxy-2-(*p*-tolylthio)tetrahydro-2*H*-pyran-3-yl benzoate

41

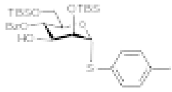

(2*R*,3*S*,4*S*,5*S*,6*R*)-5-((*tert*-butyldimethylsilyl)oxy)-2-(((*tert*-butyldimethylsilyl)oxy)methyl)-4-hydroxy-6-(*p*-tolylthio)tetrahydro-2*H*-pyran-3-yl benzoate

42

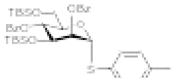

(2*R*,3*R*,4*S*,5*S*,6*R*)-4-((*tert*-butyldimethylsilyl)oxy)-2-(((*tert*-butyldimethylsilyl)oxy)methyl)-6-(*p*-tolylthio)tetrahydro-2*H*-pyran-3,5-diyl dibenzoate

43

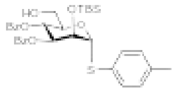

(2*R*,3*R*,4*S*,5*S*,6*R*)-5-((*tert*-butyldimethylsilyl)oxy)-2-(hydroxymethyl)-6-(*p*-tolylthio)tetrahydro-2*H*-pyran-3,4-diyl dibenzoate

44

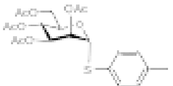

(2*R*,3*R*,4*S*,5*S*,6*R*)-2-(acetoxymethyl)-6-(*p*-tolylthio)tetrahydro-2*H*-pyran-3,4,5-triyl triacetate

45

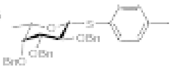

(2*S*,3*R*,4*R*,5*S*,6*R*)-3,4,5-tris(benzyloxy)-2-methyl-6-(*p*-tolylthio)tetrahydro-2*H*-pyran

46

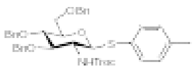

2,2,2-trichloroethyl ((2*S*,3*R*,4*R*,5*S*,6*R*)-4,5-bis(benzyloxy)-6-((benzyloxy)methyl)-2-(*p*-tolylthio)tetrahydro-2*H*-pyran-3-yl)carbamate

47

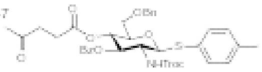

(2*R*,3*S*,4*R*,5*R*,6*S*)-2-((benzyloxy)methyl)-3-((4-oxopentanoyl)oxy)-6-(*p*-tolylthio)-5-(((2,2,2-trichloroethoxy)carbonyl)amino)tetrahydro-2*H*-pyran-4-yl benzoate

48

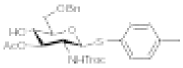

(2*R*,3*S*,4*R*,5*R*,6*S*)-2-((benzyloxy)methyl)-3-hydroxy-6-(*p*-tolylthio)-5-(((2,2,2-trichloroethoxy)carbonyl)amino)tetrahydro-2*H*-pyran-4-yl acetate

49

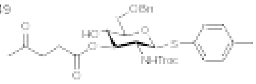

(2*R*,3*S*,4*R*,5*R*,6*S*)-2-((benzyloxy)methyl)-3-hydroxy-6-(*p*-tolylthio)-5-(((2,2,2-trichloroethoxy)carbonyl)amino)tetrahydro-2*H*-pyran-4-yl 4-oxopentanoate

50

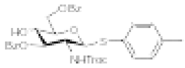

((2*R*,3*S*,4*R*,5*R*,6*S*)-4-(benzoyloxy)-3-hydroxy-6-(*p*-tolylthio)-5-(((2,2,2-trichloroethoxy)carbonyl)amino)tetrahydro-2*H*-pyran-2-yl)methyl benzoate

51

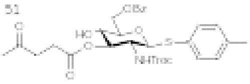

((2*R*,3*S*,4*R*,5*R*,6*S*)-3-hydroxy-4-((4-oxopentanoyl)oxy)-6-(*p*-tolylthio)-5-(((2,2,2-trichloroethoxy)carbonyl)amino)tetrahydro-2*H*-pyran-2-yl)methyl benzoate

52

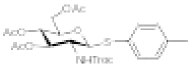

(2*R*,3*S*,4*R*,5*R*,6*S*)-2-(acetoxymethyl)-6-(*p*-tolylthio)-5-(((2,2,2-trichloroethoxy)carbonyl)amino)tetrahydro-2*H*-pyran-3,4-diyl diacetate

53

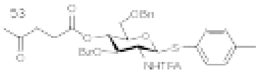

(2*R*,3*S*,4*R*,5*R*,6*S*)-2-((benzyloxy)methyl)-3-((4-oxopentanoyl)oxy)-6-(*p*-tolylthio)-5-(2,2,2-trifluoroacetamido)tetrahydro-2*H*-pyran-4-yl benzoate

54

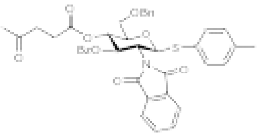

(2*R*,3*S*,4*R*,5*R*,6*S*)-2-((benzyloxy)methyl)-5-(1,3-dioxoisoindolin-2-yl)-3-((4-oxopentanoyl)oxy)-6-(*p*-tolylthio)tetrahydro-2*H*-pyran-4-yl benzoate

55

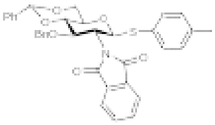

2-((2*R*,4a*R*,6*S*,7*R*,8*R*,8a*S*)-8-(benzyloxy)-2-phenyl-6-(*p*-tolylthio)hexahydropyrano[3,2-*d*][1,3]dioxin-7-yl)isoindoline-1,3-dione

56

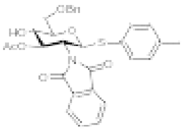

(2*R*,3*S*,4*R*,5*R*,6*S*)-2-((benzyloxy)methyl)-5-(1,3-dioxoisoindolin-2-yl)-3-hydroxy-6-(*p*-tolylthio)tetrahydro-2*H*-pyran-4-yl acetate

57

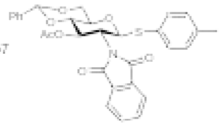

(2*R*,4a*R*,6*S*,7*R*,8*R*,8a*S*)-7-(1,3-dioxoisoindolin-2-yl)-2-phenyl-6-(*p*-tolylthio)hexahydropyrano[3,2-*d*][1,3]dioxin-8-yl acetate

58

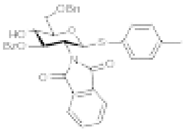

(2*R*,3*S*,4*R*,5*R*,6*S*)-2-((benzyloxy)methyl)-5-(1,3-dioxoisoindolin-2-yl)-3-hydroxy-6-(*p*-tolylthio)tetrahydro-2*H*-pyran-4-yl benzoate

59

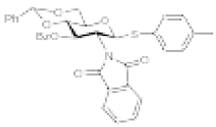

(2*R*,4a*R*,6*S*,7*R*,8*R*,8a*S*)-7-(1,3-dioxoisoindolin-2-yl)-2-phenyl-6-(*p*-tolylthio)hexahydropyrano[3,2-*d*][1,3]dioxin-8-yl benzoate

60

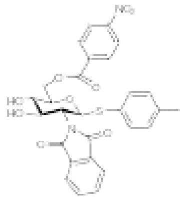

((2*R*,3*S*,4*R*,5*R*,6*S*)-5-(1,3-dioxoisoindolin-2-yl)-3,4-dihydroxy-6-(*p*-tolylthio)tetrahydro-2*H*-pyran-2-yl)methyl 4-nitrobenzoate

61

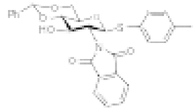

2-((2*R*,4a*R*,6*S*,7*R*,8*R*,8a*S*)-8-hydroxy-2-phenyl-6-(*p*-tolylthio)hexahydropyrano[3,2-*d*][1,3]dioxin-7-yl)isoindoline-1,3-dione

62

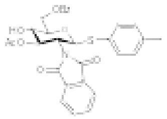

((2*R*,3*S*,4*R*,5*R*,6*S*)-4-acetoxy-5-(1,3-dioxoisoindolin-2-yl)-3-hydroxy-6-(*p*-tolylthio)tetrahydro-2*H*-pyran-2-yl)methyl benzoate

63

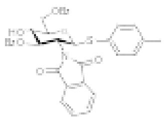

((2*R*,3*S*,4*R*,5*R*,6*S*)-4-(benzoyloxy)-5-(1,3-dioxoisoindolin-2-yl)-3-hydroxy-6-(*p*-tolylthio)tetrahydro-2*H*-pyran-2-yl)methyl benzoate

64

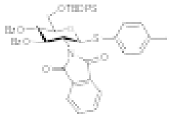

(2*R*,3*S*,4*R*,5*R*,6*S*)-2-(((*tert*-butyldiphenylsilyl)oxy)methyl)-5-(1,3-dioxoisoindolin-2-yl)-6-(*p*-tolylthio)tetrahydro-2*H*-pyran-3,4-diyl dibenzoate

65

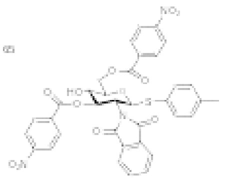

((2R,3S,4R,5R,6S)-5-(1,3-dioxoisoindolin-2-yl)-3-hydroxy-4-((4-nitrobenzoyl)oxy)-6-(p-tolylthio)tetrahydro-2H-pyran-2-yl)methyl 4-nitrobenzoate

66

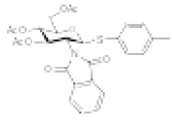

(2*R*,3*S*,4*R*,5*R*,6*S*)-2-(acetoxymethyl)-5-(1,3-dioxoisoindolin-2-yl)-6-(*p*-tolylthio)tetrahydro-2*H*-pyran-3,4-diyl diacetate

67

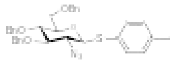

(2*S*,3*R*,4*R*,5*S*,6*R*)-3-azido-4,5-bis(benzyloxy)-6-((benzyloxy)methyl)-2-(*p*-tolylthio)tetrahydro-2*H*-pyran

68

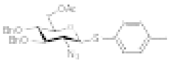

((2*R*,3*S*,4*R*,5*R*,6*S*)-5-azido-3,4-bis(benzyloxy)-6-(*p*-tolylthio)tetrahydro-2*H*-pyran-2-yl)methyl acetate

69

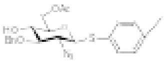

((2*R*,3*S*,4*R*,5*R*,6*S*)-5-azido-4-(benzyloxy)-3-hydroxy-6-(*p*-tolylthio)tetrahydro-2*H*-pyran-2-yl)methyl acetate

70

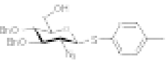

((2*R*,3*S*,4*R*,5*R*,6*S*)-5-azido-3,4-bis(benzyloxy)-6-(*p*-tolylthio)tetrahydro-2*H*-pyran-2-yl)methanol

71

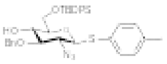

(2*R*,3*S*,4*R*,5*R*,6*S*)-5-azido-4-(benzyloxy)-2-(((*tert*-butyldiphenylsilyl)oxy)methyl)-6-(*p*-tolylthio)tetrahydro-2*H*-pyran-3-ol

72

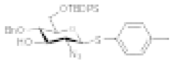

(2*S*,3*R*,4*R*,5*S*,6*R*)-3-azido-5-(benzyloxy)-6-(((*tert*-butyldiphenylsilyl)oxy)methyl)-2-(*p*-tolylthio)tetrahydro-2*H*-pyran-4-ol

73

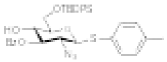

(2*S*,3*R*,4*R*,5*S*,6*R*)-3-azido-6-(((*tert*-butyldiphenylsilyl)oxy)methyl)-5-hydroxy-2-(*p*-tolylthio)tetrahydro-2*H*-pyran-4-yl benzoate

74

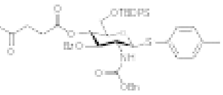

(2*S*,3*R*,4*R*,5*S*,6*R*)-3-(((benzyloxy)carbonyl)amino)-6-(((*tert*-butyldiphenylsilyl)oxy)methyl)-5-((4-oxopentanoyl)oxy)-2-(*p*-tolylthio)tetrahydro-2*H*-pyran-4-yl benzoate

75

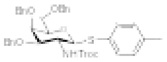

2,2,2-trichloroethyl ((2*S*,3*R*,4*R*,5*R*,6*R*)-4,5-bis(benzyloxy)-6-((benzyloxy)methyl)-2-(*p*-tolylthio)tetrahydro-2*H*-pyran-3-yl)carbamate

76

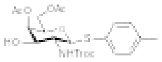

((2*R*,3*R*,4*R*,5*R*,6*S*)-3-acetoxy-4-hydroxy-6-(*p*-tolylthio)-5-(((2,2,2-trichloroethoxy)carbonyl)amino)tetrahydro-2*H*-pyran-2-yl)methyl acetate

77

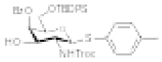

(2*R*,3*R*,4*R*,5*R*,6*S*)-2-(((*tert*-butyldiphenylsilyl)oxy)methyl)-4-hydroxy-6-(*p*-tolylthio)-5-(((2,2,2-trichloroethoxy)carbonyl)amino)tetrahydro-2*H*-pyran-3-yl benzoate

78

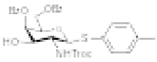

((2*R*,3*R*,4*R*,5*R*,6*S*)-3-(benzoyloxy)-4-hydroxy-6-(*p*-tolylthio)-5-(((2,2,2-trichloroethoxy)carbonyl)amino)tetrahydro-2*H*-pyran-2-yl)methyl benzoate

79

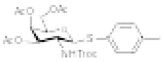

(2*R*,3*R*,4*R*,5*R*,6*S*)-2-(acetoxymethyl)-6-(*p*-tolylthio)-5-(((2,2,2-trichloroethoxy)carbonyl)amino)tetrahydro-2*H*-pyran-3,4-diyl diacetate

80

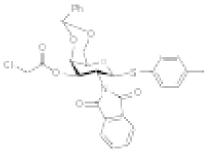

(2*S*,4a*R*,6*S*,7*R*,8*R*,8a*R*)-7-(1,3-dioxoisoindolin-2-yl)-2-phenyl-6-(*p*-tolylthio)hexahydropyrano[3,2-*d*][1,3]dioxin-8-yl 2-chloroacetate

81

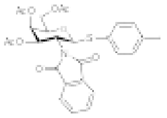

(2*R*,3*R*,4*R*,5*R*,6*S*)-2-(acetoxymethyl)-5-(1,3-dioxoisoindolin-2-yl)-6-(*p*-tolylthio)tetrahydro-2*H*-pyran-3,4-diyl diacetate

82

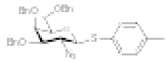

(2*S*,3*R*,4*R*,5*R*,6*R*)-3-azido-4,5-bis(benzyloxy)-6-((benzyloxy)methyl)-2-(*p*-tolylthio)tetrahydro-2*H*-pyran

83

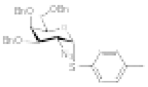

(2*R*,3*R*,4*R*,5*R*,6*R*)-3-azido-4,5-bis(benzyloxy)-6-((benzyloxy)methyl)-2-(*p*-tolylthio)tetrahydro-2*H*-pyran

84

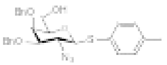

((2*R*,3*R*,4*R*,5*R*,6*S*)-5-azido-3,4-bis(benzyloxy)-6-(*p*-tolylthio)tetrahydro-2*H*-pyran-2-yl)methanol

85

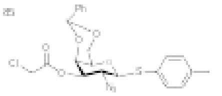

(2*S*,4a*R*,6*S*,7*R*,8*R*,8a*R*)-7-azido-2-phenyl-6-(*p*-tolylthio)hexahydropyrano[3,2-*d*][1,3]dioxin-8-yl 2-chloroacetate

86

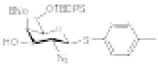

((2*R*,3*R*,4*R*,5*R*,6*S*)-5-azido-2-(((*tert*-butyldiphenylsilyl)oxy)methyl)-4-hydroxy-6-(*p*-tolylthio)tetrahydro-2*H*-pyran-3-yl)(λ1-boranyl)nobelium

87

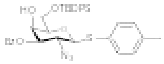

(2*S*,3*R*,4*R*,5*R*,6*R*)-3-azido-6-(((*tert*-butyldiphenylsilyl)oxy)methyl)-5-hydroxy-2-(*p*-tolylthio)tetrahydro-2*H*-pyran-4-yl benzoate

88

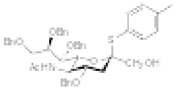

N-((2*R*,3*R*,4*S*,6*R*)-4-(benzyloxy)-6-(hydroxymethyl)-6-(*p*-tolylthio)-2-((1*S*,2*R*)-1,2,3-tris(benzyloxy)propyl)tetrahydro-2*H*-pyran-3-yl)acetamide

89

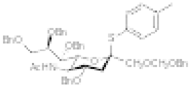

N-((2*R*,3*R*,4*S*,6*R*)-4-(benzyloxy)-6-(((benzyloxy)methoxy)methyl)-6-(*p*-tolylthio)-2-((1*S*,2*R*)-1,2,3-tris(benzyloxy)propyl)tetrahydro-2*H*-pyran-3-yl)acetamide

90

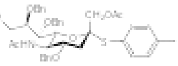

((2*S*,4*S*,5*R*,6*R*)-5-acetamido-4-(benzyloxy)-2-(*p*-tolylthio)-6-((1*S*,2*R*)-1,2,3-tris(benzyloxy)propyl)tetrahydro-2*H*-pyran-2-yl)methyl acetate

91

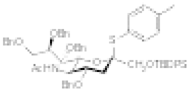

N-((2R,3R,4S,6R)-4-(benzyloxy)-6-(((tert-butyldiphenylsilyl)oxy)methyl)-6-(p-tolylthio)-2-((1S,2R)-1,2,3-tris(benzyloxy)propyl)tetrahydro-2H-pyran-3-yl)acetamide

92

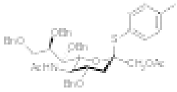

((2R,4S,5R,6R)-5-acetamido-4-(benzyloxy)-2-(p-tolylthio)-6-((1S,2R)-1,2,3-tris(benzyloxy)propyl)tetrahydro-2H-pyran-2-yl)methyl acetate

93

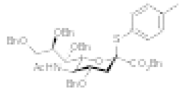

benzyl (2R,4S,5R,6R)-5-acetamido-4-(benzyloxy)-2-(p-tolylthio)-6-((1S,2R)-1,2,3-tris(benzyloxy)propyl)tetrahydro-2H-pyran-2-carboxylate

94

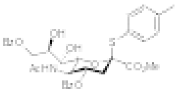

methyl (2R,4S,5R,6R)-5-acetamido-4-(benzoyloxy)-6-((1R,2R)-3-(benzoyloxy)-1,2-dihydroxypropyl)-2-(p-tolylthio)tetrahydro-2H-pyran-2-carboxylate

95

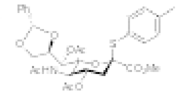

methyl (2R,4S,5R,6R)-5-acetamido-4-acetoxy-6-((S)-acetoxy((2S,4R)-2-phenyl-1,3-dioxolan-4-yl)methyl)-2-(p-tolylthio)tetrahydro-2H-pyran-2-carboxylate

96

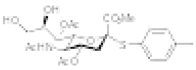

methyl (2S,4S,5R,6R)-5-acetamido-4-acetoxy-6-((1R,2R)-1-acetoxy-2,3-dihydroxypropyl)-2-(p-tolylthio)tetrahydro-2H-pyran-2-carboxylate

97

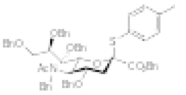

benzyl (2R,4S,5R,6R)-5-(N-benzylacetamido)-4-(benzyloxy)-2-(p-tolylthio)-6-((1S,2R)-1,2,3-tris(benzyloxy)propyl)tetrahydro-2H-pyran-2-carboxylate

98

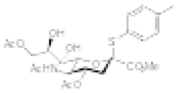

methyl (2R,4S,5R,6R)-5-acetamido-4-acetoxy-6-((1R,2R)-3-acetoxy-1,2-dihydroxypropyl)-2-(p-tolylthio)tetrahydro-2H-pyran-2-carboxylate

99

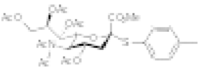

(1S,2R)-1-((2R,3R,4S,6S)-4-acetoxy-3-(N-acetylacetamido)-6-(methoxycarbonyl)-6-(p-tolylthio)tetrahydro-2H-pyran-2-yl)propane-1,2,3-triyl triacetate

100

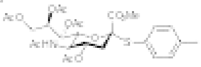

(1S,2R)-1-((2R,3R,4S,6S)-3-acetamido-4-acetoxy-6-(methoxycarbonyl)-6-(p-tolylthio)tetrahydro-2H-pyran-2-yl)propane-1,2,3-triyl triacetate

101

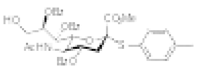

(1S,2R)-1-((2R,3R,4S,6S)-3-acetamido-4-(benzoyloxy)-6-(methoxycarbonyl)-6-(p-tolylthio)tetrahydro-2H-pyran-2-yl)-3-hydroxypropane-1,2-diyl dibenzoate

102

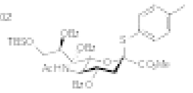

(1S,2R)-1-((2R,3R,4S,6R)-3-acetamido-4-(benzoyloxy)-6-(methoxycarbonyl)-6-(p-tolylthio)tetrahydro-2H-pyran-2-yl)-3-((tert-butyldimethylsilyl)oxy)propane-1,2-diyl dibenzoate

103

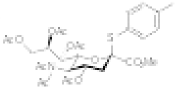

(1S,2R)-1-((2R,3R,4S,6R)-4-acetoxy-3-(N-acetylacetamido)-6-(methoxycarbonyl)-6-(p-tolylthio)tetrahydro-2H-pyran-2-yl)propane-1,2,3-triyl triacetate

104

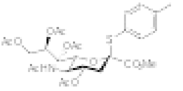

(1S,2R)-1-((2R,3R,4S,6R)-3-acetamido-4-acetoxy-6-(methoxycarbonyl)-6-(p-tolylthio)tetrahydro-2H-pyran-2-yl)propane-1,2,3-triyl triacetate

105

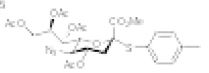

(1S,2R)-1-((2R,3R,4S,6S)-4-acetoxy-3-azido-6-(methoxycarbonyl)-6-(p-tolylthio)tetrahydro-2H-pyran-2-yl)propane-1,2,3-triyl triacetate

106

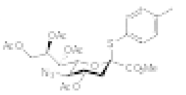

(1S,2R)-1-((2R,3R,4S,6R)-4-acetoxy-3-azido-6-(methoxycarbonyl)-6-(p-tolylthio)tetrahydro-2H-pyran-2-yl)propane-1,2,3-triyl triacetate

107

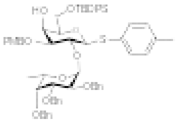

(2R,3S,4S,5R,6S)-2-(((tert-butyldiphenylsilyl)oxy)methyl)-4-((4-methoxybenzyl)oxy)-6-(p-tolylthio)-5-(((2S,3S,4R,5R,6S)-3,4,5-tris(benzyloxy)-6-methyltetrahydro-2H-pyran-2-yl)oxy)tetrahydro-2H-pyran-3-ol

108

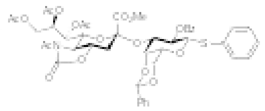

(1S,2R)-1-((3aR,4R,6S,7aS)-3-acetyl-6-(((2S,6R,7S,8R)-7-(benzoyloxy)-2-phenyl-6-(phenylthio)hexahydropyrano[3,2-d][1,3]dioxin-8-yl)oxy)-6-(methoxycarbonyl)-2-oxohexahydro-4H-pyrano[3,4-d]oxazol-4-yl)propane-1,2,3-triyl triacetate

109

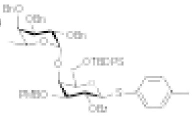

(2S,3R,4S,5S,6R)-6-(((tert-butyldiphenylsilyl)oxy)methyl)-4-((4-methoxybenzyl)oxy)-2-(p-tolylthio)-5-(((2R,3R,4S,5S,6R)-3,4,5-tris(benzyloxy)-6-methyltetrahydro-2H-pyran-2-yl)oxy)tetrahydro-2H-pyran-3-yl benzoate

110

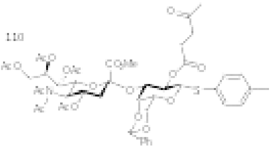

(1S,2R)-1-((2R,3R,4S,6S)-4-acetoxy-3-(N-acetylacetamido)-6-(methoxycarbonyl)-6-(((2S,6R,7S,8R)-7-((4-oxopentanoyl)oxy)-2-phenyl-6-(p-tolylthio)hexahydropyrano[3,2-d][1,3]dioxin-8-yl)oxy)tetrahydro-2H-pyran-2-yl)propane-1,2,3-triyl triacetate

111

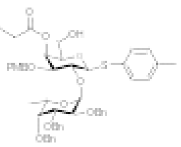

(2R,3S,4S,5R,6S)-2-(hydroxymethyl)-4-((4-methoxybenzyl)oxy)-6-(p-tolylthio)-5-(((2S,3S,4R,5R,6S)-3,4,5-tris(benzyloxy)-6-methyltetrahydro-2H-pyran-2-yl)oxy)tetrahydro-2H-pyran-3-yl 4-oxopentanoate

112

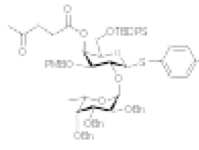

(2R,3S,4S,5R,6S)-2-(((tert-butyldiphenylsilyl)oxy)methyl)-4-((4-methoxybenzyl)oxy)-6-(p-tolylthio)-5-(((2S,3S,4R,5R,6S)-3,4,5-tris(benzyloxy)-6-methyltetrahydro-2H-pyran-2-yl)oxy)tetrahydro-2H-pyran-3-yl 4-oxopentanoate

113

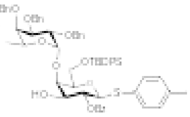

(2S,3R,4S,5R,6R)-6-(((tert-butyldiphenylsilyl)oxy)methyl)-4-hydroxy-2-(p-tolylthio)-5-(((2R,3R,4S,5S,6R)-3,4,5-tris(benzyloxy)-6-methyltetrahydro-2H-pyran-2-yl)oxy)tetrahydro-2H-pyran-3-yl benzoate

114

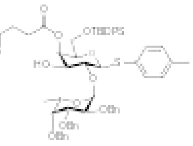

(2R,3R,4S,5R,6S)-2-(((tert-butyldiphenylsilyl)oxy)methyl)-4-hydroxy-6-(p-tolylthio)-5-(((2S,3S,4R,5R,6S)-3,4,5-tris(benzyloxy)-6-methyltetrahydro-2H-pyran-2-yl)oxy)tetrahydro-2H-pyran-3-yl 4-oxopentanoate

115

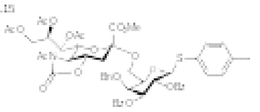

(1S,2R)-1-((3aR,4R,6R,7aS)-3-acetyl-6-(((2R,3S,4S,5R,6S)-4,5-bis(benzyloxy)-3-(benzoyloxy)-6-(p-tolylthio)tetrahydro-2H-pyran-2-yl)methoxy)-6-(methoxycarbonyl)-2-oxohexahydro-4H-pyrano[3,4-d]oxazol-4-yl)propane-1,2,3-triyl triacetate

116

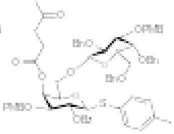

(2S,3R,4S,5S,6R)-6-((((2R,3R,4S,5S,6R)-3,5-bis(benzyloxy)-6-((benzyloxy)methyl)-4-((4-methoxybenzyl)oxy)tetrahydro-2H-pyran-2-yl)oxy)methyl)-4-((4-methoxybenzyl)oxy)-5-((4-oxopentanoyl)oxy)-2-(p-tolylthio)tetrahydro-2H-pyran-3-yl benzoate

117

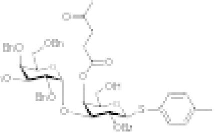

(2S,3R,4S,5S,6R)-6-(hydroxymethyl)-5-((4-oxopentanoyl)oxy)-2-(p-tolylthio)-4-(((2R,3R,4S,5S,6R)-3,4,5-tris(benzyloxy)-6-((benzyloxy)methyl)tetrahydro-2H-pyran-2-yl)oxy)tetrahydro-2H-pyran-3-yl benzoate

118

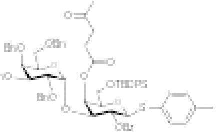

(2S,3R,4S,5S,6R)-6-(((tert-butyldiphenylsilyl)oxy)methyl)-5-((4-oxopentanoyl)oxy)-2-(p-tolylthio)-4-(((2R,3R,4S,5S,6R)-3,4,5-tris(benzyloxy)-6-((benzyloxy)methyl)tetrahydro-2H-pyran-2-yl)oxy)tetrahydro-2H-pyran-3-yl benzoate

119

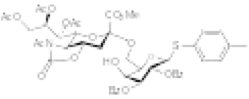

(1S,2R)-1-((3aR,4R,6R,7aS)-3-acetyl-6-(((2R,3S,4S,5R,6S)-4,5-bis(benzyloxy)-3-hydroxy-6-(p-tolylthio)tetrahydro-2H-pyran-2-yl)methoxy)-6-(methoxycarbonyl)-2-oxohexahydro-4H-pyrano[3,4-d]oxazol-4-yl)propane-1,2,3-triyl triacetate

120

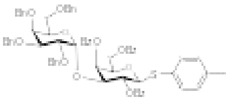

(2R,3S,4S,5R,6S)-2-((benzyloxy)methyl)-6-(p-tolylthio)-4-(((2R,3R,4S,5S,6R)-3,4,5-tris(benzyloxy)-6-((benzyloxy)methyl)tetrahydro-2H-pyran-2-yl)oxy)tetrahydro-2H-pyran-3,5-diyl dibenzoate

121

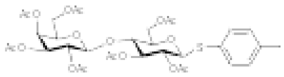

(2R,3S,4S,5R,6S)-2-(acetoxymethyl)-6-(((2R,3R,4S,5R,6S)-4,5-diacetoxy-2-(acetoxymethyl)-6-(p-tolylthio)tetrahydro-2H-pyran-3-yl)oxy)tetrahydro-2H-pyran-3,4,5-triyl triacetate

122

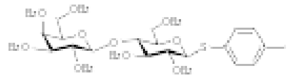

(2R,3S,4S,5R,6S)-2-((benzoyloxy)methyl)-6-(((2R,3R,4S,5R,6S)-4,5-bis(benzoyloxy)-2-((benzoyloxy)methyl)-6-(p-tolylthio)tetrahydro-2H-pyran-3-yl)oxy)tetrahydro-2H-pyran-3,4,5-triyl tribenzoate

123

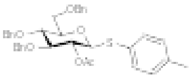

(2S,3S,4S,5R)-2-(p-tolylthio)-4,5-bis(benzyloxy)-6-((benzyloxy)methyl)-tetrahydro-2H-pyran-3-yl acetate

124

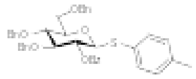

(2S,3S,4S,5R)-2-(p-tolylthio)-4,5-bis(benzyloxy)-6-((benzyloxy)methyl)-tetrahydro-2H-pyran-3-ylbenzoate

125

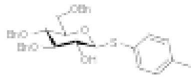

(2S,3S,4R,5R)-2-(p-tolylthio)-4,5-bis(benzyloxy)-6-((benzyloxy)methyl)-tetrahydro-2H-pyran-3-ol

126

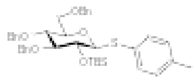

((2S,3S,4S,5R)-2-(p-tolylthio)-4,5-bis(benzyloxy)-6-((benzyloxy)methyl)-tetrahydro-2H-pyran-3-yloxy)(tert-butyl)dimethylsilane

127

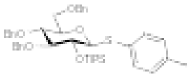

((2S,3S,4S,5R)-2-(p-tolylthio)-4,5-bis(benzyloxy)-6-((benzyloxy)methyl)-tetrahydro-2H-pyran-3-yloxy)triisopropylsilane

128

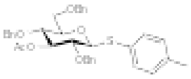

(2S,3S,4S,5R)-2-(p-tolylthio)-3,5-bis(benzyloxy)-6-((benzyloxy)methyl)-tetrahydro-2H-pyran-4-ylacetate

129

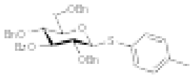

(2S,3S,4S,5R)-2-(p-tolylthio)-3,5-bis(benzyloxy)-6-((benzyloxy)methyl)-tetrahydro-2H-pyran-4-ylbenzoate

130

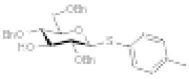

(2S,3S,4S,5S)-2-(p-tolylthio)-3,5-bis(benzyloxy)-6-((benzyloxy)methyl)-tetrahydro-2H-pyran-4-ol

131

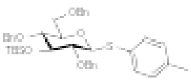

((2S,3S,4S,5R)-2-(p-tolylthio)-3,5-bis(benzyloxy)-6-((benzyloxy)methyl)-tetrahydro-2H-pyran-4-yloxy)(tert-butyl)dimethylsilane

132

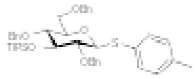

((2S,3S,4S,5R)-2-(p-tolylthio)-3,5-bis(benzyloxy)-6-((benzyloxy)methyl)-tetrahydro-2H-pyran-4-yloxy)triisopropylsilane

133

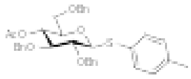

(3R,4S,5S,6S)-6-(p-tolylthio)-4,5-bis(benzyloxy)-2-((benzyloxy)methyl)-tetrahydro-2H-pyran-3-ylacetate

134

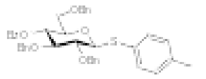

(3R,4S,5S,6S)-6-(p-tolylthio)-4,5-bis(benzyloxy)-2-((benzyloxy)methyl)-tetrahydro-2H-pyran-3-ylbenzoate

135

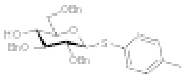

(3R,4S,5S,6S)-6-(p-tolylthio)-4,5-bis(benzyloxy)-2-((benzyloxy)methyl)-tetrahydro-2H-pyran-3-ol

136

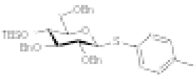

((3R,4R,5S,6S)-6-(p-tolylthio)-4,5-bis(benzyloxy)-2-((benzyloxy)methyl)-tetrahydro-2H-pyran-3-yloxy)(tert-butyl)dimethylsilane

137

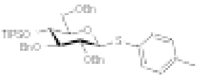

((3R,4R,5S,6S)-6-(p-tolylthio)-4,5-bis(benzyloxy)-2-((benzyloxy)methyl)-tetrahydro-2H-pyran-3-yloxy)triisopropylsilane

138

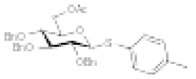

((3R,4S,5S,6S)-6-(p-tolylthio)-3,4,5-tris(benzyloxy)-tetrahydro-2H-pyran-2-yl)methylacetate

139

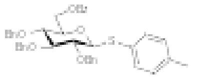

((3R,4S,5S,6S)-6-(p-tolylthio)-3,4,5-tris(benzyloxy)-tetrahydro-2H-pyran-2-yl)methylbenzoate

140

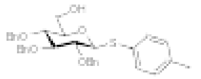

((3R,4S,5S,6S)-6-(p-tolylthio)-3,4,5-tris(benzyloxy)-tetrahydro-2H-pyran-2-yl)methanol

141

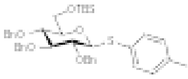

(((3R,4S,5S,6S)-6-(p-tolylthio)-3,4,5-tris(benzyloxy)-tetrahydro-2H-pyran-2-yl)methoxy)(tert-butyl)dimethylsilane

142

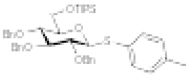

(((3R,4S,5S,6S)-6-(p-tolylthio)-3,4,5-tris(benzyloxy)-tetrahydro-2H-pyran-2-yl)methoxy)triisopropylsilane

143

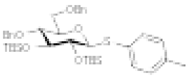

(((2S,3R,4S,5R,6R)-5-(benzyloxy)-6-((benzyloxy)methyl)-2-(p-tolylthio)tetrahydro-2H-pyran-3,4-diyl)bis(oxy))bis(tert-butyldimethylsilane)

144

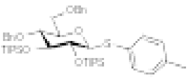

(((2S,3R,4S,5R,6R)-5-(benzyloxy)-6-((benzyloxy)methyl)-2-(p-tolylthio)tetrahydro-2H-pyran-3,4-diyl)bis(oxy))bis(triisopropylsilane)

145

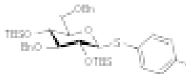

(((2R,3R,4S,5R,6S)-4-(benzyloxy)-2-((benzyloxy)methyl)-6-(p-tolylthio)tetrahydro-2H-pyran-3,5-diyl)bis(oxy))bis(tert-butyldimethylsilane)

146

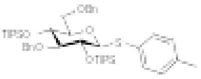

(((2R,3R,4S,5R,6S)-4-(benzyloxy)-2-((benzyloxy)methyl)-6-(p-tolylthio)tetrahydro-2H-pyran-3,5-diyl)bis(oxy))bis(triisopropylsilane)

147

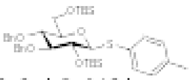

(((2R,3R,4S,5R,6S)-3,4-bis(benzyloxy)-5-(tert-butyldimethylsilyl)oxy)-6-(p-tolylthio)tetrahydro-2H-pyran-2-yl)methoxy)(tert-butyl)dimethylsilane

148

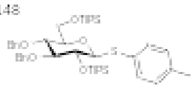

(((2S,3R,4S,5R,6R)-4,5-bis(benzyloxy)-2-(p-tolylthio)-6-(((triisopropylsilyl)oxy)methyl)tetrahydro-2H-pyran-3-yl)oxy)triisopropylsilane

149

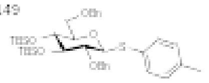

(((2R,3R,4R,5R,6S)-5-(benzyloxy)-2-((benzyloxy)methyl)-6-(p-tolylthio)tetrahydro-2H-pyran-3,4-diyl)bis(oxy))bis(tert-butyldimethylsilane)

150

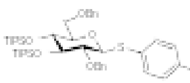

(((2R,3R,4R,5R,6S)-5-(benzyloxy)-2-((benzyloxy)methyl)-6-(p-tolylthio)tetrahydro-2H-pyran-3,4-diyl)bis(oxy))bis(triisopropylsilane)

151

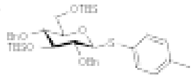

(((2R,3R,4S,5R,6S)-3,5-bis(benzyloxy)-2-(((tert-butyldimethylsilyl)oxy)methyl)-6-(p-tolylthio)tetrahydro-2H-pyran-4-yl)oxy)(tert-butyl)dimethylsilane

152

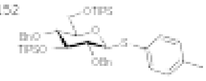

(((2S,3R,4S,5R,6R)-3,5-bis(benzyloxy)-2-(p-tolylthio)-6-(((triisopropylsilyl)oxy)methyl)tetrahydro-2H-pyran-4-yl)oxy)triisopropylsilane

153

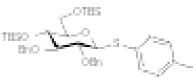

(((2R,3R,4R,5R,6S)-4,5-bis(benzyloxy)-2-(((tert-butyldimethylsilyl)oxy)methyl)-6-(p-tolylthio)tetrahydro-2H-pyran-3-yl)oxy)(tert-butyl)dimethylsilane

154

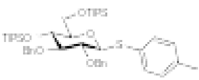

(((2R,3R,4R,5R,6S)-4,5-bis(benzyloxy)-6-(p-tolylthio)-2-(((triisopropylsilyl)oxy)methyl)tetrahydro-2H-pyran-3-yl)oxy)triisopropylsilane
