# Supplementary material for: Hierarchical and programmable one-pot synthesis of oligosaccharides
Source: Nat Commun. 2018 Dec 6;9:5202. doi: 10.1038/s41467-018-07618-8 (PMC6283847; doi:10.1038/s41467-018-07618-8)
Supplement: Supplementary file 2 — Peer Review File [file 41467_2018_7618_MOESM2_ESM.pdf]

Editorial Note: this manuscript has been previously reviewed at another journal that is not operating a transparent peer review scheme. This document only contains reviewer comments and rebuttal letters for versions considered at Nature Communications.

Reviewer #1 (Remarks to the Author):

The authors have addressed all the main points raised by this referee and made appropriate changes to the manuscript. I am happy to recommend publication – this is a significant piece of work and fully deserves to be published in Nature Communications. It will stimulate further work and discussions and with the open and accessible approach promised it will provide a valuable resource to researchers, both in carbohydrate chemistry but also to the broader chemical community.

Reviewer #2 (Remarks to the Author):

My previous comments have been well addressed, and the Nat Comm is appropriate place for the publishment.
